# Multi-format open-source sweet orange leaf dataset for disease detection, classification, and analysis

**DOI:** 10.1016/j.dib.2024.110713

**Published:** 2024-07-06

**Authors:** Yousuf Rayhan Emon, Md Taimur Ahad, Golam Rabbany

**Affiliations:** Daffodil International University, Daffodil Smart City (DSC), Birulia, Savar, Dhaka 1216, Bangladesh

**Keywords:** Sweet orange leaf, Diseases detection, Machine learning, Deep learning, Image classification, Computer vision, Plant pathology

## Abstract

In Bangladesh, sweet orange cultivation has been popular among fruit growers as the fruit is in demand. However, the disease of sweet oranges decreases fruit production. Research suggests that computer-aided disease diagnosis and machine learning (IML) models can improve fruit production by detecting and classifying diseases. In this line, a dataset of sweet oranges is required to diagnose the disease. Moreover, like many other fruits, sweet *orange* disease may vary from country to country. Therefore, in Bangladesh, a *sweet orange* dataset is required. Lastly, since different ML algorithms require datasets in various formats, only a few existing datasets fulfil the necessity. To fulfil the limitations, a sweet orange dataset in Bangladesh is collected. The dataset was collected in August and comprises high-quality images documenting multiple disease conditions, including *Citrus Canker, Citrus Greening, Citrus Mealybugs, Die Back, Foliage Damage, Spiny Whitefly, Powdery Mildew, Shot Hole, Yellow Dragon, Yellow Leaves, and Healthy Leaf*. These images provide an opportunity to apply machine learning and computer vision techniques to detect and classify diseases. This dataset aims to help researchers advance agri engineering through ML. Other sweet orange growing countries with having similar environments may find helpful information. Lastly, such experiments using our dataset will assist farmers in taking preventive measures and minimising economic losses.

Specifications Table

**Table:** 

Subject	Agriculture Engineering, Computer Science, Computer Vision and Pattern Recognition, Artificial Intelligence, Data Science
Specific subject area	Deep learning-based image detection and classification of plant leaf disease. (YOLO, Vision Transformer)
Data format	Raw: jpg Annotation: TXT
Type of data	Image
Data collection	This study presents a healthy and diseased leaf dataset of sweet oranges. The diseased leaves represent *Citrus Canker, Citrus Greening, Citrus Mealybugs, Die Back, Foliage Damage, Spiny Whitefly, Powdery Mildew, Shot Hole, Yellow Dragon,* and *Yellow Leaves* diseases. The images were collected from - Khemerdia, Bheramara, Kushtia district of Bangladesh, pinpointed at geographical coordinates 35.3602°N and 113.9505°E. This primary data was collected from August 19 to August 22, 2023. Two iPhones (Brand name) were used to collect the data from the field. The images were saved in JPEG format as JPEG image files are very efficient at compressing images with areas of solid colour, like photos. The dimensions of the images were chosen 3024×4032 pixels. After the initial collection, another collection of images of 640×480 pixels was produced. Moreover, the resolution was originally 72 dpi. The images were classified into diseases with the help of a plant pathology specialist. In total, eleven diseases or classes, namely: *Citrus canker, Citrus greening, Citrus mealybugs, Dieback, Foliage damage, Spiny whitefly, Powdery mildew, shot hole, yellow dragon, Yellowing leaves,* and *Healthy* were found in the collected dataset. This classification process resulted in a total of 5,813 images. From this collection, a subset was manually annotated so that instant segmentation which is the process of separating objects in an image, and it could be conducted using CNN. These annotations were converted into TXT formats using *the Makesenseai* web-based annotation tool*.*
Data source location	City: Khemerdia, Bheramara, Kushtia Country: Bangladesh Latitude and longitude (and GPS coordinates, if possible) for collected samples/data: 35.3602°N and 113.9505°E, Altitude: 75 msl
Data accessibility	Repository name: Mendeley Data Data identification number: 10.17632/f7cr74mwpj.1 Direct URL to data: https://data.mendeley.com/datasets/f7cr74mwpj/1

## 1 Value of the Data

• The dataset offers a collection of images of healthy and diseased sweet orange leaves. The main value of the dataset is that it can be used for machine learning models, which are expected to advance computer-aided plant disease diagnosis [1]. The dataset might be valuable to sweet-orange farmers from other countries with similar sweet-orange growing weather.
• The dataset covers a large number of classes (eleven in particular) including nine diseases of sweet orange leaf conditions.
• Using the dataset, data scientists and industry practitioners may find a solution that will assist farmers in making proper decisions to prevent disease. Thus, this dataset may help increase the production of sweet oranges and predict harvest yield, helping farmers optimise harvest timing.
• This dataset has practical applications. Using the dataset, mobile phone-based machine learning models can be implemented.
• The dataset has been made publicly accessible to support open research. This allows fellow researchers to use it in their machine-learning systems without requiring extensive validation or preprocessing.

## 2 Background

Sweet orange is an excellent source of Vitamin C, and it is an affordable fruit for ordinary people in Bangladesh. Therefore, the fruit is in demand in Bangladesh. However, sweet orange production is hampered by disease [2]. Research from the machine learning perspective suggests that the application of machine learning models for detecting plant diseases from leaf images is a promising way to yield the production of sweet oranges. Machine learning-based plant leaf disease detection requires noise-free, well-captured, large, well-annotated datasets. From the existing literature, a lack of publicly available sweet orange datasets in the Bangladeshi context is a motivating factor to collect the dataset [3,4]. This dataset aims to present sweet orange healthy and diseased leaves in raw and annotated format that can be utilised in feature engineering, convolutional neural network (CNN), and you only look once model. The datasetʼs compatibility with CNN architectures ensures its potential usefulness within current methodological trends in image-based agricultural development. Hence, we think it is imperative to develop and release such a dataset to foster research in machine learning-based plant disease detection. Top machine learning scientists and practitioners often opine that the benefit of this discipline is not yet fully harnessed for social goods such as healthcare and agriculture. So, our venture is to prepare a standard agricultural dataset that will leap forward, however small, the endeavour of sharing the benefit of machine learning for mass people.

## 3 Data Description

The dataset offers a collection of images of sweet orange healthy and diseased leaves from sweet orange growing gardens in Khemerdia, Bheramara, Kushtia, Bangladesh. The dataset includes a total of 5813 photographs. Researchers can use these images to study sweet orange leaf diseases. The main directory, labelled “sweet orange,” is categorised into three subdirectories. Each serves a unique purpose:

*Raw Images:* This directory stores photographs straight from the capturing device. The data are resized to ensure uniformity.

*Pixel-Modified Images:* In this section, images from the raw directory have been further processed to modify their pixel format. The images here are sorted into eleven distinct classes, facilitating easier categorisation and research.

*Annotations:* Annotations are crucial for machine learning and deep learning applications. In the sweet orange dataset, annotations are available in TXT formats. By choosing the txt format, the data accessibility, longevity, size efficiency, and software compatibility – factors that ensure annotated data remains usable, shareable, and easily processable. Other formats like XML, JSON, or proprietary binary formats may offer additional features like hierarchical data structures, metadata storage, or optimised parsing. However, these formats often have trade-offs, such as increased file sizes, potential compatibility issues across different software or versions, and the risk of format obsolescence over time (Table 1).

**Table 1:** Brief description of the data collection.

No.	Particulars	Description
1	Leaves	Sweet Orange
2	The number of diseases considered in the datatset	11
3	Data/image capturing time	19 August— to 22 August 2023, shooting every day. The image capturing the climate was sunny and captured in the daytime.
4	Geographical location	35.3602°N and 113.9505°E
5	Climatic	Sunny day
6	Temperature	27-29°C

This dataset holds immense potential for diagnosing sweet orange leaf diseases through visual recognition. Training machine learning models to automatically detect and classify various foliar diseases. Assisting agriculturalists in early disease detection and management (Table 2).

**Table 2:** Description of the dataset for sweet orange leaf (raw image).

Class	Citrus Canker	Citrus Greening	Citrus Mealy bugs	Die Back	Foliage Damaged	Spiny Whitefly	Powdery Mildew	Shot Hole	Yellow Dragon	Yellow Leaves	Health Leaves
No of Images	588	254	603	642	632	672	598	560	407	310	547

## 4 Disease Description

### 4.1 In the following sections, descriptions of the diseases are provided

#### 4.1.1 Citrus canker

**Fig 1:**
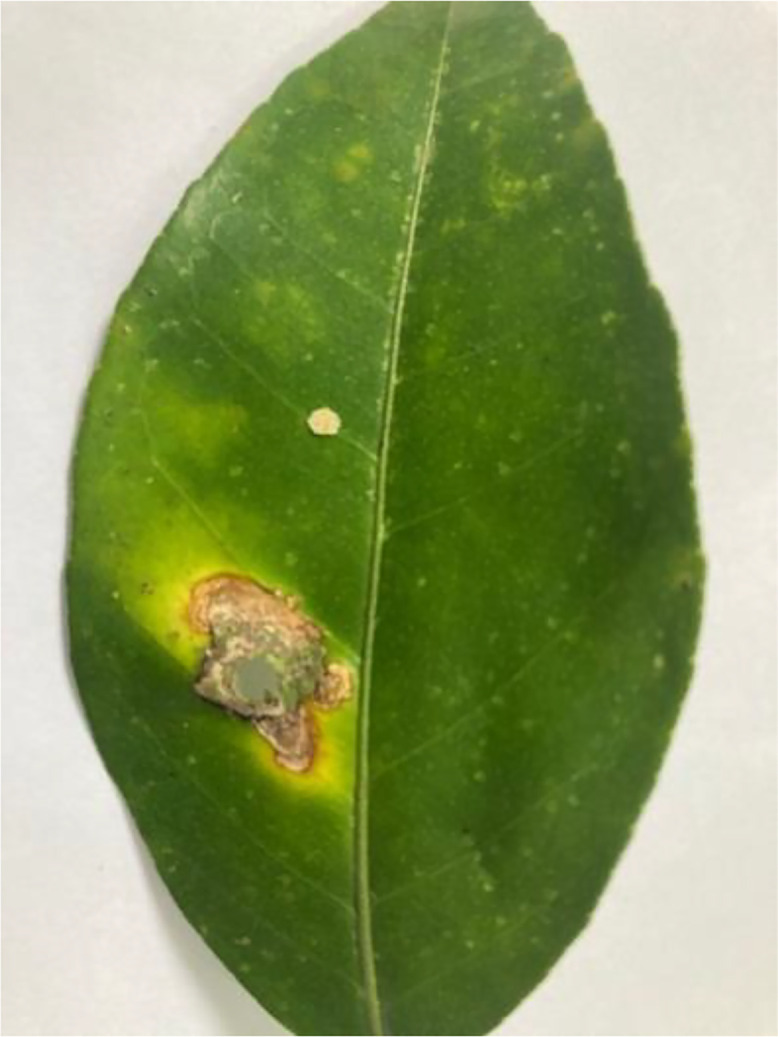
The citrus canker disease of sweet orange.

Citrus canker (see Fig. 1) is a bacterial disease caused by *Xanthomonas citri subsp. citri*. Citrus trees affected by this disease show raised, rough patches on their fruits, leaves, and stems. These patches look wet and might turn from tan to brown as time passes. Strong winds and rain can spread the disease faster, leading to poorer fruit quality and less fruit production. Although the tree doesn't die from this, the disease can cause leaves to fall, fruits to drop early, and spots on the fruit, making them unsuitable for sale [5] (Table 3).

**Table 3:** Description of the dataset for sweet orange leaf (annotation).

Class	Citrus Canker	Citrus Greening	Citrus Mealy bugs	Die Back	Foliage Damaged	Spiny Whitefly	Powdery Mildew	Shot Hole	Yellow Dragon
N of images	100	100	100	100	100	100	100	100	100

#### 4.1.2 Citrus greening

**Fig 2:**
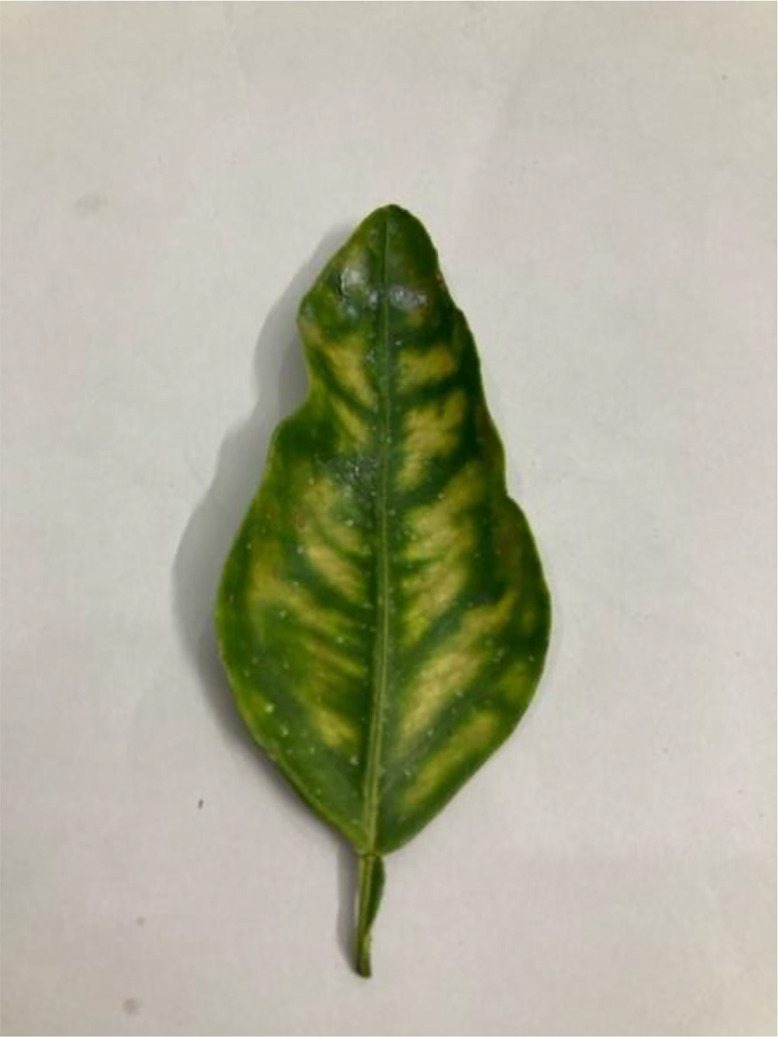
The citrus greening disease of sweet orange.

This disease, caused by the bacterium *Candidatus Liberibacter asiaticus*, is arguably the most devastating citrus disease globally (see Fig. 2). Transmitted by the Asian citrus psyllid, it results in asymmetrical yellowing and blotchy mottling of leaves, lopsided and bitter fruits, and an overall tree decline [6]. The root system also deteriorates, and trees eventually die. There is currently no cure for HLB (Table 4).

**Table 4:** Brief description of the dataset.

No	Particulars	Description
1	Leaf	Sweet Orange
2	Classes	11
3	Original Image	JPEG; 3024*4032 pixels: 72 dpi
4	Converted Image	640*480 pixels; 72 dpi
5	Annotation file format	TXT
6	Original Dataset size	Size of each image: 430-1008 KB Original Image folder size:3.69 GB
7	Converted Dataset size	Size of each image: 23-80 KB Original Image folder size: 263.8 MB
8	Annotated Dataset size	Annotations folder size: 2.02 GB (Includes Images and Txt files)

#### 4.1.3 Citrus mealybugs

**Fig 3:**
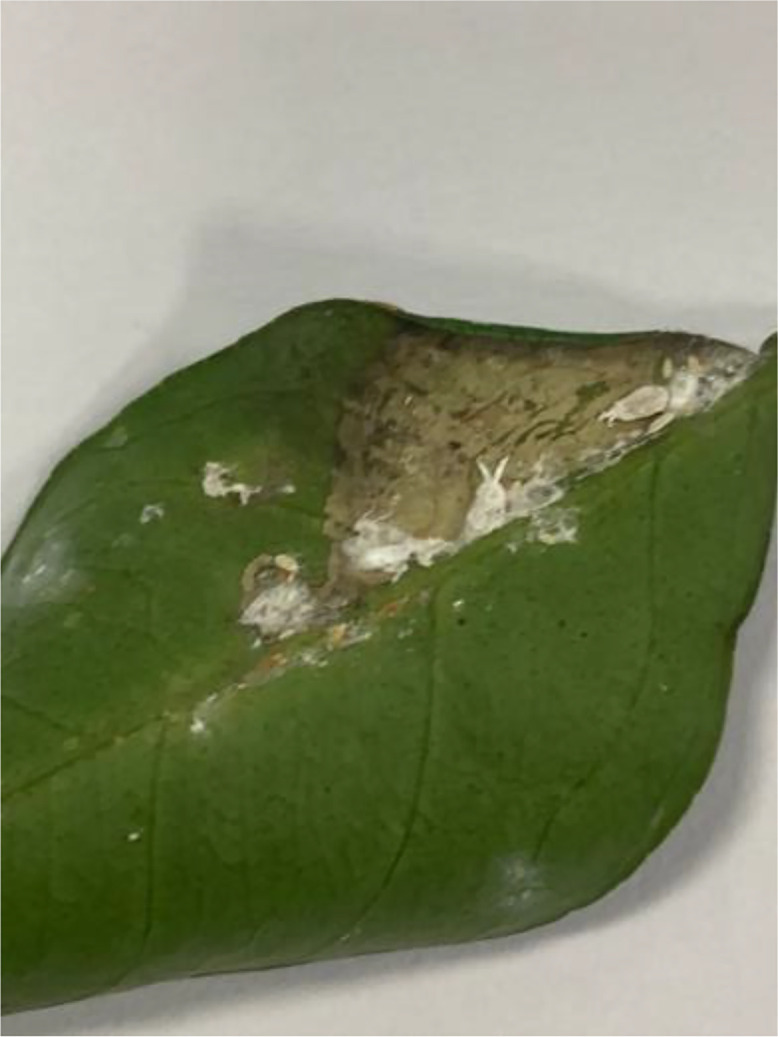
An image of citrus mealybugs disease of sweet orange.

Citrus mealybugs are soft-bodied insects covered with a white, waxy material, giving them a cottony appearance (see Fig. 3). These pests primarily reside on the undersides of leaves and stems of citrus plants, feeding on plant sap. Their feeding can result in yellowing of leaves, premature leaf drop, and growth stunting. Moreover, they excrete a sticky substance called honeydew, which promotes the growth of sooty mold, further impacting the health and aesthetics of the citrus plant [7].

#### 4.1.4 Die back

**Fig 4:**
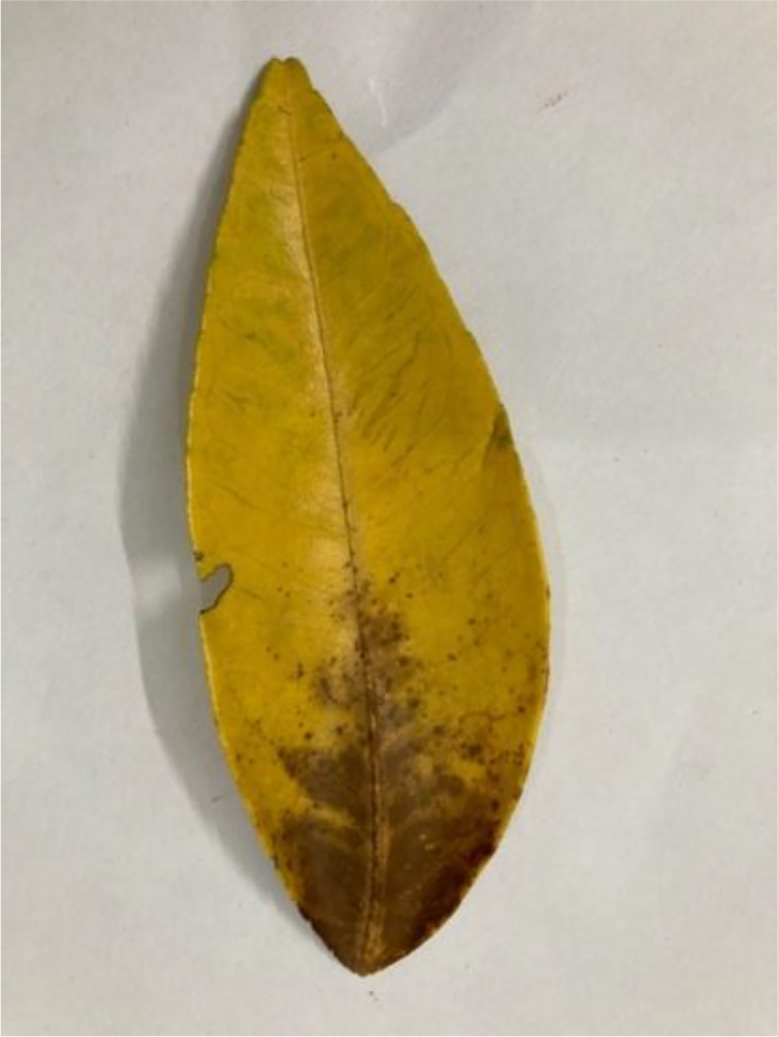
An image of die back disease of sweet orange.

Dieback in citrus is a complex syndrome often related to fungal infections, such as those caused by *Phomopsis* and *Botryosphaeria* species (see Fig. 4). Affected trees show twig and branch dieback, starting from the tip. Advanced infections can lead to a significant reduction in yield and tree vitality [8].

#### 4.1.5 Foliage damaged

**Fig 5:**
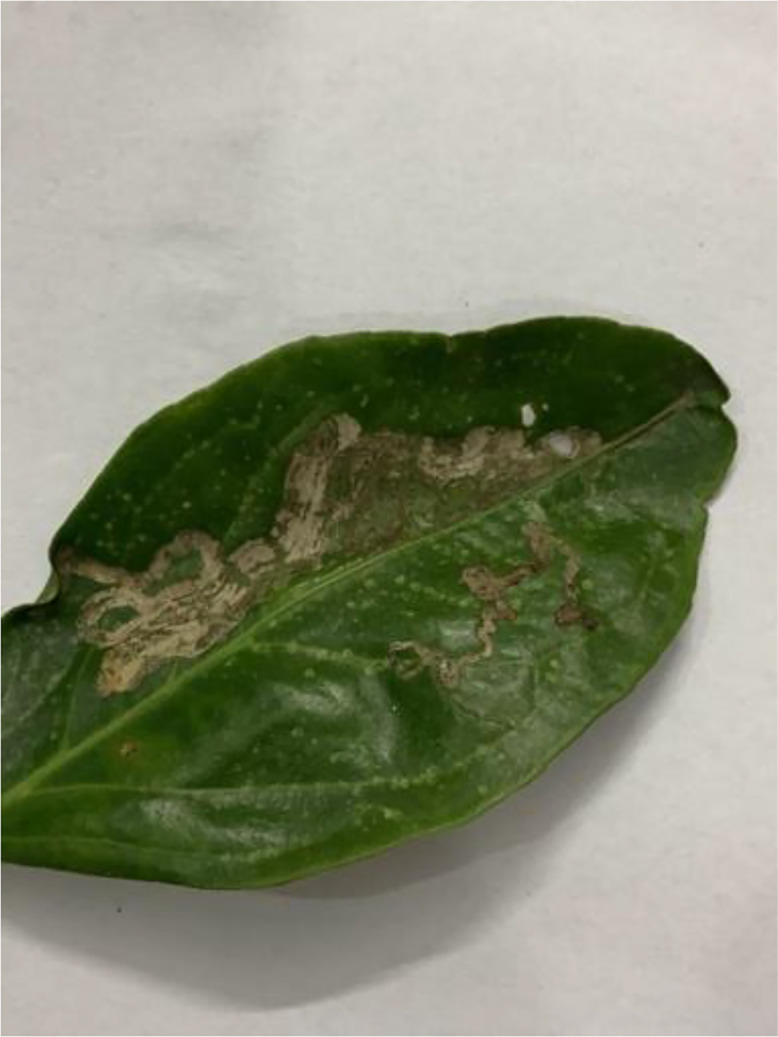
An image of foliage damaged disease of sweet orange.

Various factors, including fungal pathogens, pests, nutritional imbalances, or environmental stressors, can cause foliage damage. Affected leaves might show spots, curling, yellowing, or drop prematurely (see Fig. 5). Addressing the underlying cause is essential for managing this issue [9].

#### 4.1.6 Spiny whitefly

**Fig 6:**
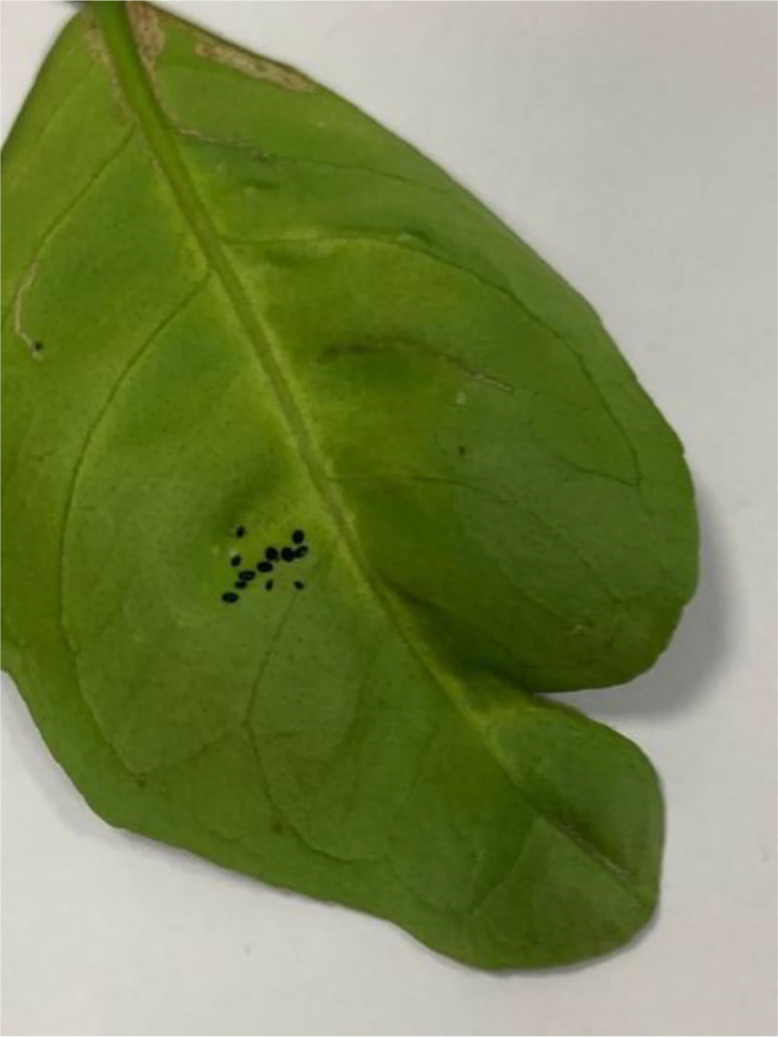
An image of spiny whitefly disease of sweet orange.

The Spiny Whitefly is a major pest of citrus and other fruit trees. Adults have yellowish bodies and wings that are covered with a white, waxy secretion (see Fig. 6). The nymphs, found on the undersides of leaves, are flat, oval, and surrounded by white, waxy filaments with prominent spines. Both nymphs and adults feed on plant sap, leading to symptoms like leaf curling, yellowing, and stunted growth. Like mealybugs, Spiny Whitefly excretes honeydew, facilitating the growth of sooty mould on leaves. This pest can cause significant damage if left uncontrolled [10].

#### 4.1.7 Powdery mildew

**Fig 7:**
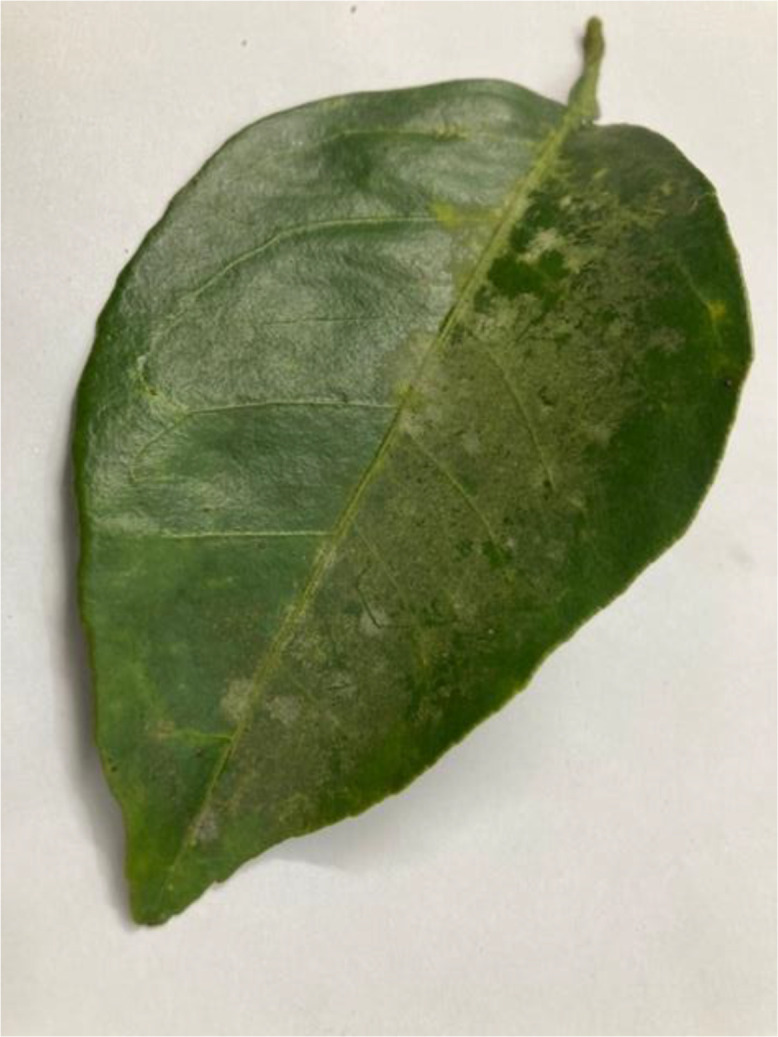
An image of powdery mildew disease of sweet orange.

This disease manifests as white powdery spots primarily on leaves. Over time, heavy infections may lead to leaf curling and drop. Caused by *Oidiopsis taurica*, it affects photosynthesis and fruit quality (see Fig. 7). Management includes fungicide applications and maintaining good airflow around trees [11].

#### 4.1.8 Shot hole

**Fig 8:**
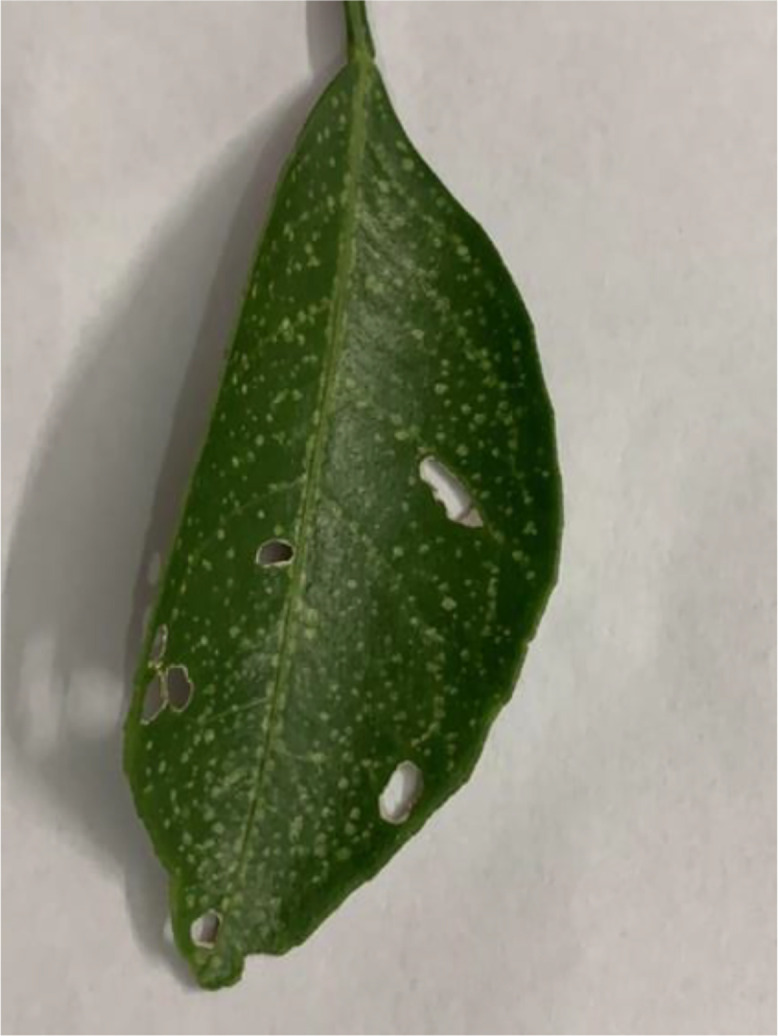
An image of shot hole disease of sweet orange.

This disease is often associated with a fungal pathogen that causes small, round holes to appear in the leaves, resembling pellet shots (see Fig. 8). Surrounding tissue may turn brown or yellow. Maintaining tree health is crucial to manage this disease [12].

#### 4.1.9 Yellow dragon

**Fig 9:**
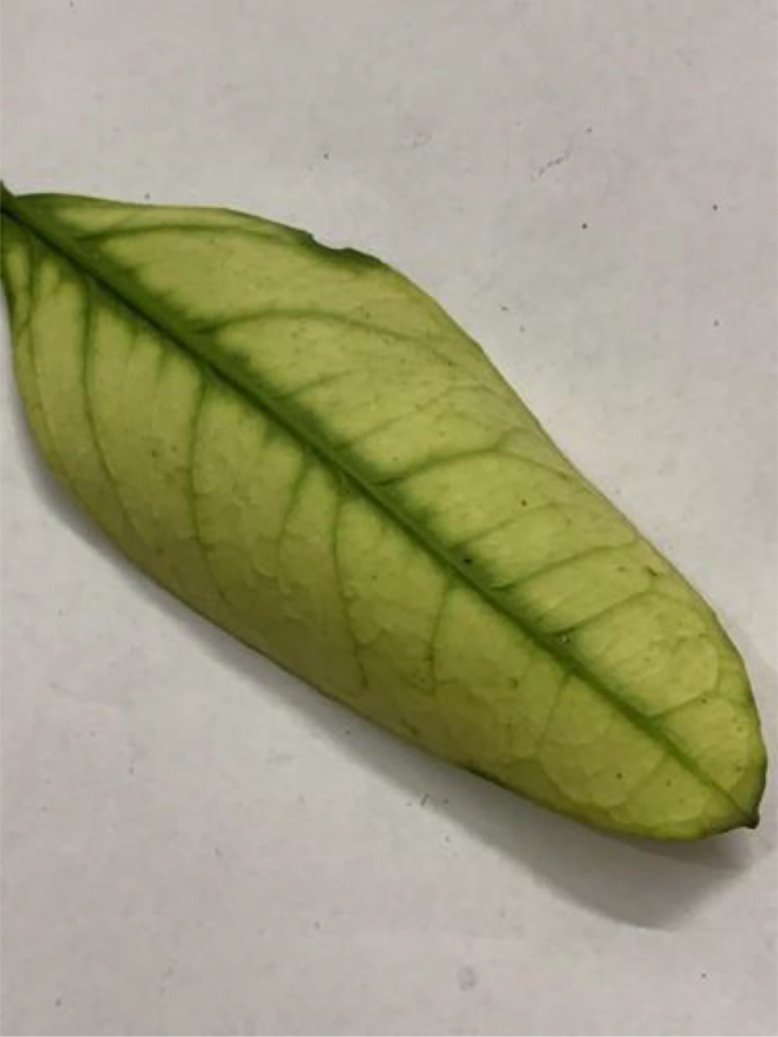
An image of yellow dragon disease of sweet orange.

Caused by the bacterium *Candidatus Liberibacter spp,* Yellow Dragon, also known as Huanglongbing (HLB) (see Fig. 9), is one of the most devastating citrus diseases, which is vectored by the Asian citrus psyllid (*Diaphorina citri*) and the African citrus psyllid (*Trioza erytreae*). Affected trees show symptoms such as blotchy mottling of leaves, yellowing of leaf veins, stunted growth, and the production of small, green, misshapen fruits with a bitter taste. The roots of infected trees also deteriorate over time [13].

#### 4.1.10 Yellow leaf

**Fig 10:**
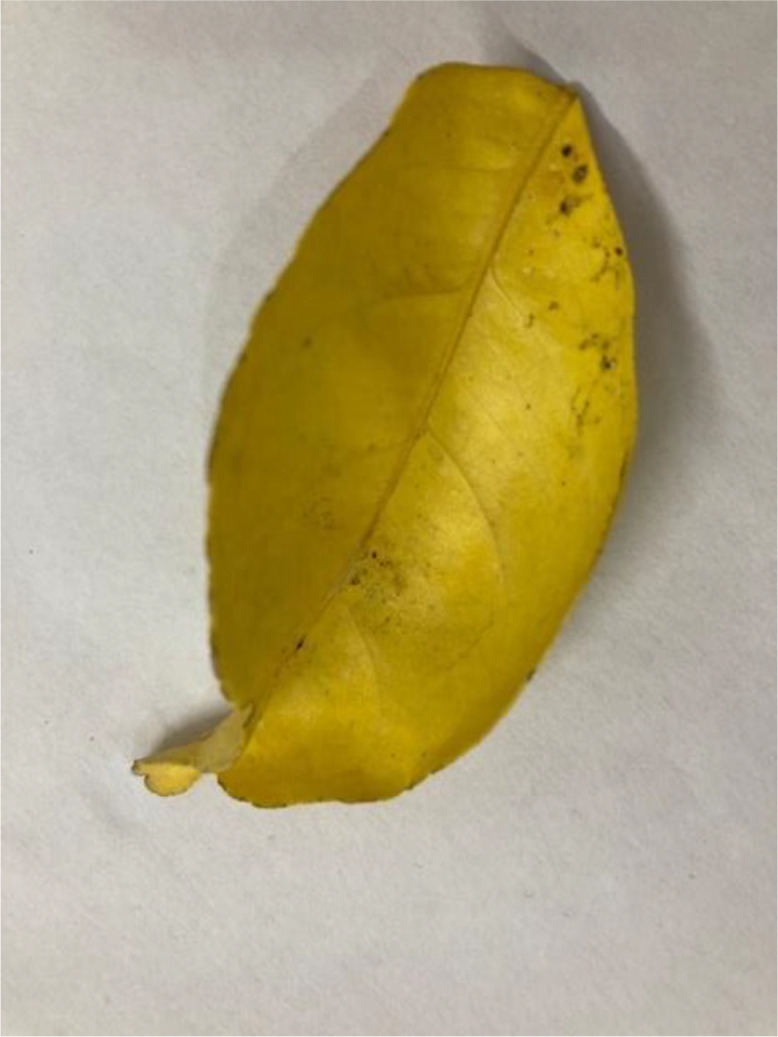
The yellow leaf of sweet orange.

Yellowing of citrus leaves can result from various issues, including nutrient deficiencies (e.g., nitrogen, iron, magnesium), water stress, root rot diseases, or infections like HLB (see Fig. 10). Comprehensive evaluation, including soil and tissue testing can help diagnose the cause [14].

#### 4.1.11 Healthy leaf

**Fig 11:**
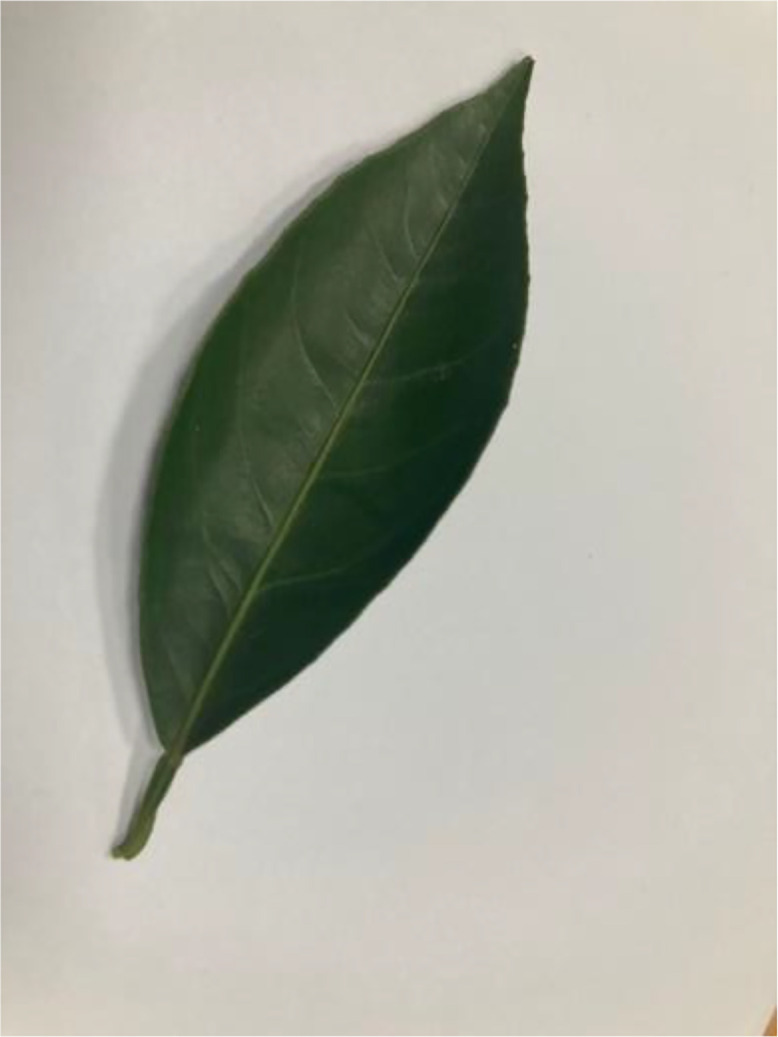
The healthy leaf of sweet orange.

Healthy sweet orange leaves are characterized by a vibrant green colour, smooth texture, and an elliptical shape (see Fig. 11). The leaves are typically firm to the touch, demonstrating good turgor pressure. There is an absence of any spots, blemishes, or discolouration indicating pest infestations or disease. The leaves are an essential indicator of the overall health of the citrus tree, playing a vital role in photosynthesis, which in turn supports fruit development and tree growth [15].

#### 4.1.12 Significance of the dataset

The Sweet Orange Leaf Diseases Dataset represents a collection of different types of diseases, from *Citrus Canker, Citrus Greening, Citrus Mealybugs, Die Back, Foliage Damage, Spiny Whitefly, Powdery Mildew, Shot Hole, Yellow Dragon, to Yellow Leaves*, along with images of healthy leaves for contrast. For the scientific community, particularly those in agronomy, plant pathology, and computational biology, this dataset offers an unparalleled opportunity for in-depth analysis and understanding of sweet orange leaf diseases. From a machine learning perspective, the dataset holds potential as a foundational resource for developing robust and accurate models for early disease detection, which is pivotal for timely interventions. For agronomists and farmers, the detailed categorisation of images enables more accurate and prompt disease diagnosis and more informed decision-making for disease detection. These proactive measures can mitigate yield losses and ensure consistent produce quality.

**Fig 12:**
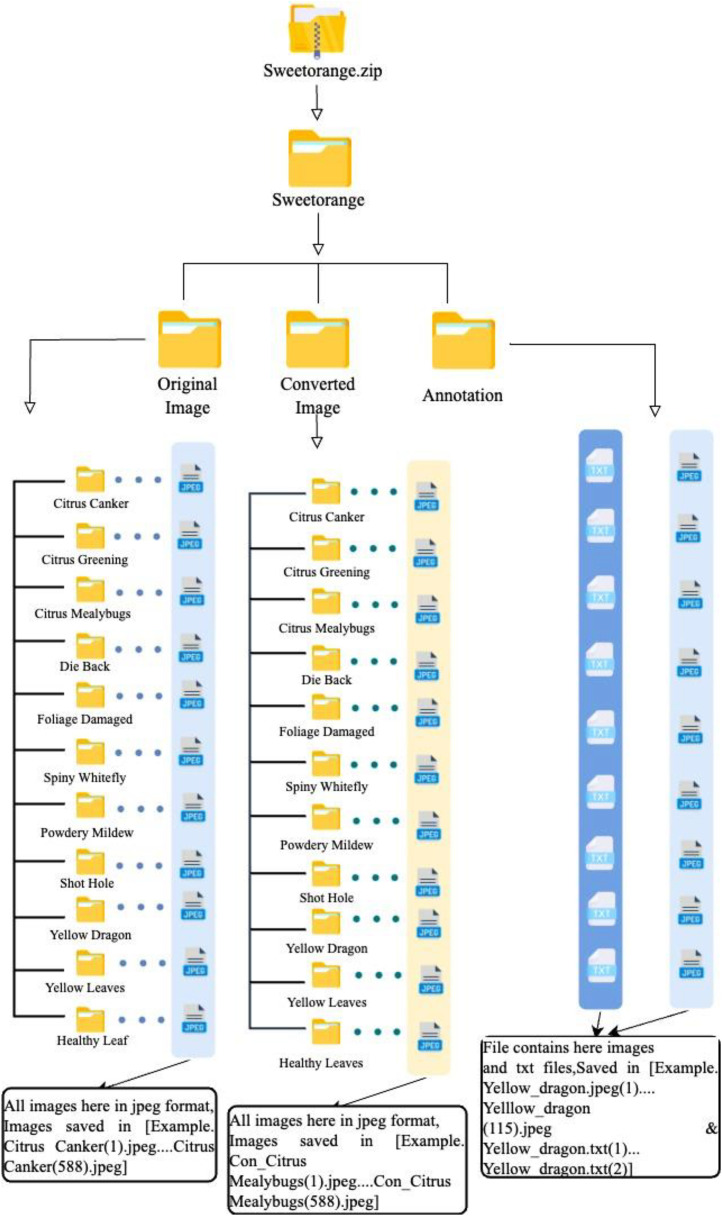
Folder hierarchy tree.

Sweet oranges are very important in many countries worldwide because they are a key part of their agricultural economies. The dataset highlights the need for computational approaches to boost sweet orange supply chains by recognising diseases (Fig. 12).

#### 4.1.13 Description of the dataset folder

In the root directory, there is only one file called Sweetorange.zip. Inside the folder, there is another folder called Sweet Orange, which contains all the dataset's subfolders. We have made a zip folder for easy download, and the Sweet Orange dataset folder has four subfolders: Original Images, Converted Images, Annotations, and Labels. You can find more information about each folder in Table 5.

**Table 5:** Brief description of the folders.

No	Folder Name	File Format in the folder	Description
1	Sweetorange.zip	zip (in itself)	Conveniently packaged for download
2	Sweet Orange	All format together	-
	Original Images	jpeg	Image files for easy viewing
3	Converted Images	jpeg	Image files help better experiment
4	Annotations	TXT	Annotated file with information such as category, location & size. The model can be used for training.

#### 4.1.14 Original images

The main folder contains all the images in the dataset, with a folder size of 3.69 GB. The images have varying sizes, with the highest 1008 KB and the lowest 430 KB. All images are in the 3024 × 4032-pixel and have a resolution of 72 dpi. This folder is divided into eleven subfolders, each containing images of specific classes such as Citrus Canker, Citrus Greening, Citrus Mealybugs, Die Back, Foliage Damaged, Spiny Whitefly, Powdery Mildew, Shot Hole, Yellow Dragon, and Yellow Leaves, as well as Healthy Leaves. We arranged the images this way to make it easier for researchers to find images based on their classification needs. Each image was renamed according to its class, such as Citrus Canker (1).jpeg to Citrus Canker (588).jpeg, Citrus Greening (1).jpeg to Citrus Greening (254).jpeg, Citrus Mealybugs (1).jpeg to Citrus Mealybugs (603).jpeg, Die Back (1).jpeg to Die Back (642).jpeg, Foliage Damaged (1).jpeg to Foliage Damaged (632).jpeg, Spiny Whitefly (1).jpeg to Spiny Whitefly (672).jpeg, Powdery Mildew (1).jpeg to Powdery Mildew (598).jpeg, Shot Hole (1).jpeg to Shot Hole (560).jpeg, Yellow Dragon (1).jpeg to Yellow Dragon (407).jpeg, Yellow Leaves (1).jpeg to Yellow Leaves (310).jpeg, Healthy Leaf (1).jpeg to Healthy Leaf (547).jpeg. This makes it easier for researchers to train their Sweet orange leaf disease classification model using the specific images they need (Table 6).

**Table 6:** Description of the camera devices.

No.	Particulars	Device 1	Device 2
		iPhone	iPhone
1.	Phone model	iPhone SE 2^nd^ Generation	iPhone XS Max
2.	Camera manufacturer	Apple	Apple
3.	Camera model	Single 12MP Wide camera	Dual 12MP Wide and Telephoto cameras
4.	Camera pixel	12MP	12MP
5.	Aperture Value	f/1.8	f/1.8
6.	Exposure time	1/60	1/60
7.	Camera flash	None	None
8.	Monitoring range	3.99 mm	4.25 mm
9.	Imaging	Size: 3024*4032 Resolution: 72 dpi Bit Depth:24	Size: 3024*4032 Resolution: 72 dpi Bit Depth:24

#### 4.1.15 Converted images

The Converted Images folder contains all the images in the dataset. The folder size is 263.8 MB. The highest image size of the image is 80 KB, and the lowest is 23 KB. All the images were in 3024*4032-pixel format, and the resolution was 72 dpi. The images were converted to 480*640-pixel format using the software ‘Microsoft Powertoys’. This folder is divided into eleven subfolders, each containing images of specific classes such as Citrus Canker, Citrus Greening, Citrus Mealybugs, Die Back, Foliage Damaged, Spiny Whitefly, Powdery Mildew, Shot Hole, Yellow Dragon, and Yellow Leaves, as well as Healthy Leaves. Each folder contains images of those classes individually. We've made the images in this structure due to classification help. Each image was renamed according to its class, such as Con_Citrus Canker (1).jpeg to Con_Citrus Canker (588).jpeg, Con_Citrus Greening (1).jpeg to Con_Citrus Greening (254).jpeg, Con_Citrus Mealybugs (1).jpeg to Con_Citrus Mealybugs (603).jpeg, Con_Die Back (1).jpeg to Con_Die Back (642).jpeg, Con_Foliage Damaged (1).jpeg to Con_Foliage Damaged (632).jpeg, Con_Spiny Whitefly (1).jpeg to Con_Spiny Whitefly (672).jpeg, Con_Powdery Mildew (1).jpeg to Con_Powdery Mildew (598).jpeg, Con_Shot Hole (1).jpeg to Con_Shot Hole (560).jpeg, Con_Yellow Dragon (1).jpeg to Con_Yellow Dragon (407).jpeg, Con_Yellow Leaves (1).jpeg to Con_Yellow Leaves (310).jpeg, Con_Healthy Leaf (1).jpeg to Con_Healthy Leaf (547).jpeg.

#### 4.1.16 Annotations

Annotation is the labelling and region of interest of an image. The annotation format of an image allows the detection, classification and grouping of images recognisable to machines through machine learning. Annotation files contain the bounding boxes in images for object detection tasks. In this format, each image in the dataset should have a corresponding text file with the same name as the image, containing the bounding box annotations for that image. The annotation folder contains 900 TXT files and 900 images. Each image was renamed according to its class, such as Citrus_canker (1).jpeg to Citrus_canker (100).jpeg and Citrus_canker (1).txt to Citrus_canker (100).txt.

## 5 Experimental Design, Materials and Methods

### 5.1 Theoretical knowledge gathering

Researchers should have theoretical knowledge of the crop and the disease of the crops. Therefore, an extensive literature review on sweet orange trees, specifically the diseases and the symptoms of the disease, was conducted. Academic research papers, agricultural websites, and specialized studies on sweet orange trees were the sources of the literature review. Leaf morphology, symptoms of diseases, diagnostic techniques, and symptomatology were studied to broaden the theoretical knowledge.

### 5.2 Fieldwork

After collecting knowledge about leaf diseases, the next step was the field trip to collect a dataset of the diseased leaves. In the fieldwork, the selection of trees and the selection of diseased leaves were important. The selection of trees was based on accessibility to the trees, availability of enough leaves, and maturity of the leaves. Trees were randomly selected, however focus was to have enough leaves to represent all the diseases we wanted to cover. Leaves that are matured and diseased were only collected for the dataset. Visibility of the disease in the leaves, leaves that have a significant portion diseased, and maturity of the leaf were some considerations while the leaves were collected.

### 5.3 Image capturing of the collected leaves in the fieldwork

**Fig 13:**
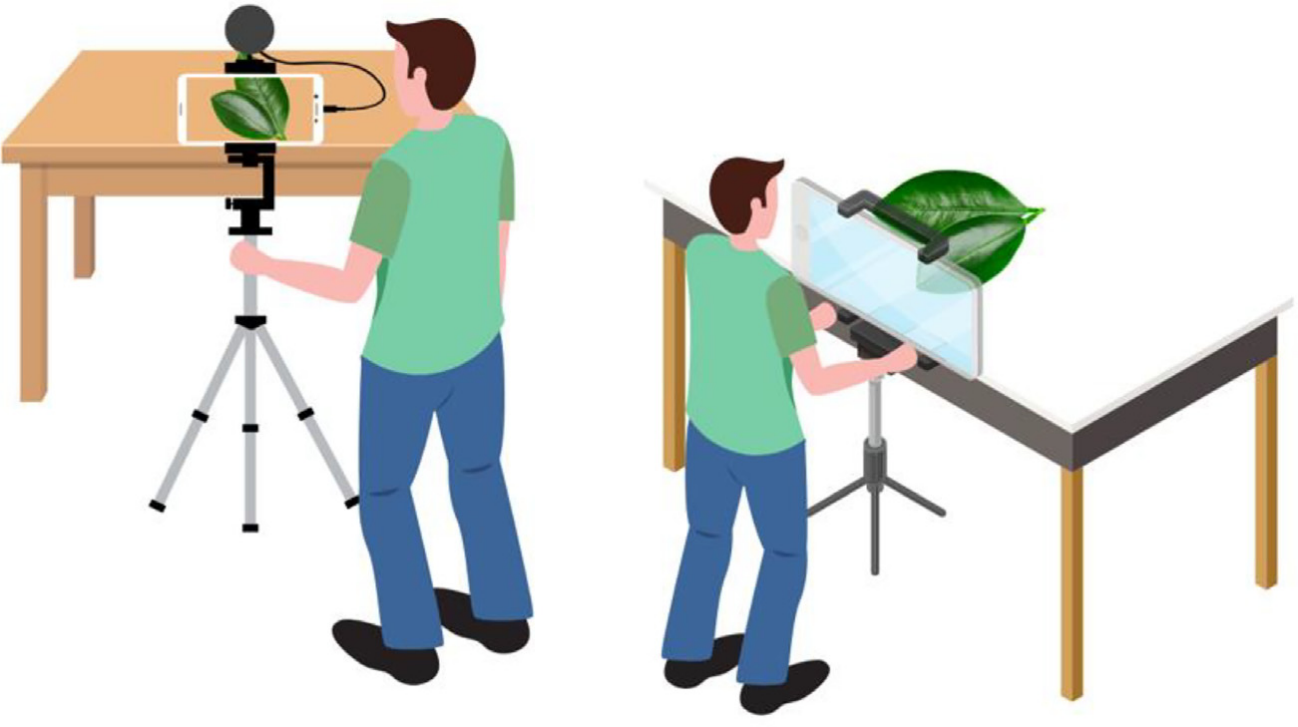
Photo capturing method of sweet orange leaf.

After the fieldwork, diseased leaves were sorted according to the classes. Eleven buckets were used for sorting; the buckets were labelled as per class (disease) names. The leaves were then placed on a white page. It was made sure that the collected leaves were not damaged or rotten. High-resolution pictures were considered and taken to avoid incorrect identification and classification. It is important to note that the effectiveness of machine learning models depends on the clarity and precision of the photographs. Therefore, during image collection, special attention was paid to this step (see Fig. 13).

Two devices, the iPhone SE 2022, and the iPhone XS Max was used to capture all the images of the collected leaves after fieldwork. Both devices captured an image size of 3024*4032 pixels with a 72-dpi resolution. Initially, images were taken in jpeg format.

• The device position was 2.5 feet from the ground, depending on the table position from the ground.
• Device distance from the target leaf: 30–40 cm.

### 5.4 Leaf classification

**Fig 14:**
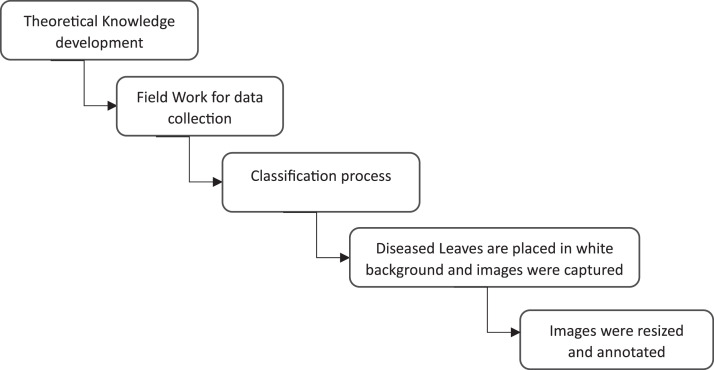
Image classification process.

To ensure the accuracy of the disease classification model, we sorted the collected leaves based on their corresponding disease. There are eleven (11) different disease classifications, namely: Citrus Canker, Citrus Greening, Citrus Mealybugs, Die Back, Foliage Damage, Spiny Whitefly, Powdery Mildew, Shot Hole, Yellow Dragon, Yellow Leaves, and Healthy Leaf. However, a representative from the Bangladesh Agriculture Association, who is an expert in this field, was included in the research team. The purpose was to apply his theoretical knowledge of sweet orange disease and made the disease class appropriate. The image collection process is described in Fig. 14. The process classification of diseased images is described in Fig. 14.

To collect the dataset, firstly, theoretical knowledge of the sweet orange disease was obtained Following the knowledge obtained, the fieldwork for data collection started in the Kushtia area of Bangladesh. After the image collection, the images were classified into designated classes. Diseased leaves were placed on a white background and images were captured.

### 5.5 Image processing

To make the images more manageable for researchers, the height and width of the images were decreased using *‘Microsoft Powertoys*. Microsoft Powertoys offers a convenient and user-friendly solution with integrated image resizer tool, allowing one to specify desired dimensions and output formats. Compared to the other image resizing tools such as Adobe Photoshop, BulkResizePhotos, and ResizePixel, Microsoft Powertoys is more user-friendly. Lastly, it is open source and works well in a Windows environment. The resulting image size was 640×480 pixels, with a resolution of 72 dpi. Additional information on image processing can be found in Section 2.3.

### 5.6 Image annotation

Image annotation is mandatory for specific deep learning models (such as YOLO, R-CNN, SSD, U-Net, and FCN). To make the dataset appropriate for these models, annotation was conducted, and the annotation files are included. From each class, 900 images were annotated. All the annotation files are in txt format.

## Limitations

Like many other studies, this research also has some limitations and opportunities for further improvements [[16], [17], [18]]. For example, one can complain that images are only in high resolution. In future, images might be collected using a low-resolution camera. The high-resolution and low-resolution datasets might be the source of further research experiments. Data scientists could find useful information while applying machine learning models on both high-resolution and low-resolution image sets. The dataset was collected in only one place. In future datasets can be collected from different regions in Bangladesh. Lastly, images could be captured in different weather and time which could enrich the features of the dataset.

## Ethics Statement

Neither plants nor animals contracted an infection while the data were being gathered. The current work does not use any human subjects, animal trials, or data gathered from social media sites. The authors strictly maintained the ethical code of data in brief during the experiment of dataset collection.

## CRediT authorship contribution statement

**Yousuf Rayhan Emon:** Investigation, Methodology, Writing – original draft, Visualization, Data curation, Resources. **Md Taimur Ahad:** Conceptualization, Supervision, Writing – review & editing. **Golam Rabbany:** Conceptualization, Writing – review & editing.

## Data Availability

Multi-format open-source sweet orange leaf dataset for disease detection, classification, and analysis (Original data) (Mendeley Data).

## Appendix Supplementary materials

Image, application 1
