# Supplementary figures and images for: Multi-format open-source sweet orange leaf dataset for disease detection, classification, and analysis (part 2 of 2)
Source: Data Brief. 2024 Jul 6;55:110713. doi: 10.1016/j.dib.2024.110713 (PMC11295629; doi:10.1016/j.dib.2024.110713)

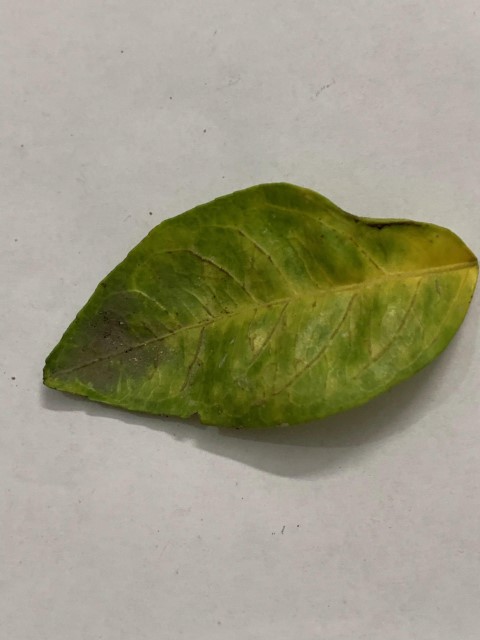

Supplement: Supplementary file 1 [file mmc1.zip › Sweetorange Sample Dataset/Converted Image/Powdery mildew/Con_Powdery Mildew35.jpeg]

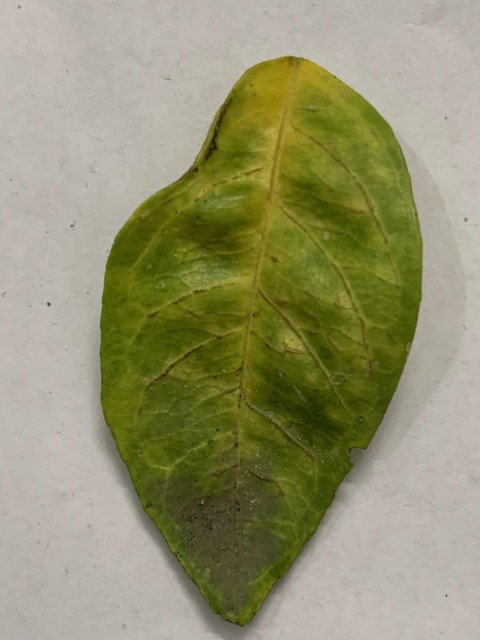

Supplement: Supplementary file 1 [file mmc1.zip › Sweetorange Sample Dataset/Converted Image/Powdery mildew/Con_Powdery Mildew42.jpeg]

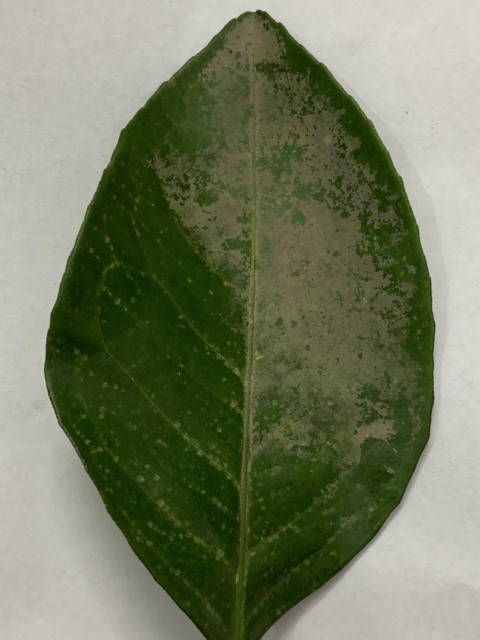

Supplement: Supplementary file 1 [file mmc1.zip › Sweetorange Sample Dataset/Converted Image/Powdery mildew/Con_Powdery Mildew14.jpeg]

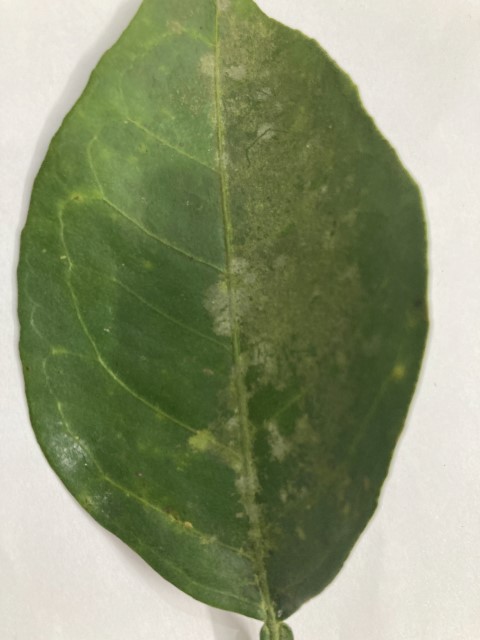

Supplement: Supplementary file 1 [file mmc1.zip › Sweetorange Sample Dataset/Converted Image/Powdery mildew/Con_Powdery Mildew560.jpeg]

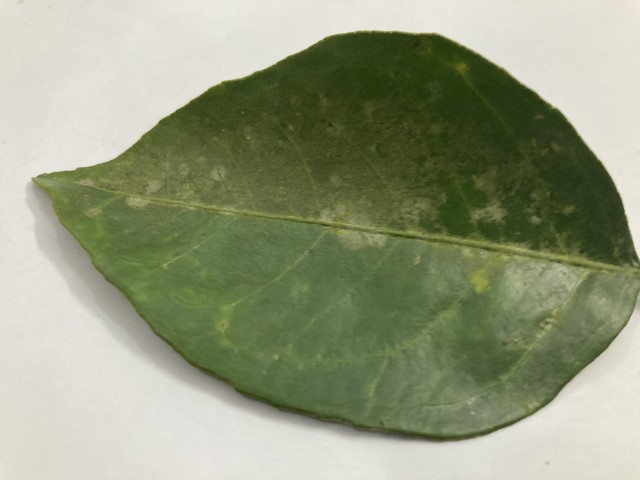

Supplement: Supplementary file 1 [file mmc1.zip › Sweetorange Sample Dataset/Converted Image/Powdery mildew/Con_Powdery Mildew448.jpeg]

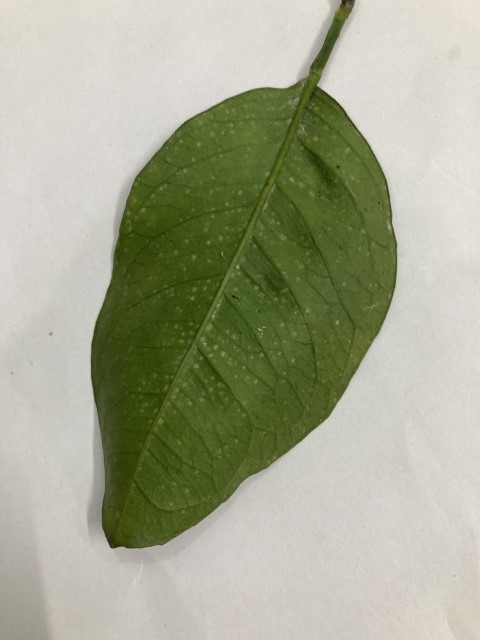

Supplement: Supplementary file 1 [file mmc1.zip › Sweetorange Sample Dataset/Converted Image/Powdery mildew/Con_Powdery Mildew595.jpeg]

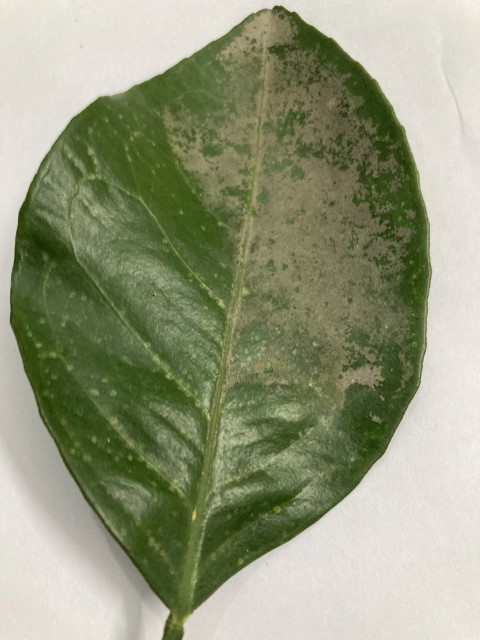

Supplement: Supplementary file 1 [file mmc1.zip › Sweetorange Sample Dataset/Converted Image/Powdery mildew/Con_Powdery Mildew63.jpeg]

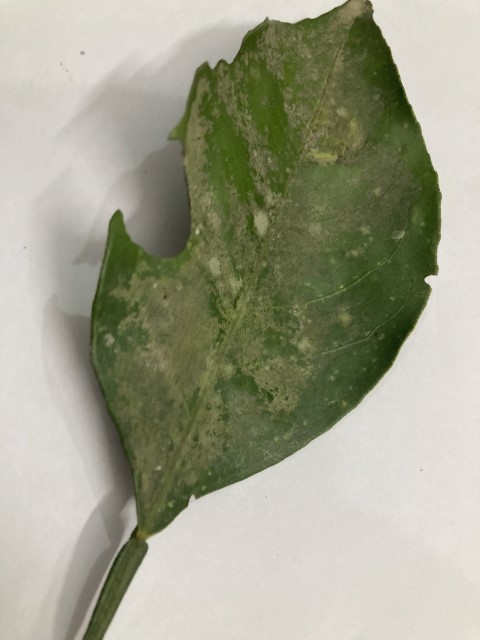

Supplement: Supplementary file 1 [file mmc1.zip › Sweetorange Sample Dataset/Converted Image/Powdery mildew/Con_Powdery Mildew567.jpeg]

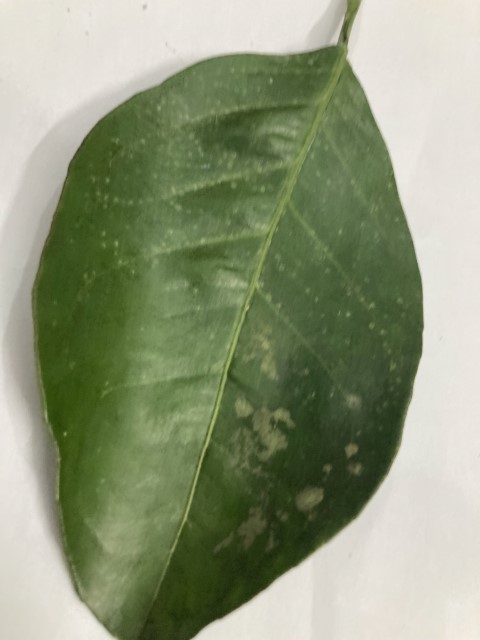

Supplement: Supplementary file 1 [file mmc1.zip › Sweetorange Sample Dataset/Converted Image/Powdery mildew/Con_Powdery Mildew588.jpeg]

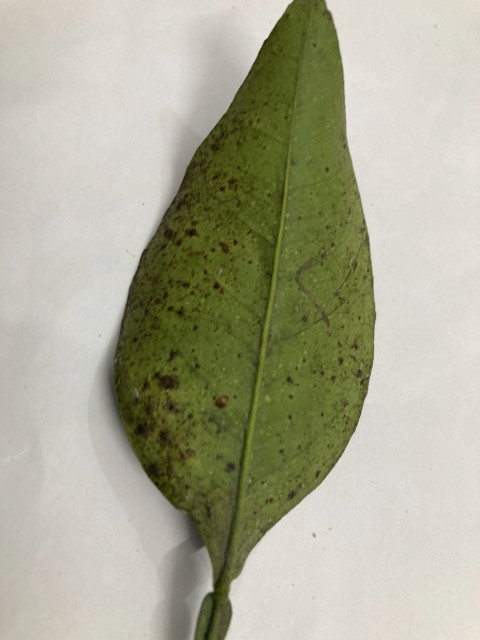

Supplement: Supplementary file 1 [file mmc1.zip › Sweetorange Sample Dataset/Converted Image/Powdery mildew/Con_Powdery Mildew434.jpeg]

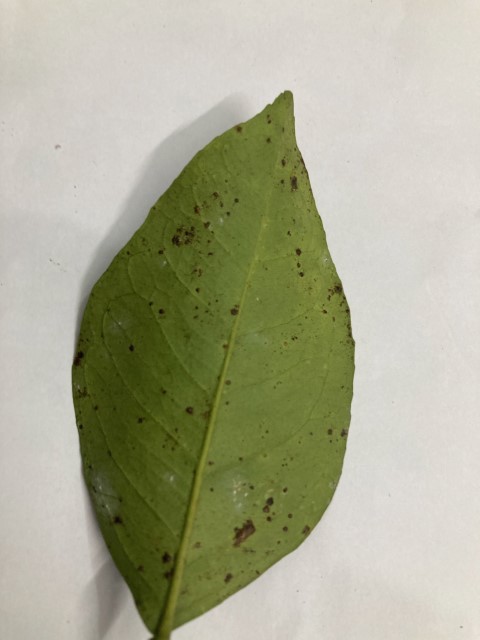

Supplement: Supplementary file 1 [file mmc1.zip › Sweetorange Sample Dataset/Converted Image/Powdery mildew/Con_Powdery Mildew455.jpeg]

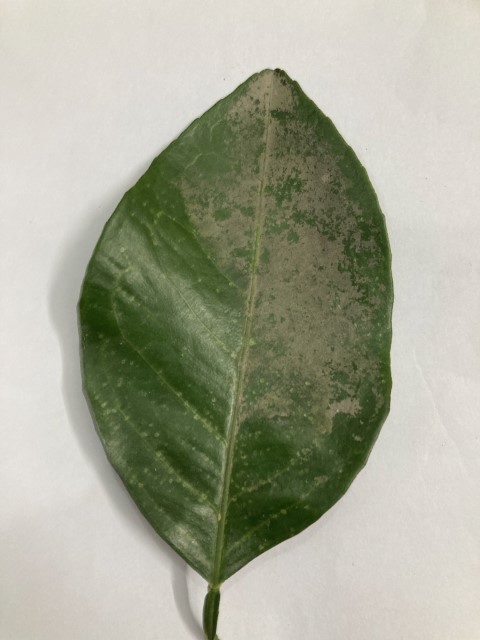

Supplement: Supplementary file 1 [file mmc1.zip › Sweetorange Sample Dataset/Converted Image/Powdery mildew/Con_Powdery Mildew49.jpeg]

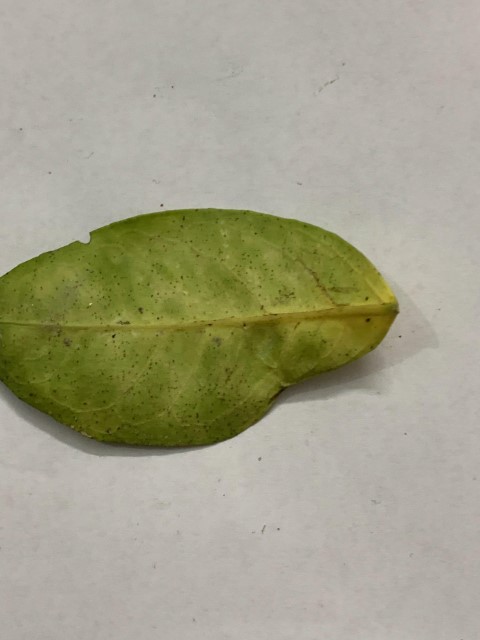

Supplement: Supplementary file 1 [file mmc1.zip › Sweetorange Sample Dataset/Converted Image/Powdery mildew/Con_Powdery Mildew28.jpeg]

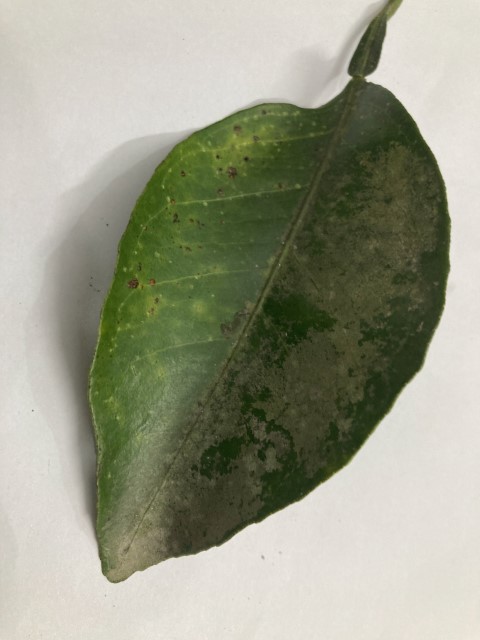

Supplement: Supplementary file 1 [file mmc1.zip › Sweetorange Sample Dataset/Converted Image/Powdery mildew/Con_Powdery Mildew420.jpeg]

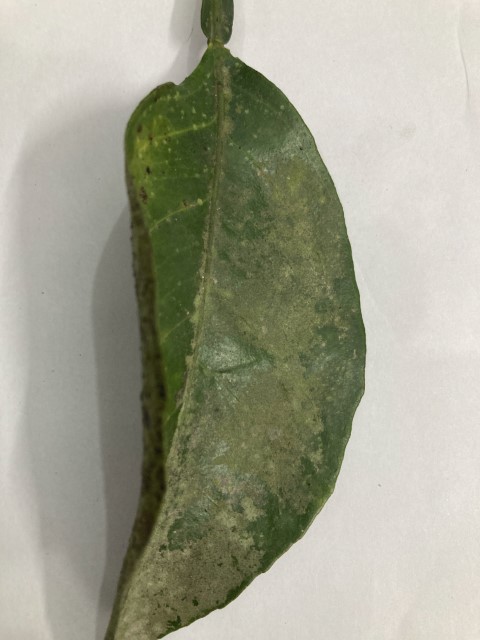

Supplement: Supplementary file 1 [file mmc1.zip › Sweetorange Sample Dataset/Converted Image/Powdery mildew/Con_Powdery Mildew553.jpeg]

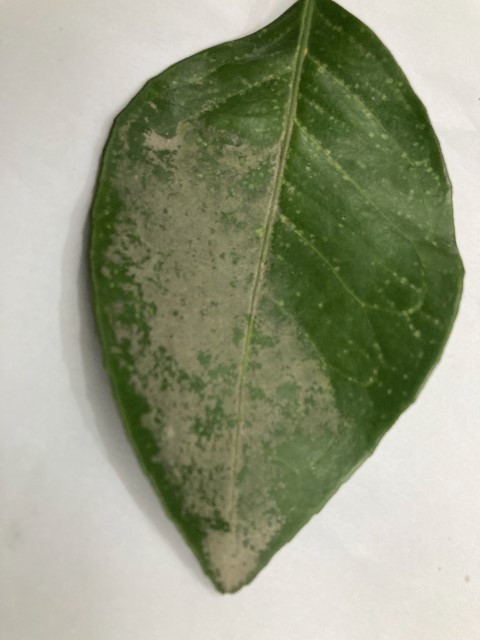

Supplement: Supplementary file 1 [file mmc1.zip › Sweetorange Sample Dataset/Converted Image/Powdery mildew/Con_Powdery Mildew70.jpeg]

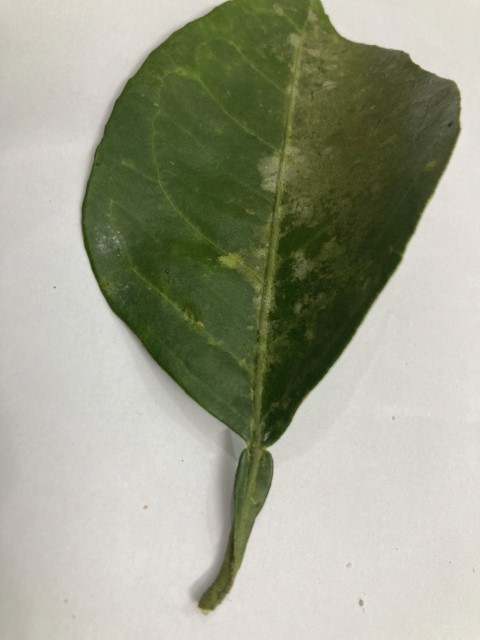

Supplement: Supplementary file 1 [file mmc1.zip › Sweetorange Sample Dataset/Converted Image/Powdery mildew/Con_Powdery Mildew441.jpeg]

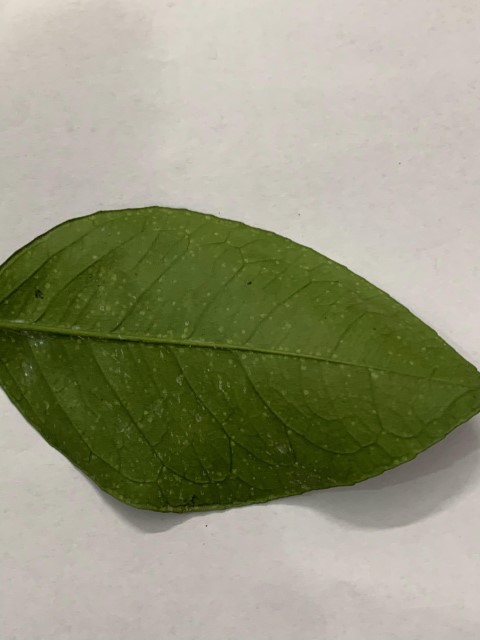

Supplement: Supplementary file 1 [file mmc1.zip › Sweetorange Sample Dataset/Converted Image/Powdery mildew/Con_Powdery Mildew21.jpeg]

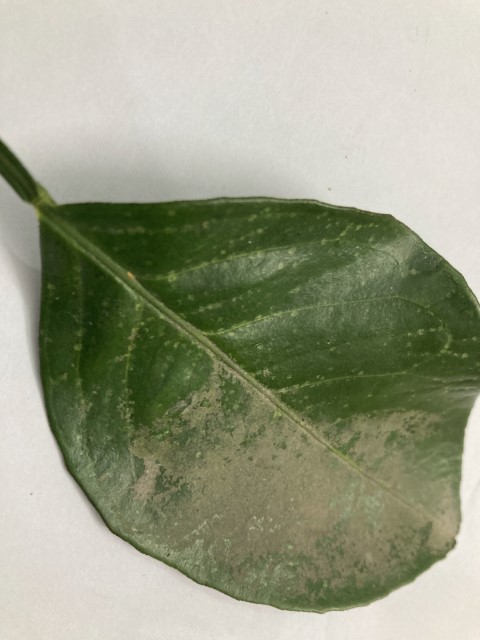

Supplement: Supplementary file 1 [file mmc1.zip › Sweetorange Sample Dataset/Converted Image/Powdery mildew/Con_Powdery Mildew56.jpeg]

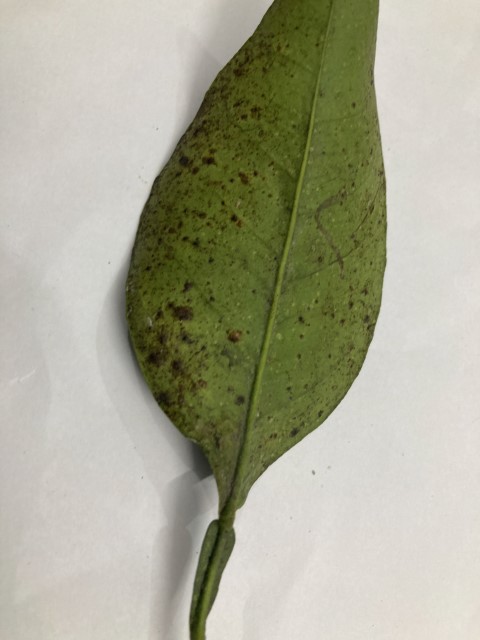

Supplement: Supplementary file 1 [file mmc1.zip › Sweetorange Sample Dataset/Converted Image/Powdery mildew/Con_Powdery Mildew427.jpeg]

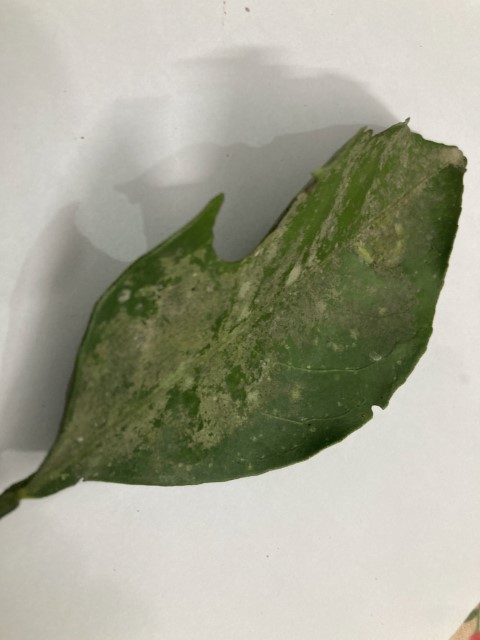

Supplement: Supplementary file 1 [file mmc1.zip › Sweetorange Sample Dataset/Converted Image/Powdery mildew/Con_Powdery Mildew574.jpeg]

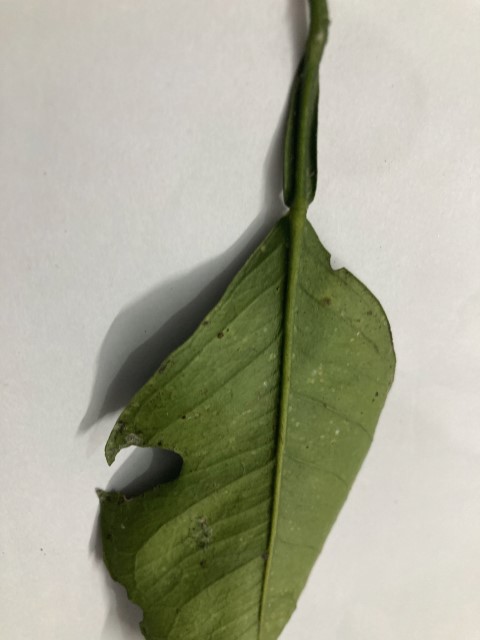

Supplement: Supplementary file 1 [file mmc1.zip › Sweetorange Sample Dataset/Converted Image/Powdery mildew/Con_Powdery Mildew581.jpeg]

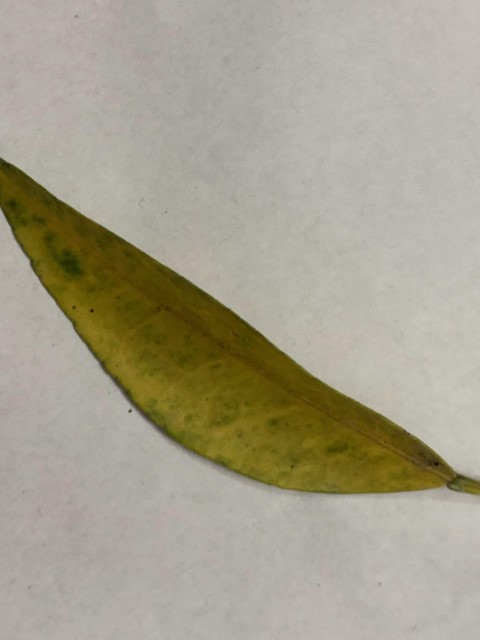

Supplement: Supplementary file 1 [file mmc1.zip › Sweetorange Sample Dataset/Converted Image/Yellow leaves/Con_Yellow Leaves7.jpeg]

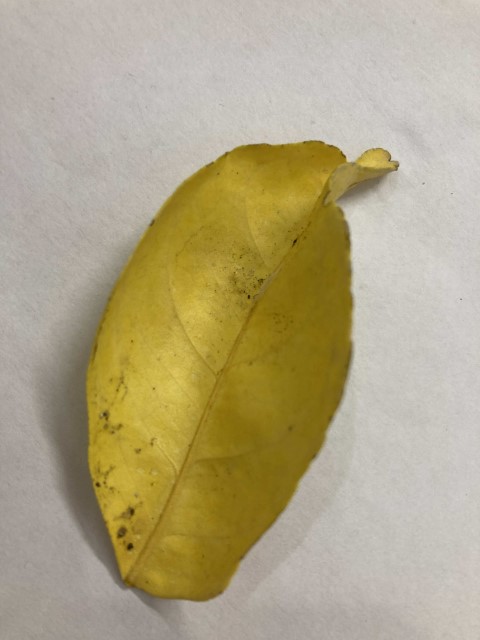

Supplement: Supplementary file 1 [file mmc1.zip › Sweetorange Sample Dataset/Converted Image/Yellow leaves/Con_Yellow Leaves49.jpeg]

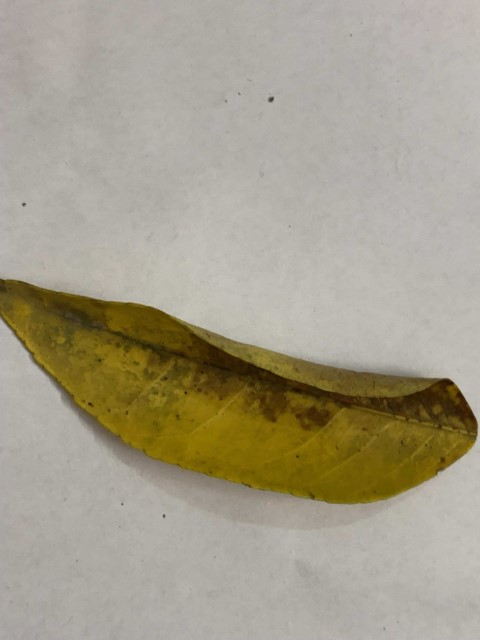

Supplement: Supplementary file 1 [file mmc1.zip › Sweetorange Sample Dataset/Converted Image/Yellow leaves/Con_Yellow Leaves28.jpeg]

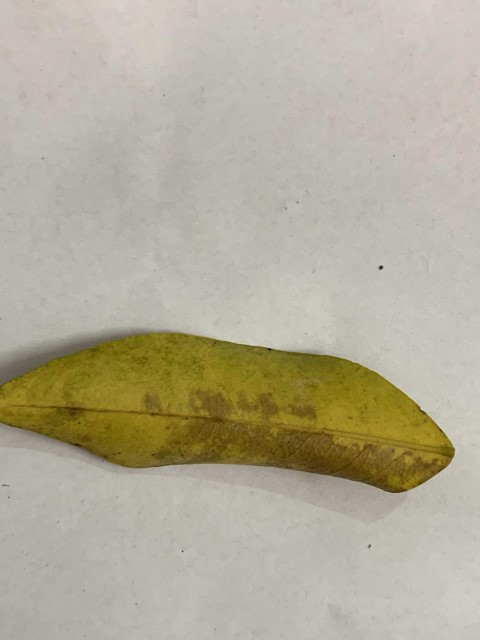

Supplement: Supplementary file 1 [file mmc1.zip › Sweetorange Sample Dataset/Converted Image/Yellow leaves/Con_Yellow Leaves35.jpeg]

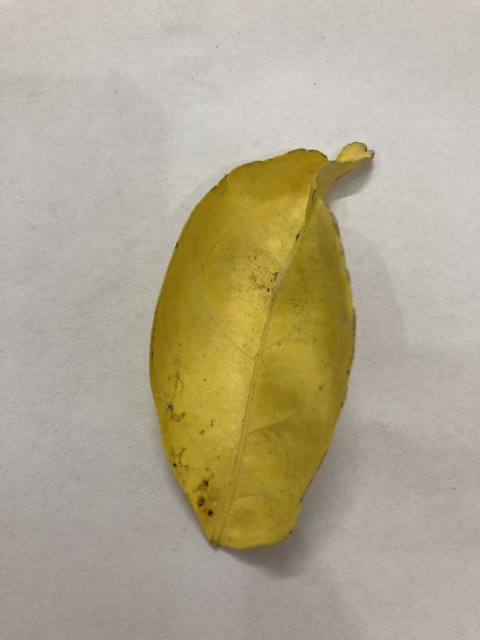

Supplement: Supplementary file 1 [file mmc1.zip › Sweetorange Sample Dataset/Converted Image/Yellow leaves/Con_Yellow Leaves42.jpeg]

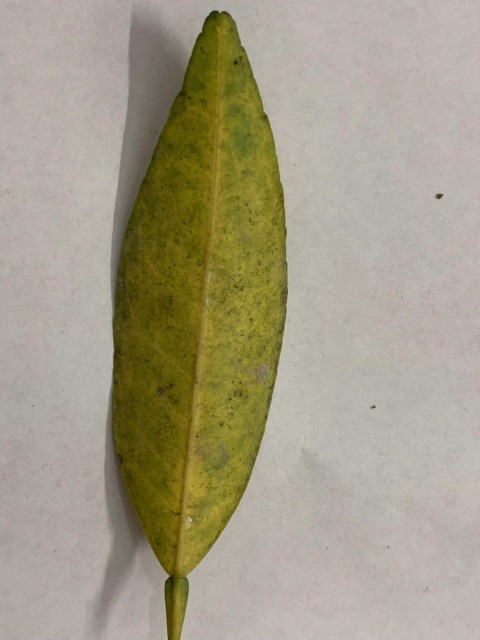

Supplement: Supplementary file 1 [file mmc1.zip › Sweetorange Sample Dataset/Converted Image/Yellow leaves/Con_Yellow Leaves14.jpeg]

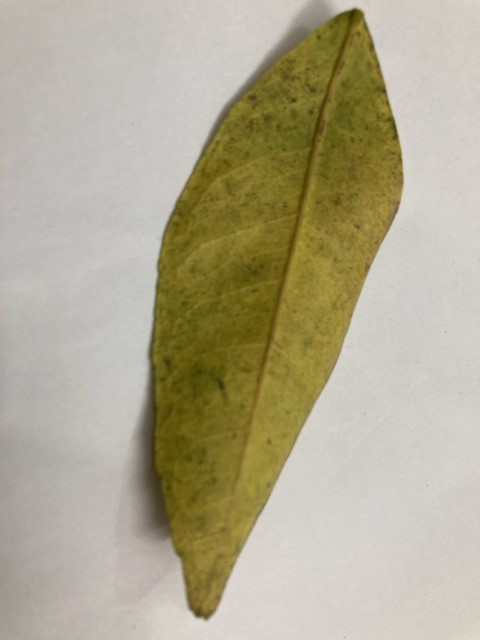

Supplement: Supplementary file 1 [file mmc1.zip › Sweetorange Sample Dataset/Converted Image/Yellow leaves/Con_Yellow Leaves301.jpeg]

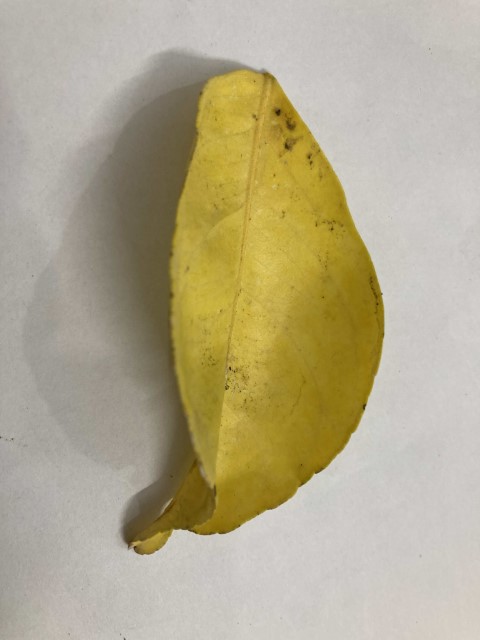

Supplement: Supplementary file 1 [file mmc1.zip › Sweetorange Sample Dataset/Converted Image/Yellow leaves/Con_Yellow Leaves63.jpeg]

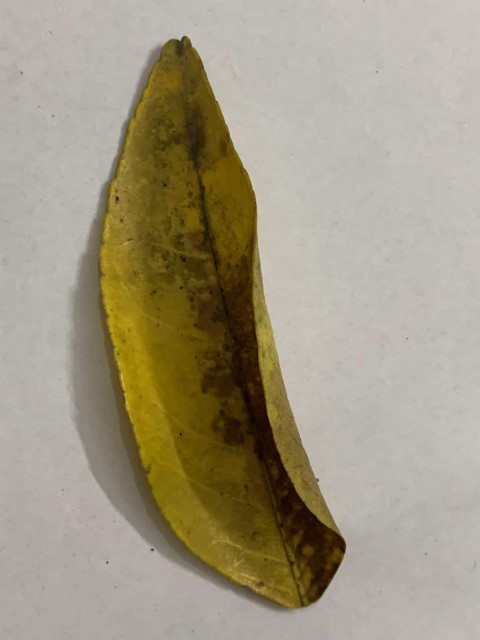

Supplement: Supplementary file 1 [file mmc1.zip › Sweetorange Sample Dataset/Converted Image/Yellow leaves/Con_Yellow Leaves21.jpeg]

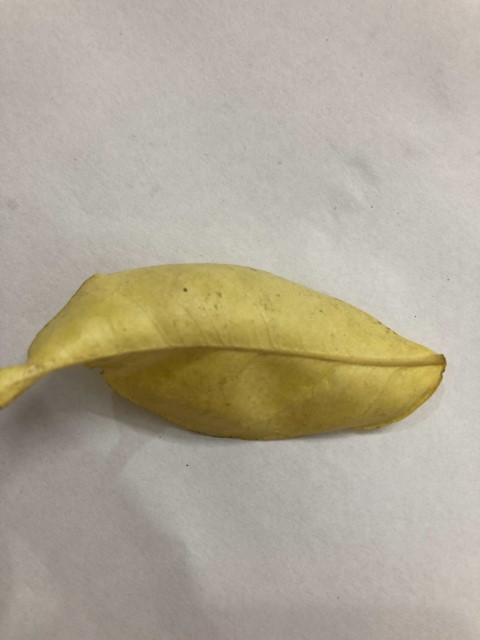

Supplement: Supplementary file 1 [file mmc1.zip › Sweetorange Sample Dataset/Converted Image/Yellow leaves/Con_Yellow Leaves56.jpeg]

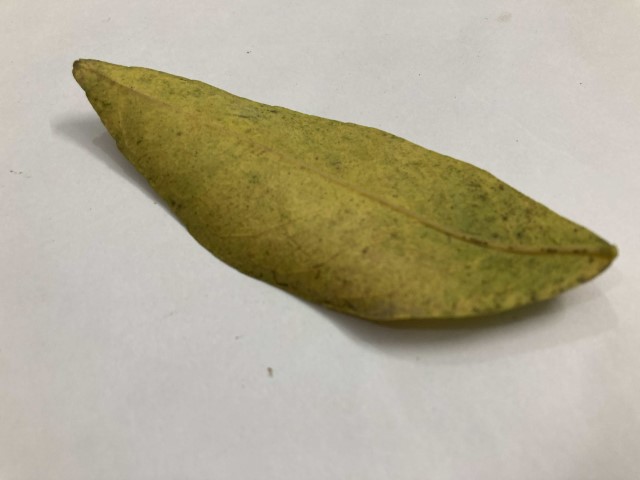

Supplement: Supplementary file 1 [file mmc1.zip › Sweetorange Sample Dataset/Converted Image/Yellow leaves/Con_Yellow Leaves308.jpeg]

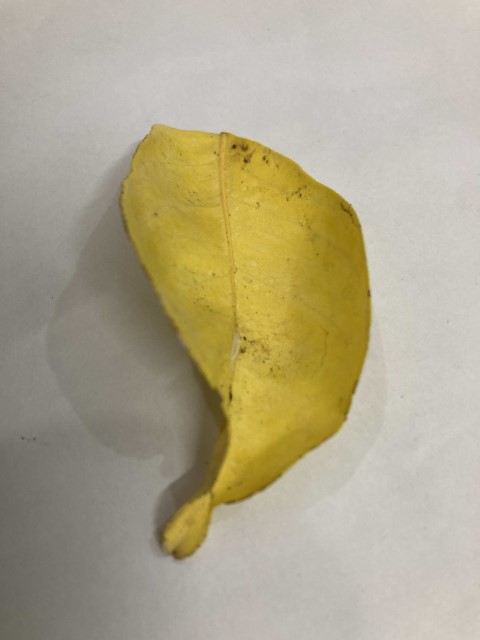

Supplement: Supplementary file 1 [file mmc1.zip › Sweetorange Sample Dataset/Converted Image/Yellow leaves/Con_Yellow Leaves70.jpeg]

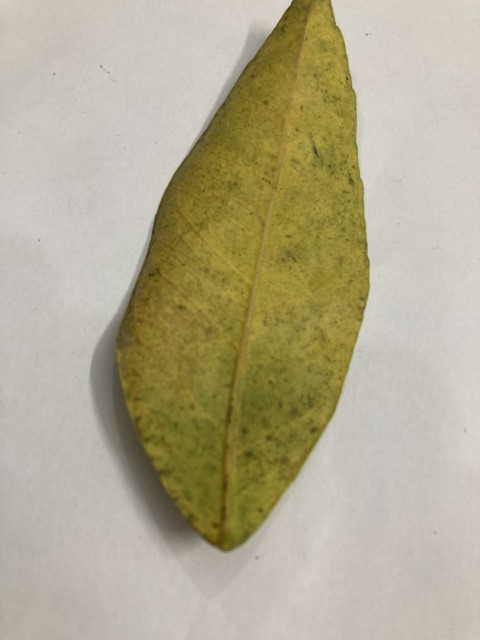

Supplement: Supplementary file 1 [file mmc1.zip › Sweetorange Sample Dataset/Converted Image/Yellow leaves/Con_Yellow Leaves294.jpeg]

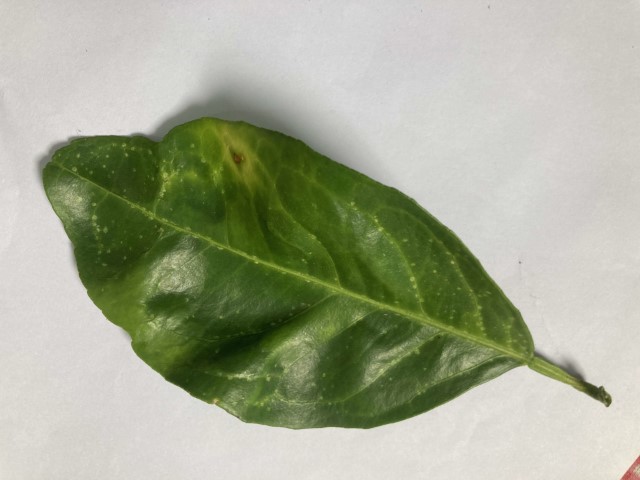

Supplement: Supplementary file 1 [file mmc1.zip › Sweetorange Sample Dataset/Converted Image/Citrus canker/Con_Citrus Canker91.jpeg]

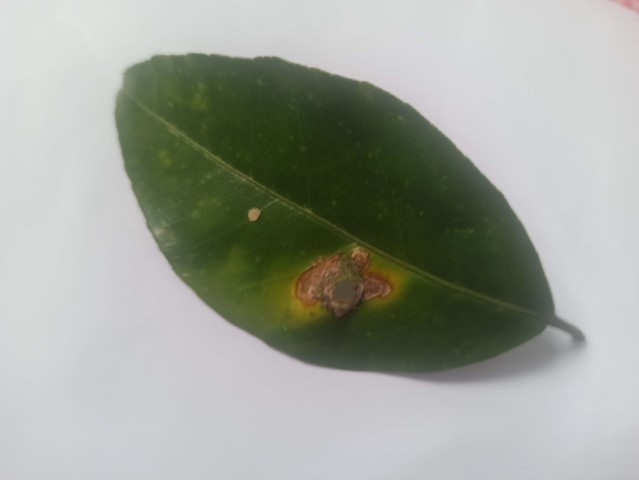

Supplement: Supplementary file 1 [file mmc1.zip › Sweetorange Sample Dataset/Converted Image/Citrus canker/Con_Citrus Canker588.jpeg]

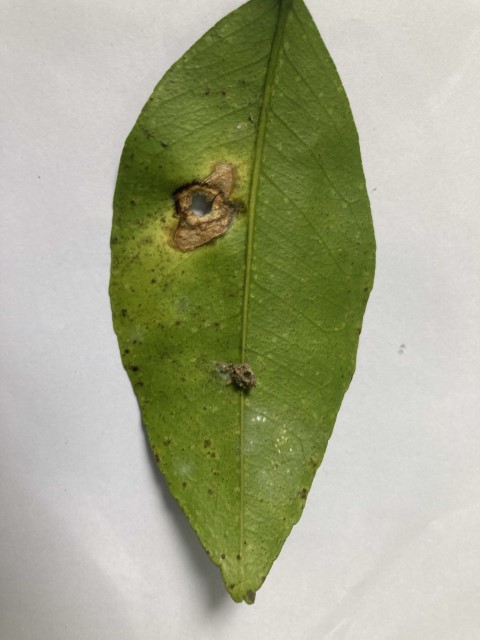

Supplement: Supplementary file 1 [file mmc1.zip › Sweetorange Sample Dataset/Converted Image/Citrus canker/Con_Citrus Canker49.jpeg]

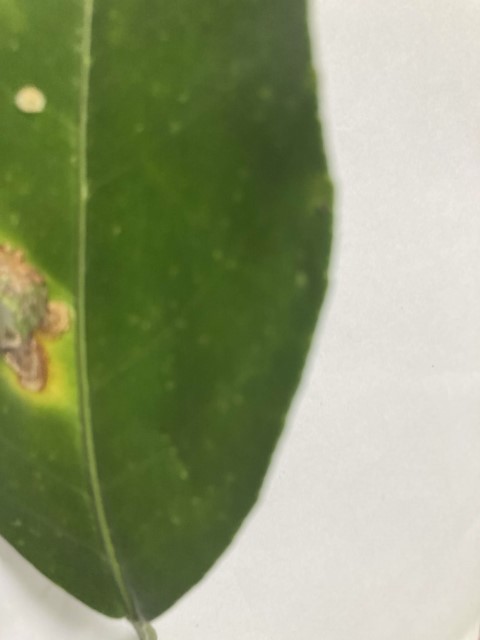

Supplement: Supplementary file 1 [file mmc1.zip › Sweetorange Sample Dataset/Converted Image/Citrus canker/Con_Citrus Canker28.jpeg]

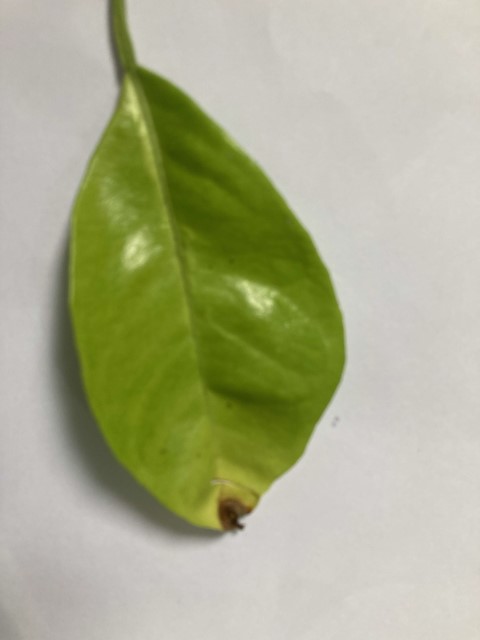

Supplement: Supplementary file 1 [file mmc1.zip › Sweetorange Sample Dataset/Converted Image/Citrus canker/Con_Citrus Canker35.jpeg]

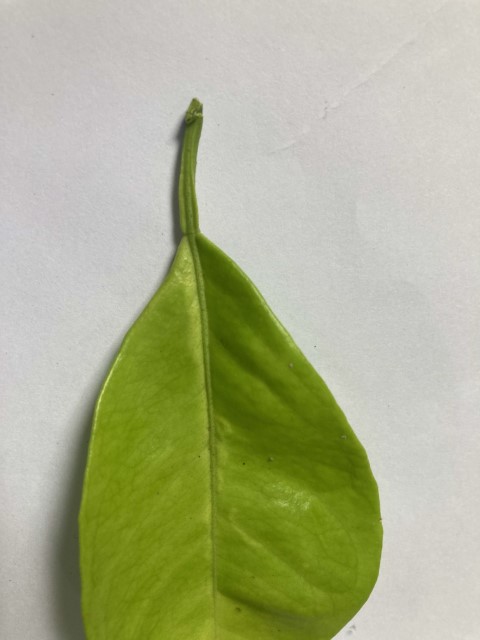

Supplement: Supplementary file 1 [file mmc1.zip › Sweetorange Sample Dataset/Converted Image/Citrus canker/Con_Citrus Canker42.jpeg]

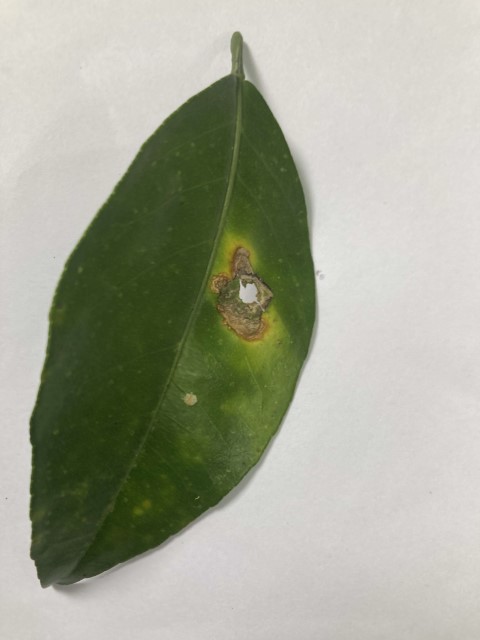

Supplement: Supplementary file 1 [file mmc1.zip › Sweetorange Sample Dataset/Converted Image/Citrus canker/Con_Citrus Canker14.jpeg]

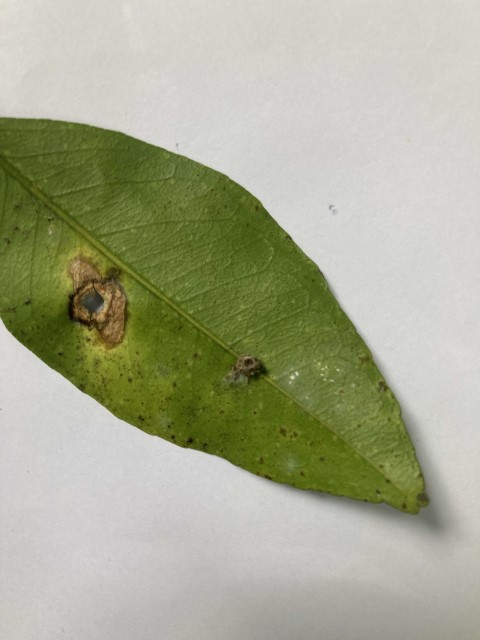

Supplement: Supplementary file 1 [file mmc1.zip › Sweetorange Sample Dataset/Converted Image/Citrus canker/Con_Citrus Canker63.jpeg]

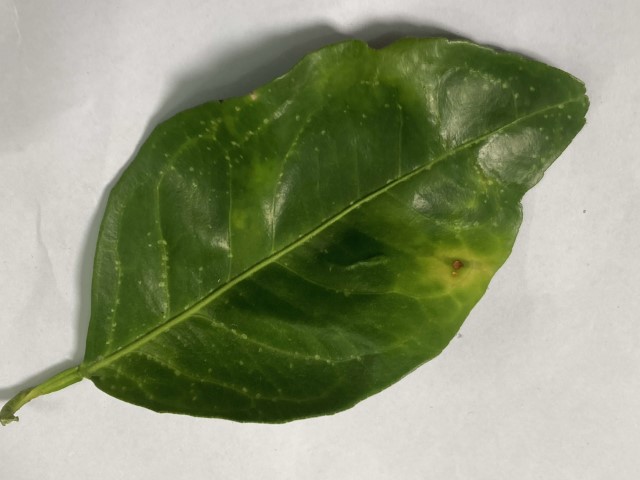

Supplement: Supplementary file 1 [file mmc1.zip › Sweetorange Sample Dataset/Converted Image/Citrus canker/Con_Citrus Canker126.jpeg]

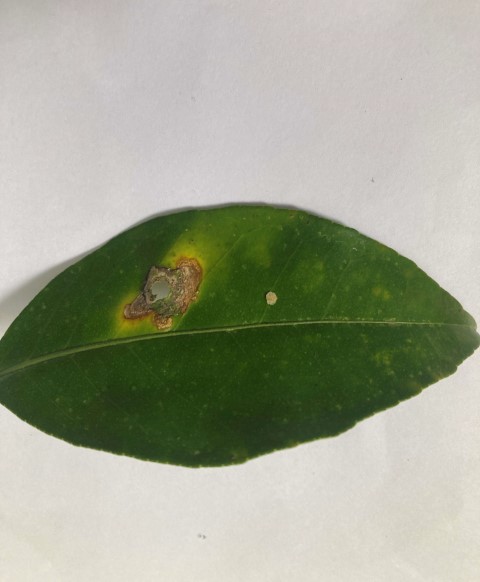

Supplement: Supplementary file 1 [file mmc1.zip › Sweetorange Sample Dataset/Converted Image/Citrus canker/Con_Citrus Canker7.jpeg]

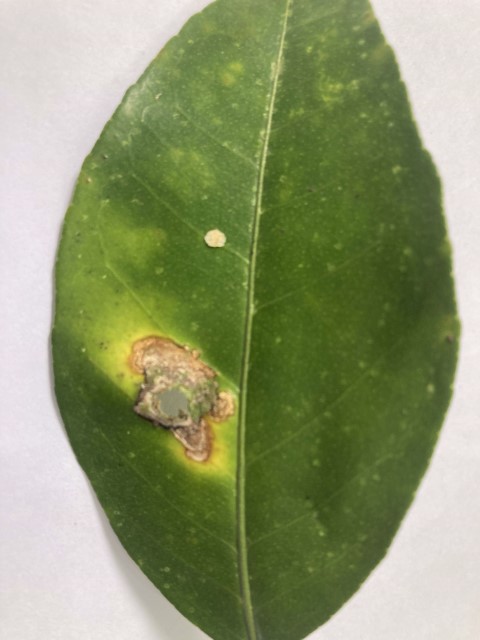

Supplement: Supplementary file 1 [file mmc1.zip › Sweetorange Sample Dataset/Converted Image/Citrus canker/Con_Citrus Canker21.jpeg]

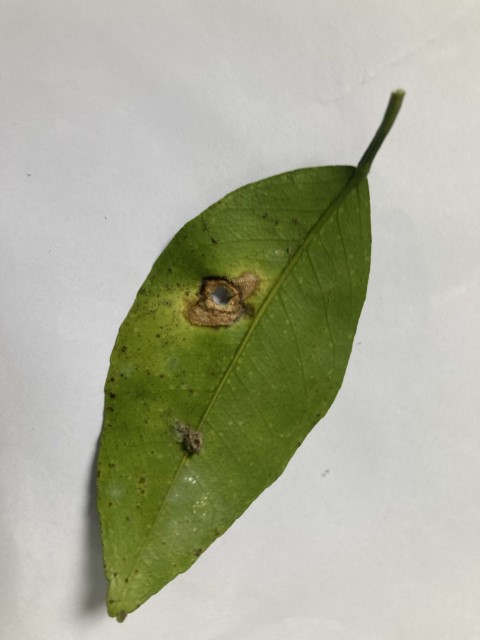

Supplement: Supplementary file 1 [file mmc1.zip › Sweetorange Sample Dataset/Converted Image/Citrus canker/Con_Citrus Canker56.jpeg]

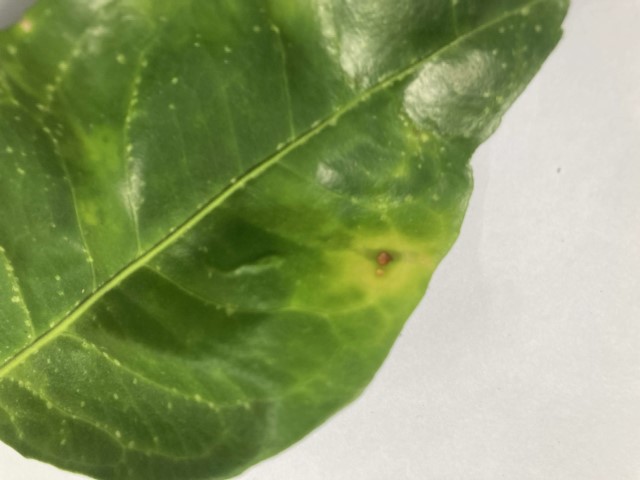

Supplement: Supplementary file 1 [file mmc1.zip › Sweetorange Sample Dataset/Converted Image/Citrus canker/Con_Citrus Canker105.jpeg]

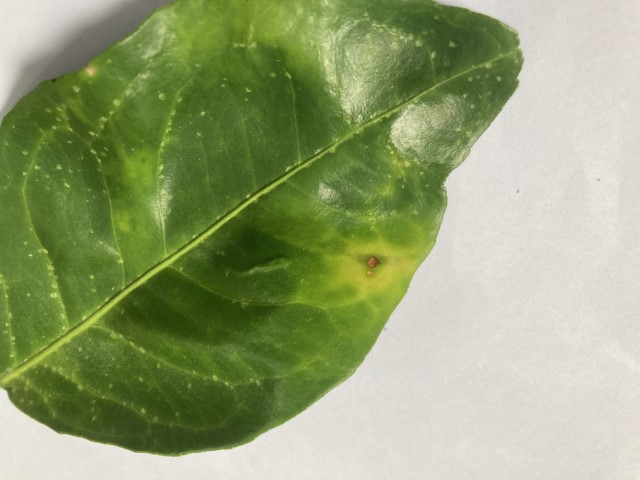

Supplement: Supplementary file 1 [file mmc1.zip › Sweetorange Sample Dataset/Converted Image/Citrus canker/Con_Citrus Canker112.jpeg]

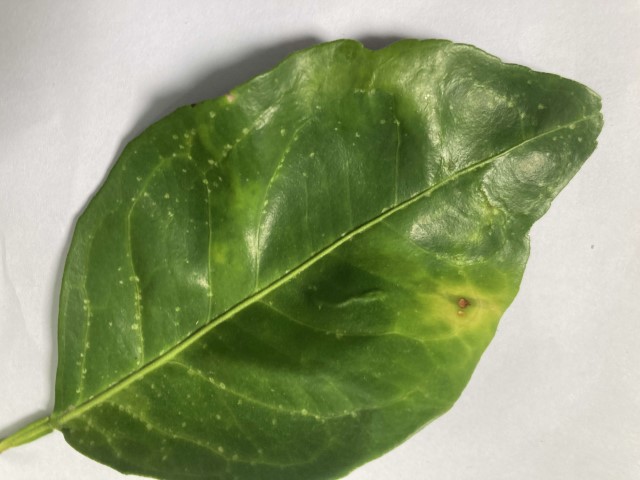

Supplement: Supplementary file 1 [file mmc1.zip › Sweetorange Sample Dataset/Converted Image/Citrus canker/Con_Citrus Canker98.jpeg]

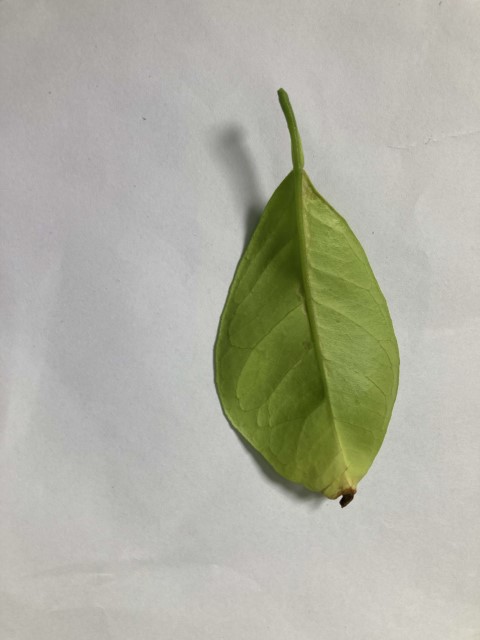

Supplement: Supplementary file 1 [file mmc1.zip › Sweetorange Sample Dataset/Converted Image/Citrus canker/Con_Citrus Canker77.jpeg]

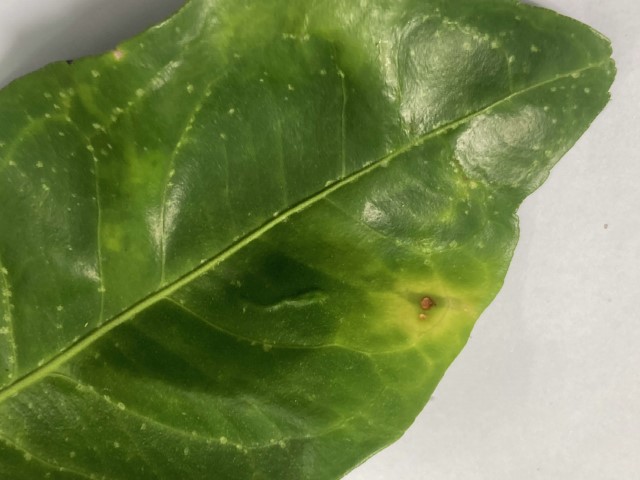

Supplement: Supplementary file 1 [file mmc1.zip › Sweetorange Sample Dataset/Converted Image/Citrus canker/Con_Citrus Canker119.jpeg]

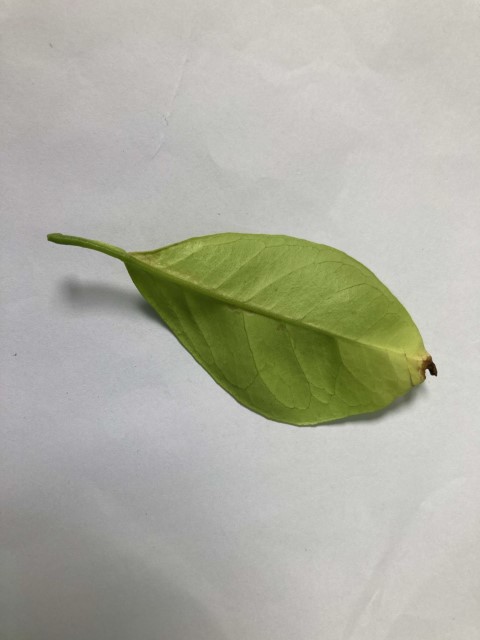

Supplement: Supplementary file 1 [file mmc1.zip › Sweetorange Sample Dataset/Converted Image/Citrus canker/Con_Citrus Canker70.jpeg]

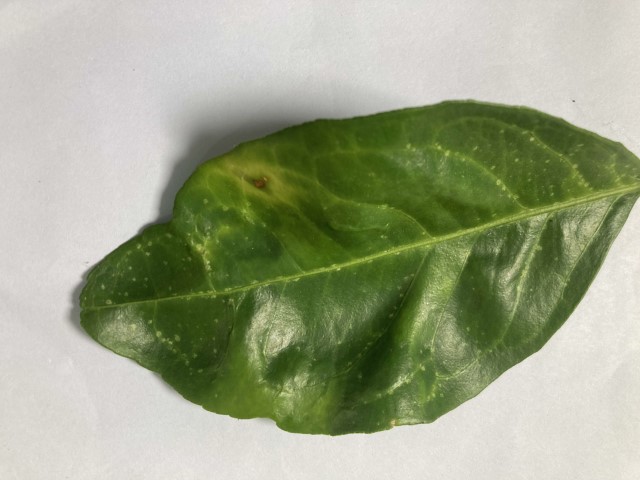

Supplement: Supplementary file 1 [file mmc1.zip › Sweetorange Sample Dataset/Converted Image/Citrus canker/Con_Citrus Canker84.jpeg]

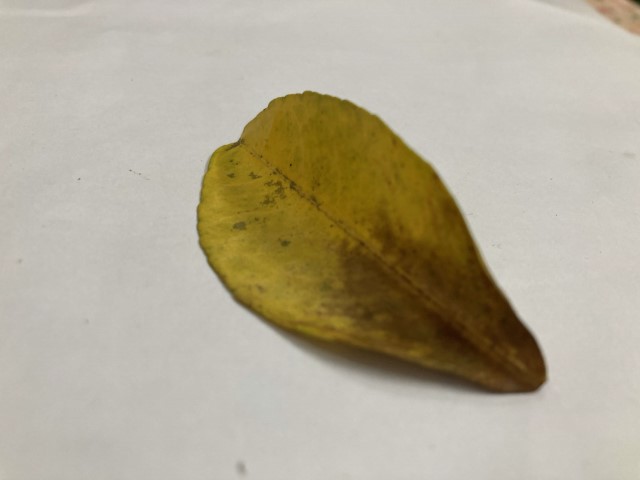

Supplement: Supplementary file 1 [file mmc1.zip › Sweetorange Sample Dataset/Converted Image/Die back/Con_Die Back63.jpeg]
