# Supplementary figures and images for: Multi-format open-source sweet orange leaf dataset for disease detection, classification, and analysis (part 1 of 2)
Source: Data Brief. 2024 Jul 6;55:110713. doi: 10.1016/j.dib.2024.110713 (PMC11295629; doi:10.1016/j.dib.2024.110713)

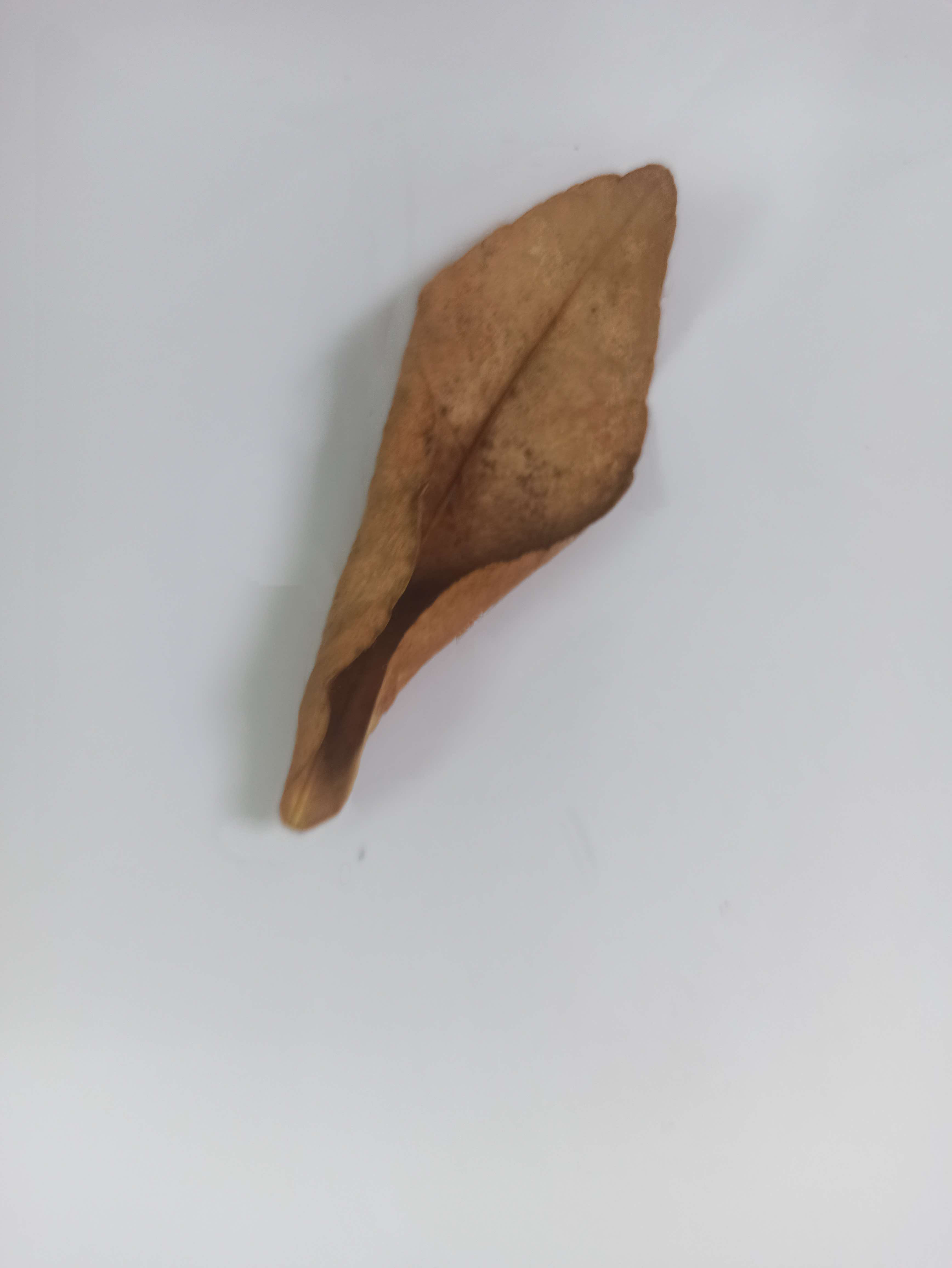

Supplement: Supplementary file 1 [file mmc1.zip › Sweetorange Sample Dataset/Annotation/Die_back (5).jpg]

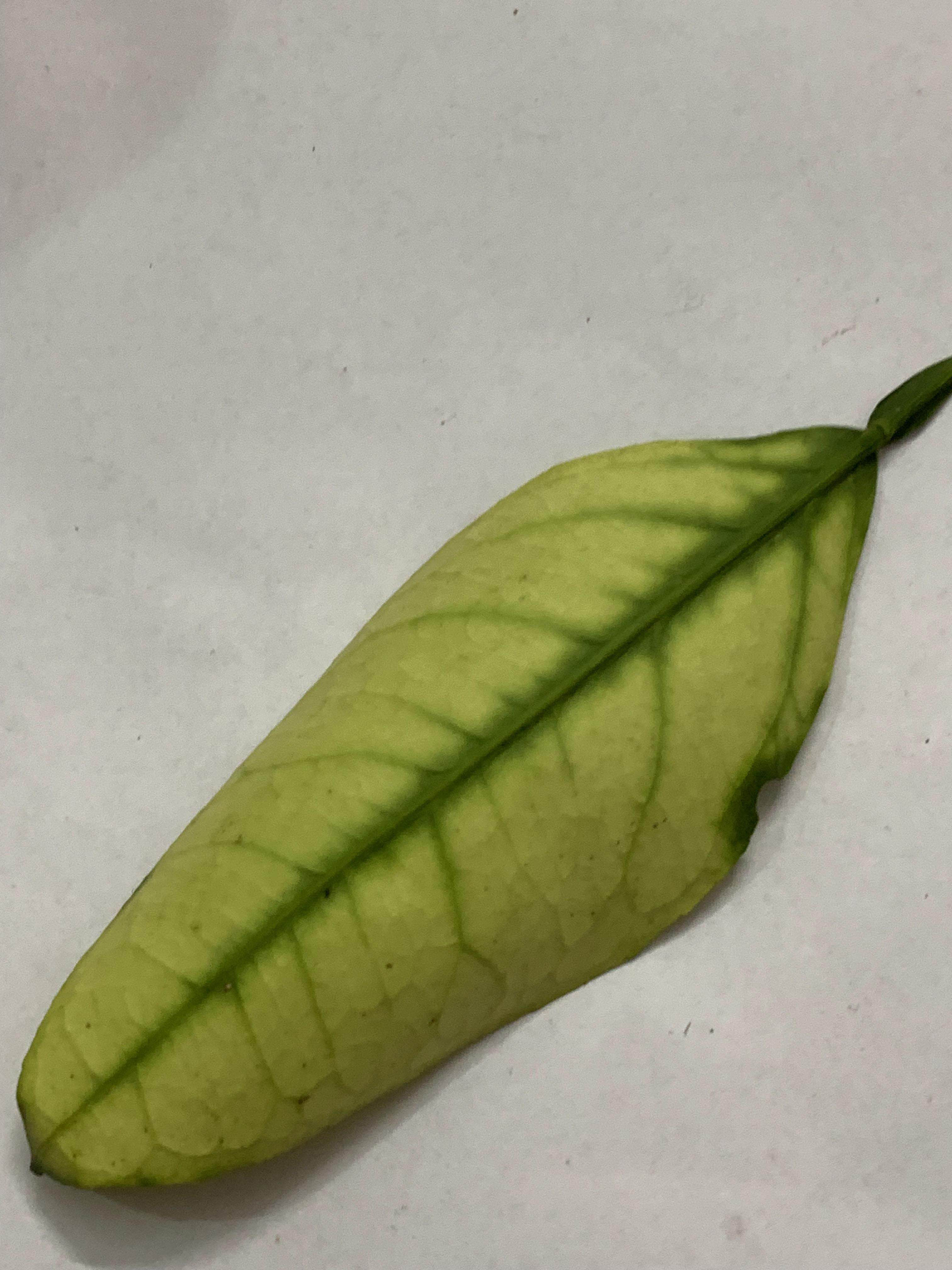

Supplement: Supplementary file 1 [file mmc1.zip › Sweetorange Sample Dataset/Annotation/Yellow_dragon (5).jpg]

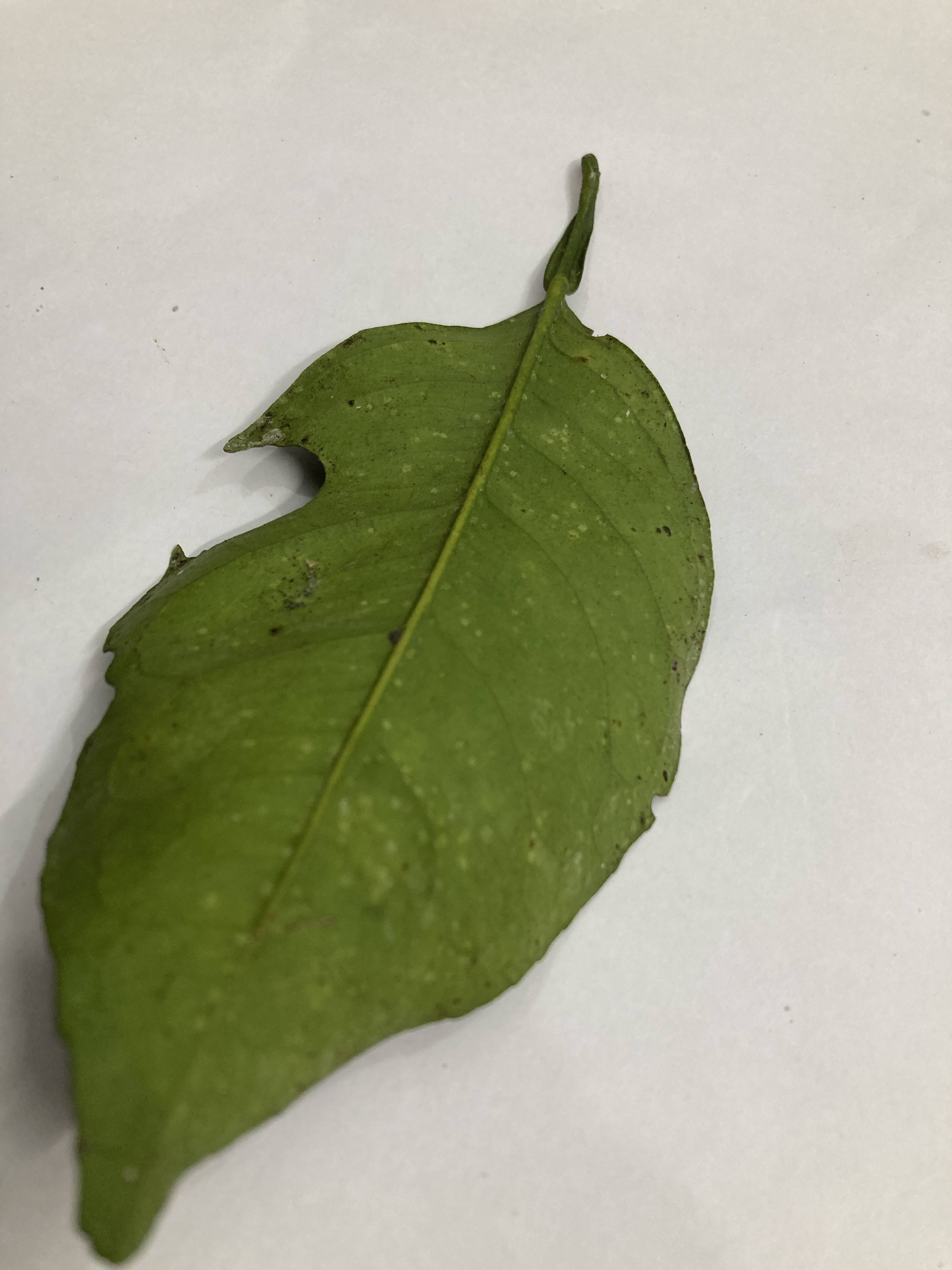

Supplement: Supplementary file 1 [file mmc1.zip › Sweetorange Sample Dataset/Annotation/Powdery_mildew (4).jpg]

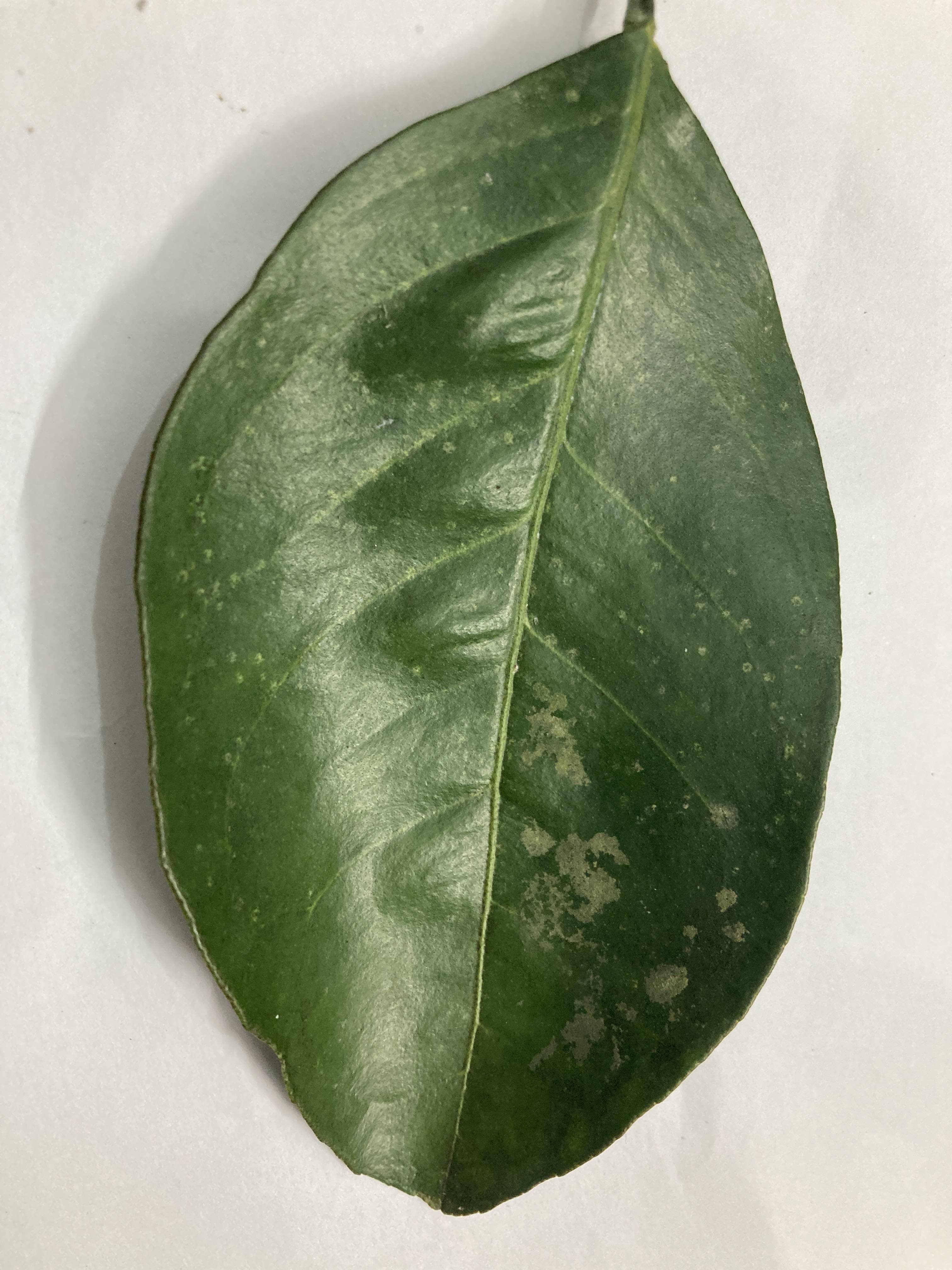

Supplement: Supplementary file 1 [file mmc1.zip › Sweetorange Sample Dataset/Annotation/Powdery_mildew (5).jpg]

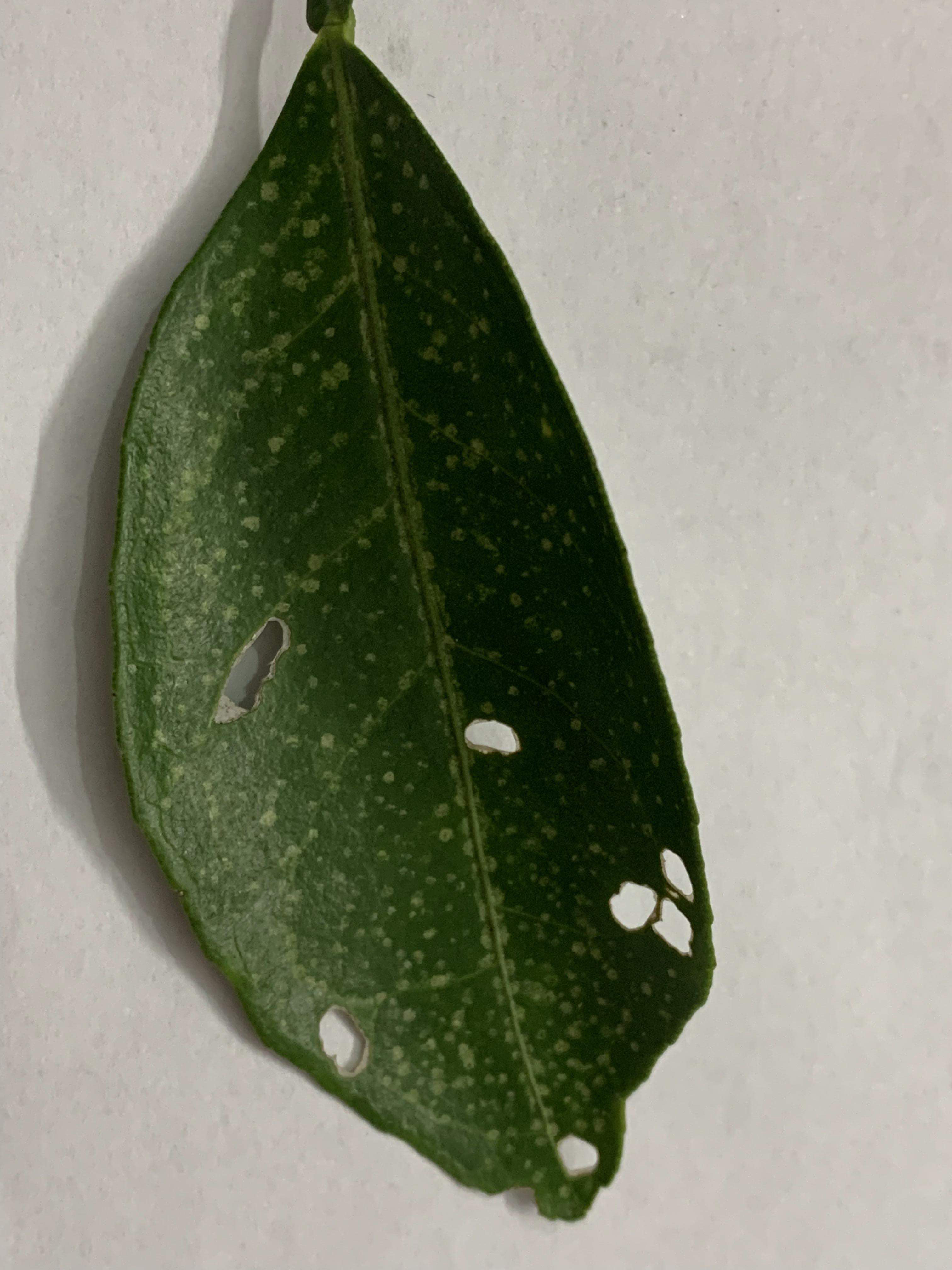

Supplement: Supplementary file 1 [file mmc1.zip › Sweetorange Sample Dataset/Annotation/Shot_hole (1).jpg]

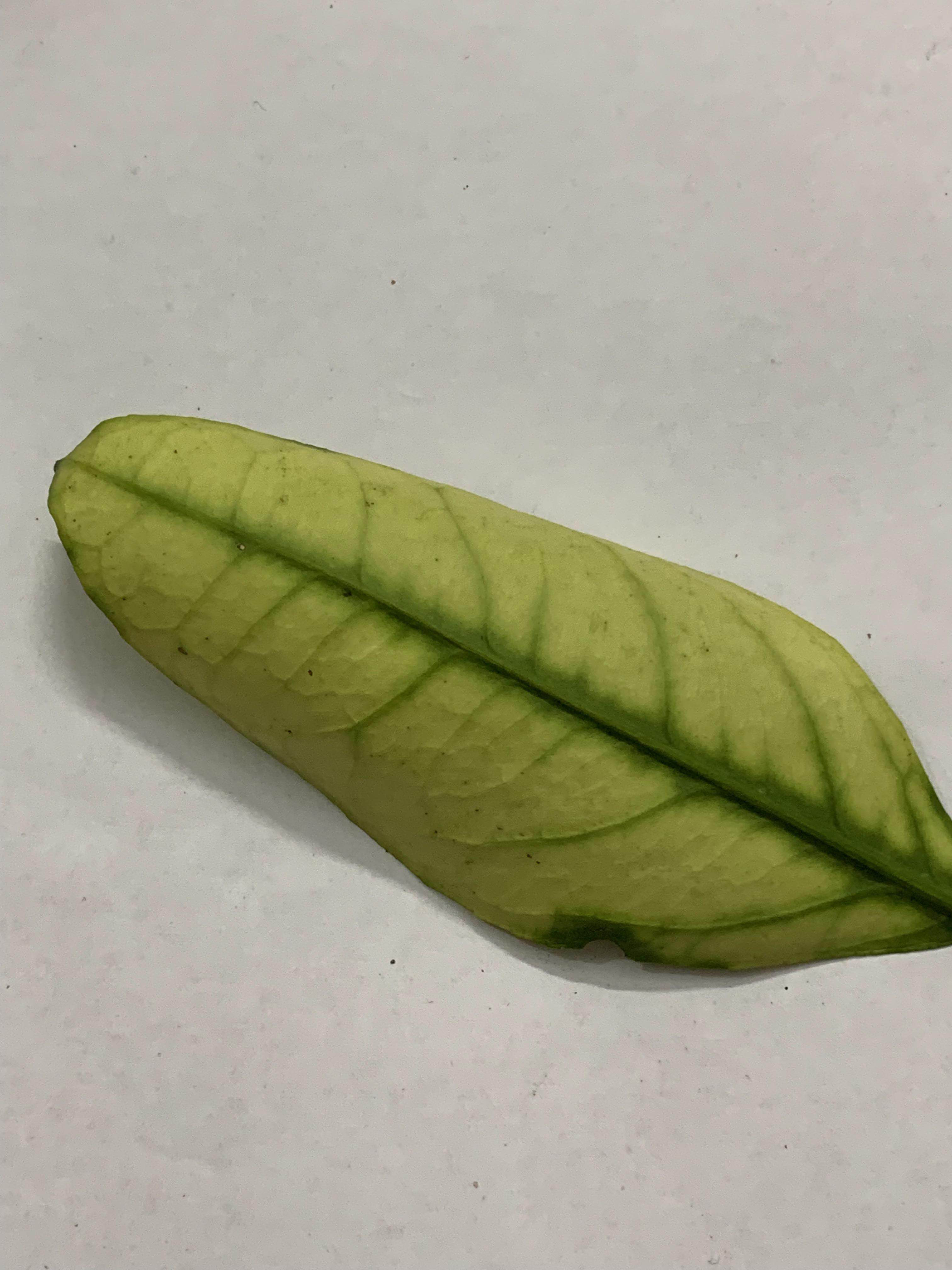

Supplement: Supplementary file 1 [file mmc1.zip › Sweetorange Sample Dataset/Annotation/Yellow_dragon (4).jpg]

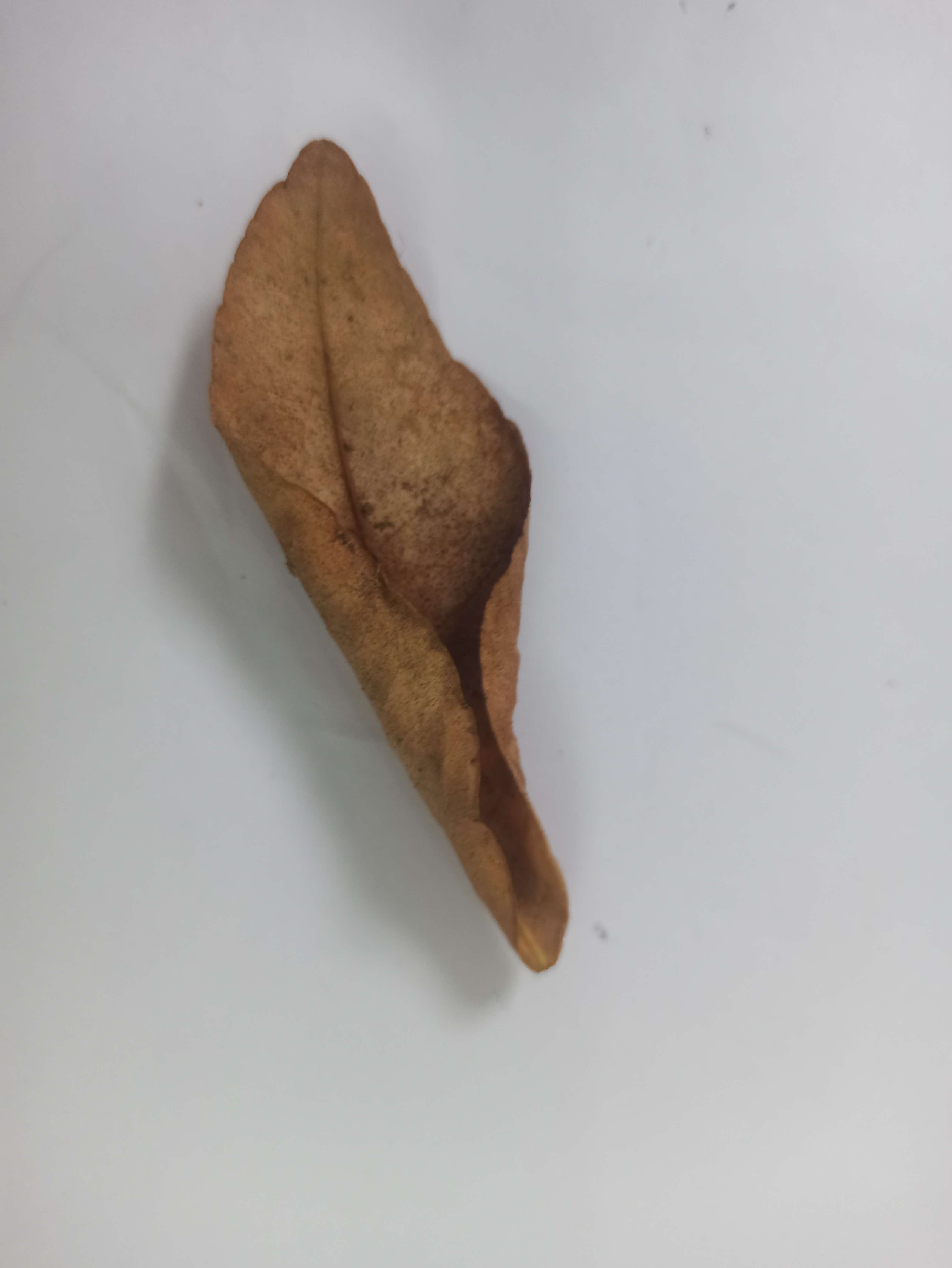

Supplement: Supplementary file 1 [file mmc1.zip › Sweetorange Sample Dataset/Annotation/Die_back (4).jpg]

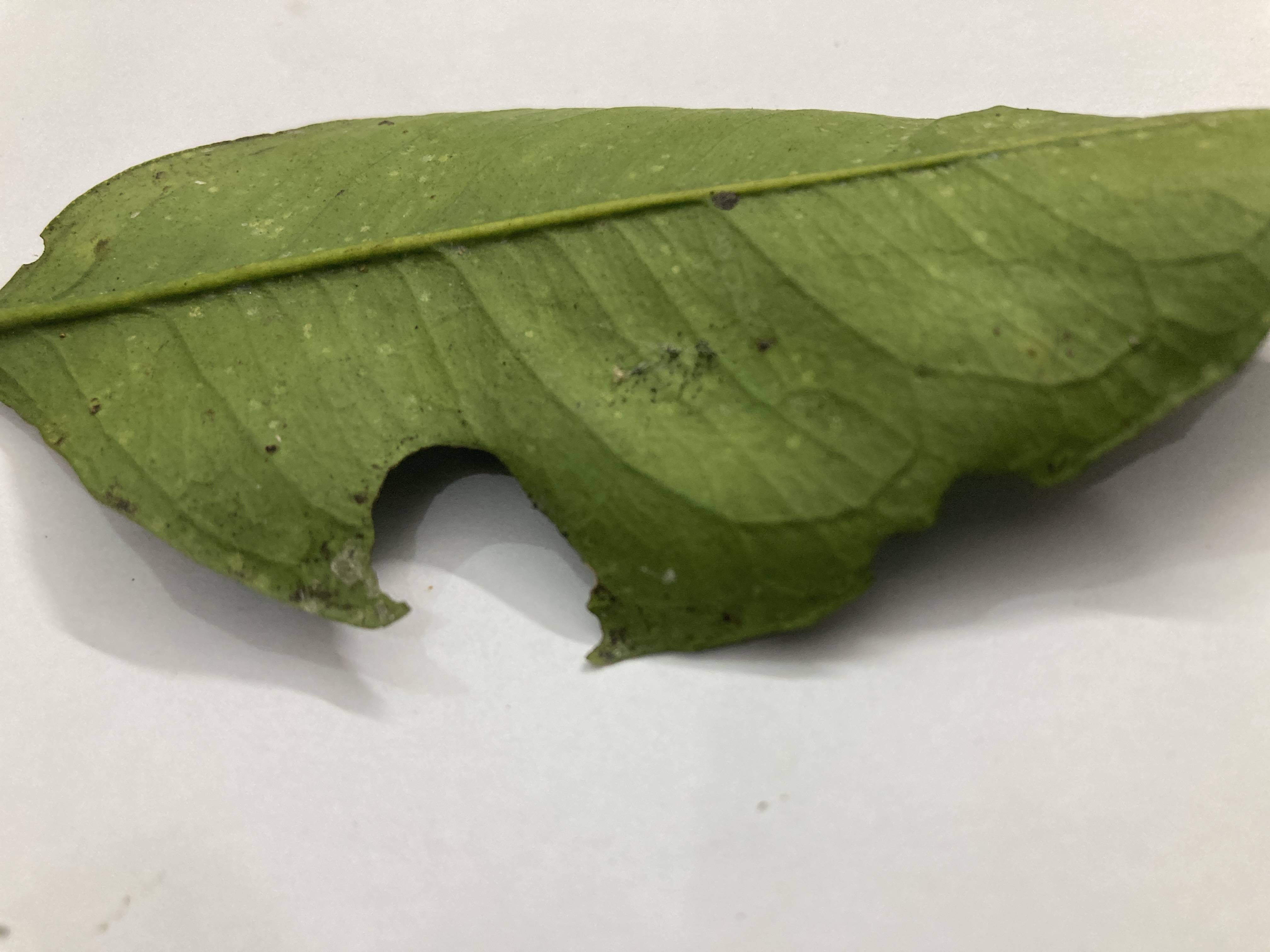

Supplement: Supplementary file 1 [file mmc1.zip › Sweetorange Sample Dataset/Annotation/Powdery_mildew (2).jpg]

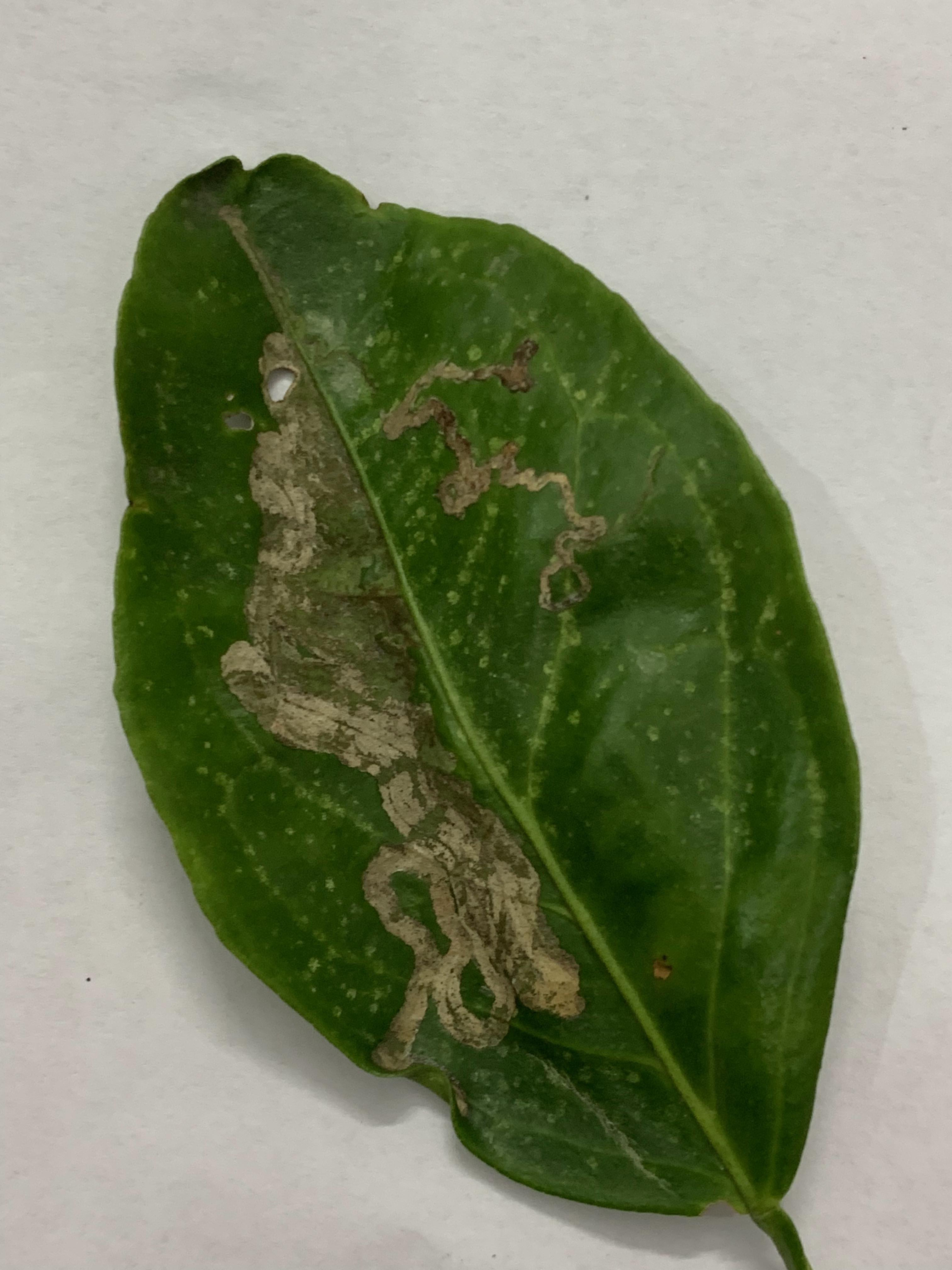

Supplement: Supplementary file 1 [file mmc1.zip › Sweetorange Sample Dataset/Annotation/Foliage_damaged (1).jpg]

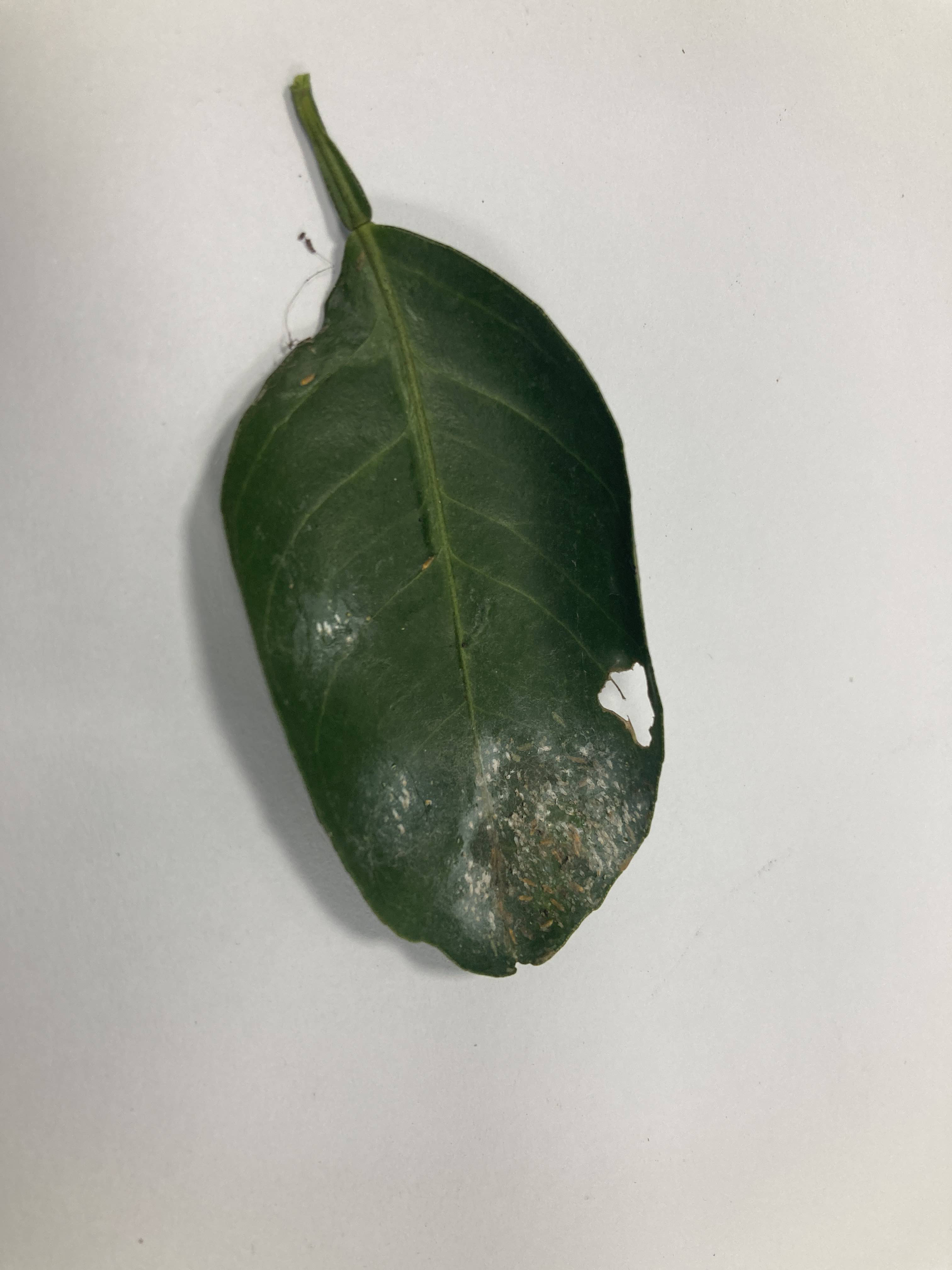

Supplement: Supplementary file 1 [file mmc1.zip › Sweetorange Sample Dataset/Annotation/Citrus_mealybugs (1).jpg]

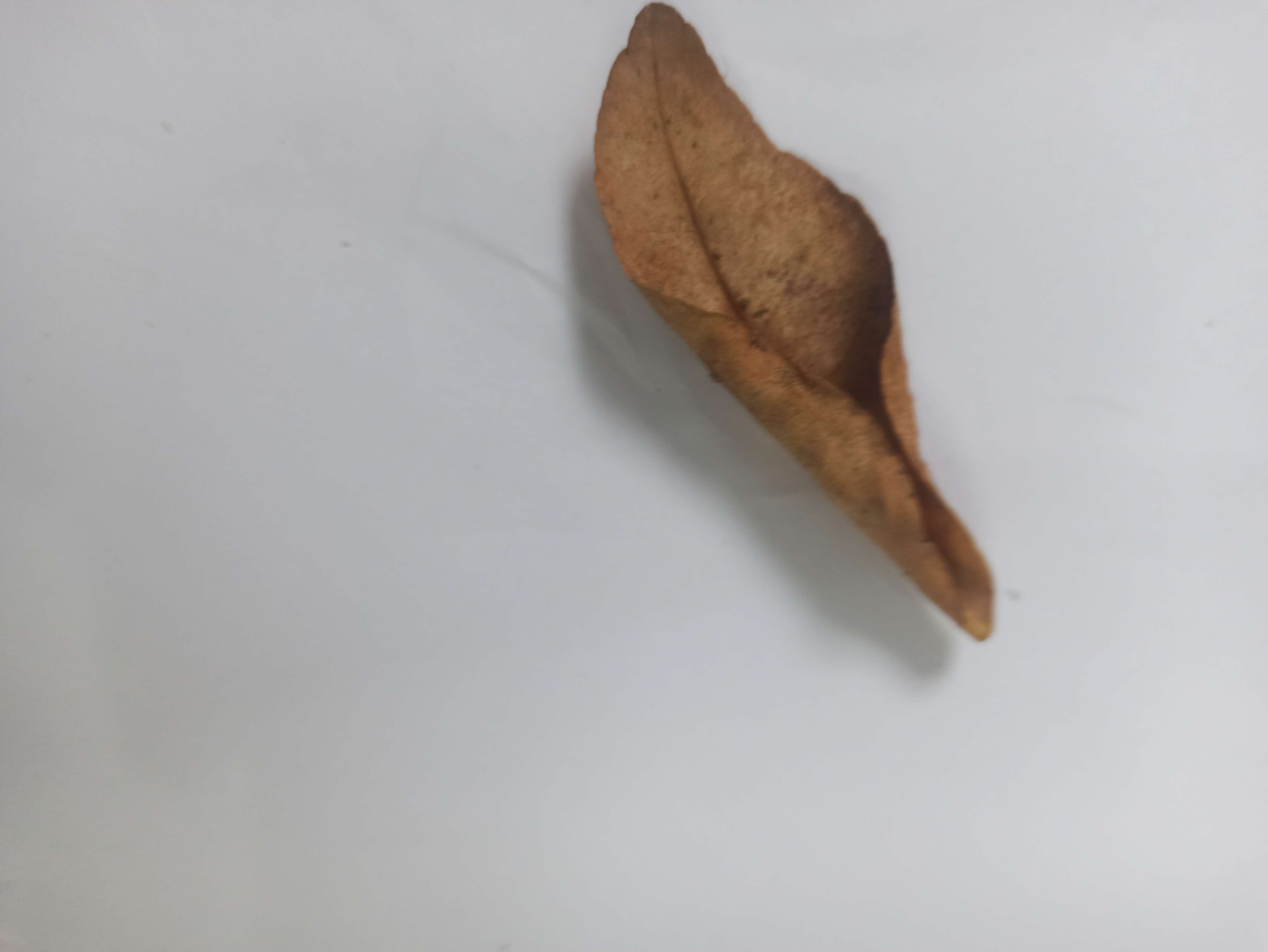

Supplement: Supplementary file 1 [file mmc1.zip › Sweetorange Sample Dataset/Annotation/Die_back (3).jpg]

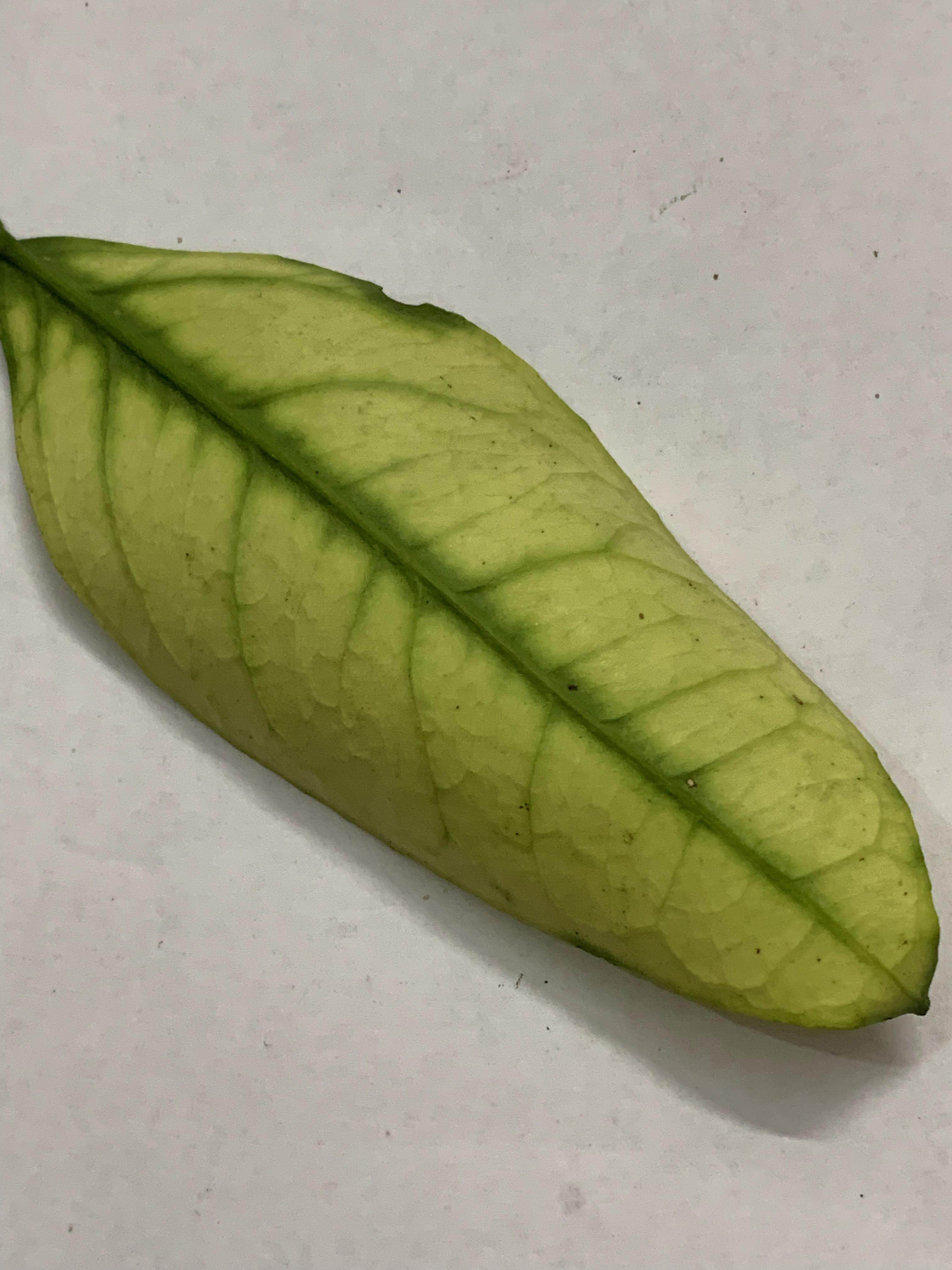

Supplement: Supplementary file 1 [file mmc1.zip › Sweetorange Sample Dataset/Annotation/Yellow_dragon (3).jpg]

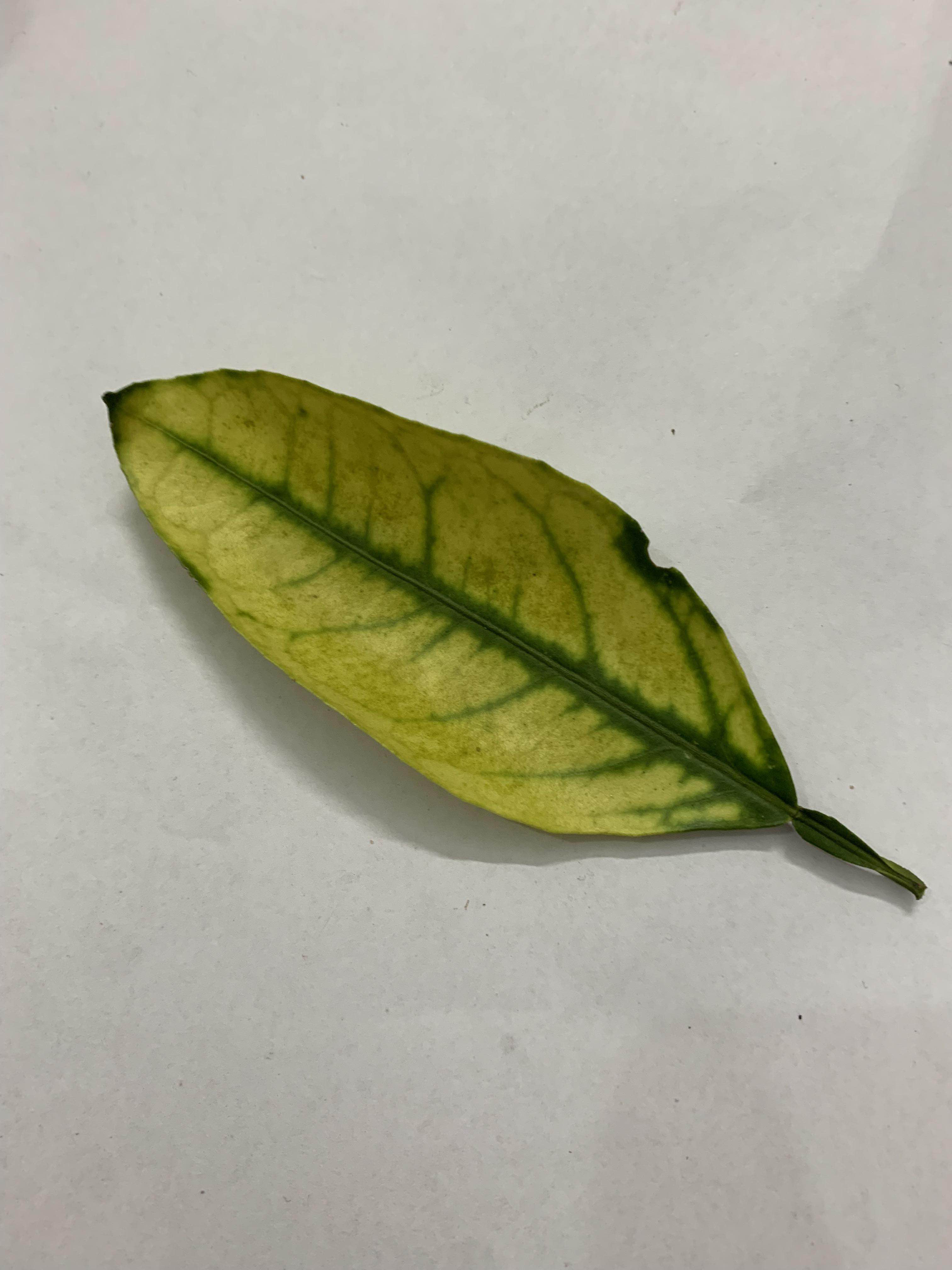

Supplement: Supplementary file 1 [file mmc1.zip › Sweetorange Sample Dataset/Annotation/Yellow_dragon (2).jpg]

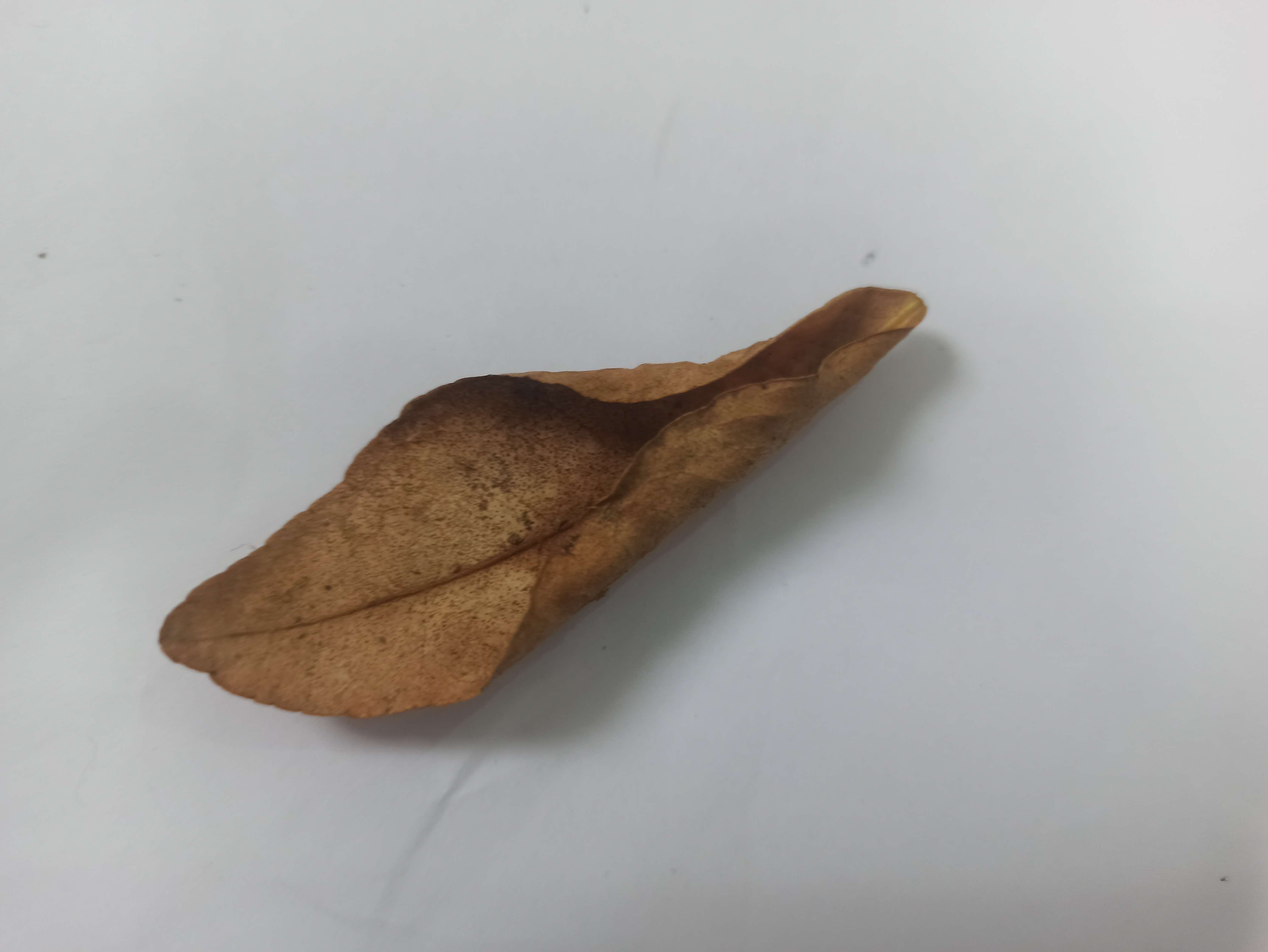

Supplement: Supplementary file 1 [file mmc1.zip › Sweetorange Sample Dataset/Annotation/Die_back (2).jpg]

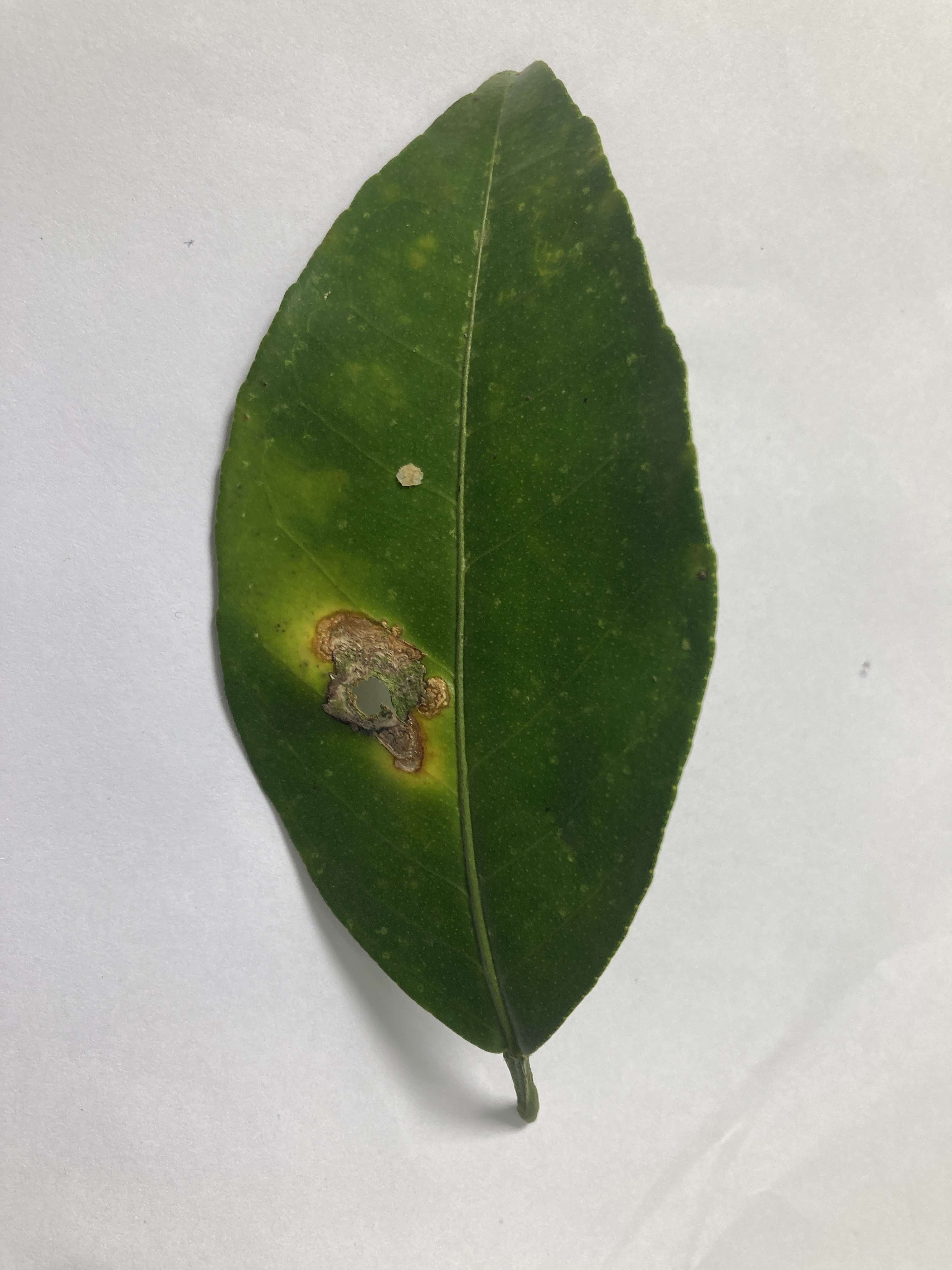

Supplement: Supplementary file 1 [file mmc1.zip › Sweetorange Sample Dataset/Annotation/Citrus_canker (1).jpg]

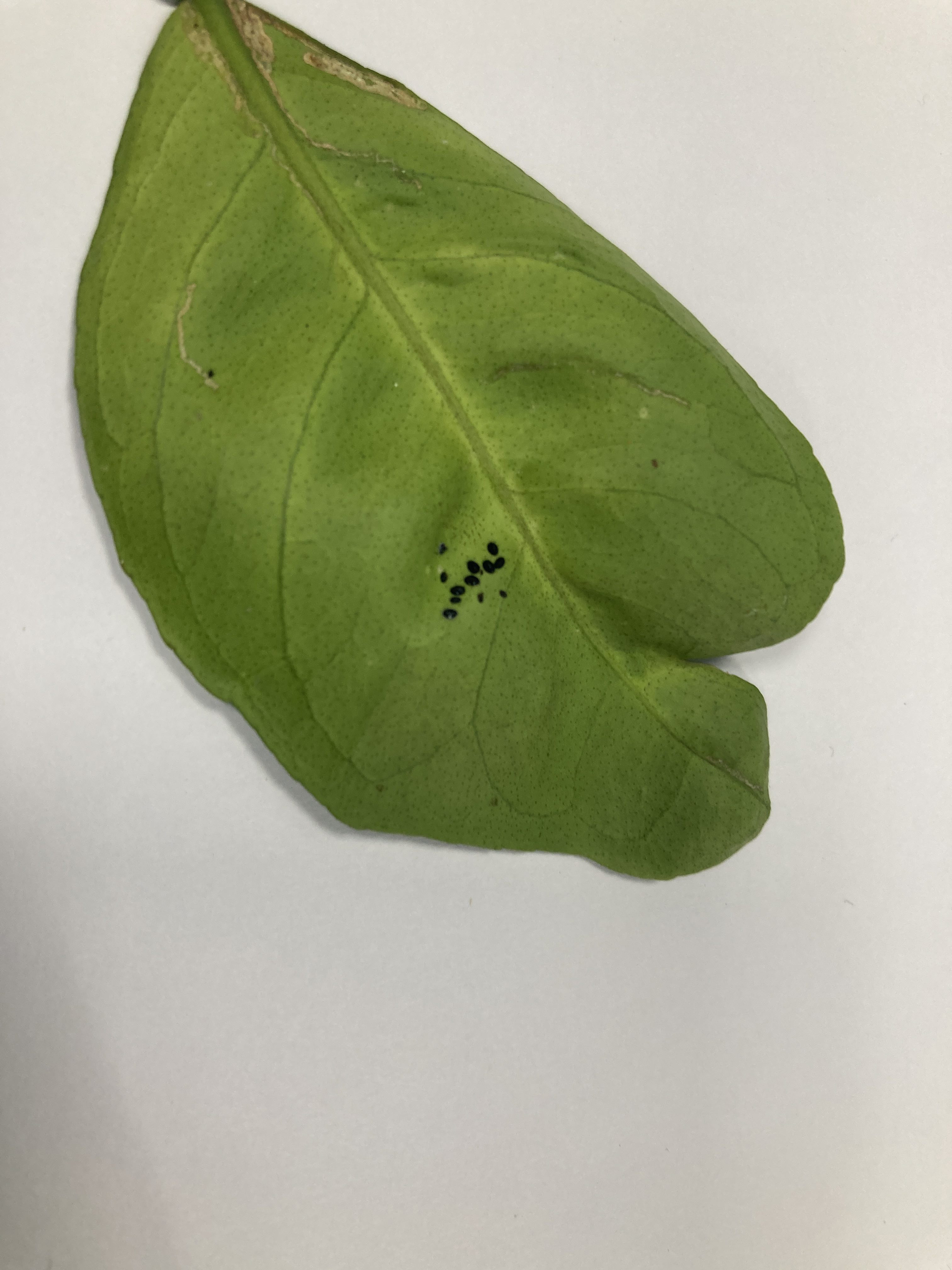

Supplement: Supplementary file 1 [file mmc1.zip › Sweetorange Sample Dataset/Annotation/Spiny_whitefly (1).jpg]

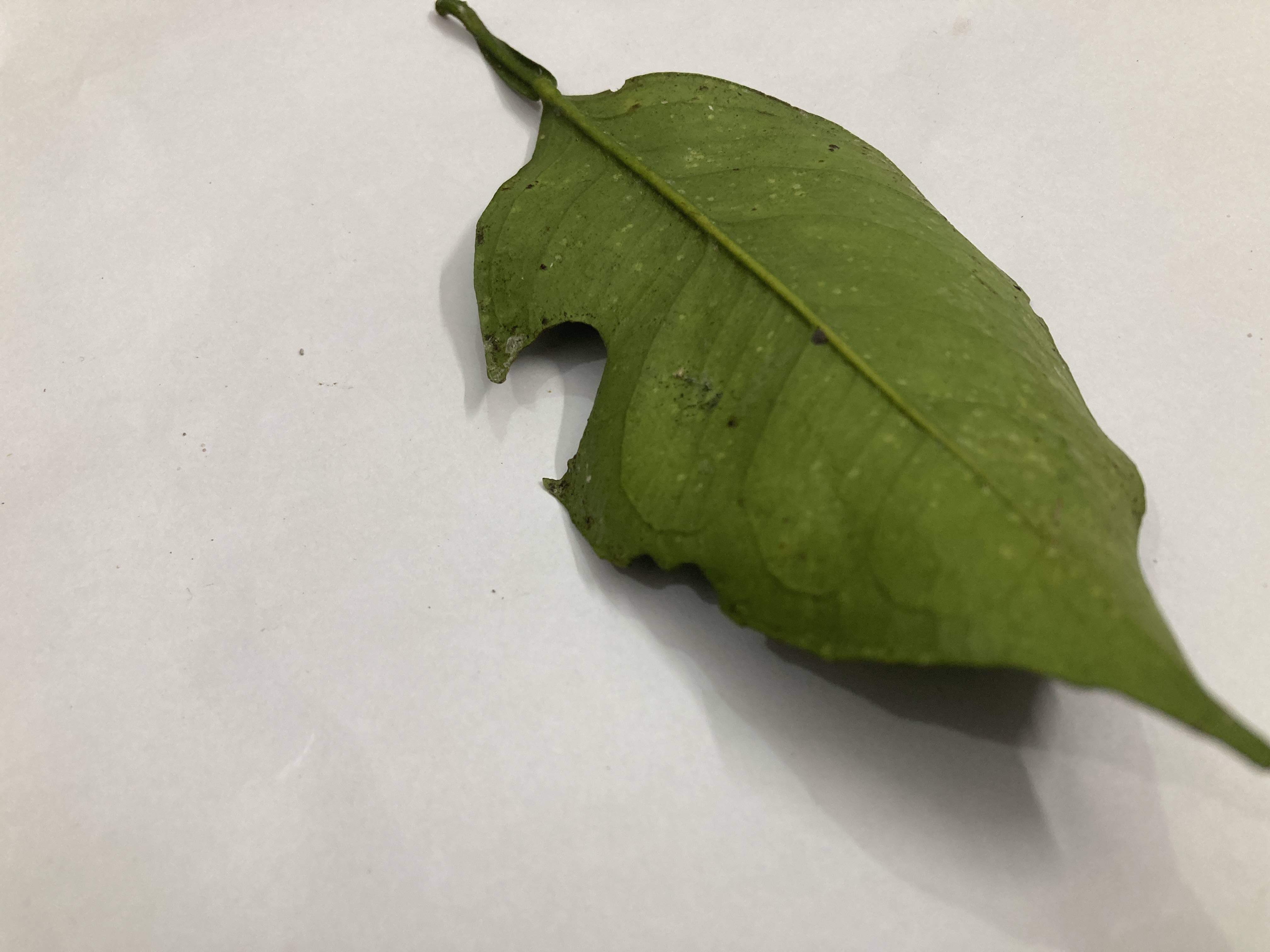

Supplement: Supplementary file 1 [file mmc1.zip › Sweetorange Sample Dataset/Annotation/Powdery_mildew (3).jpg]

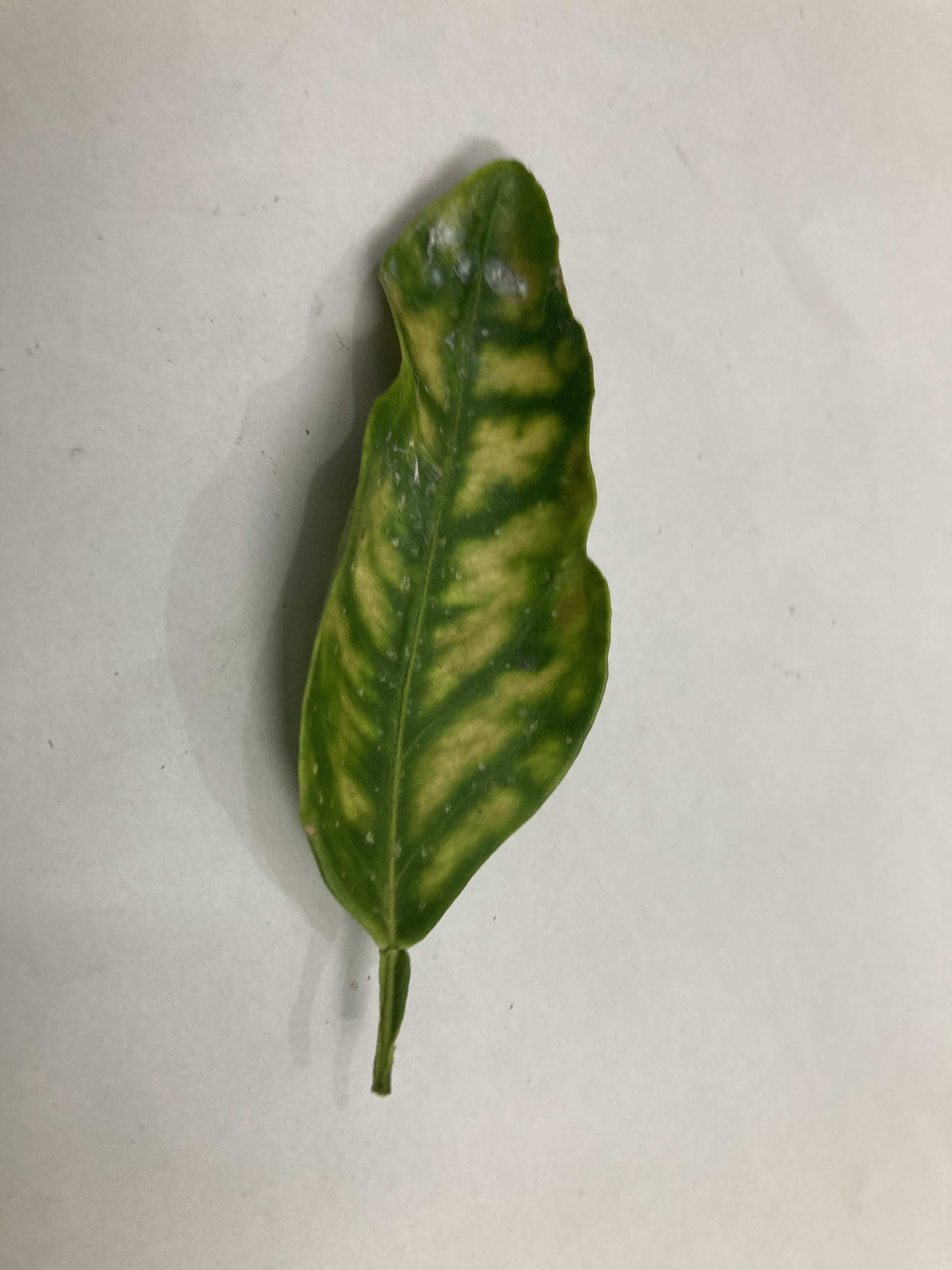

Supplement: Supplementary file 1 [file mmc1.zip › Sweetorange Sample Dataset/Annotation/Citrus_greening (1).jpg]

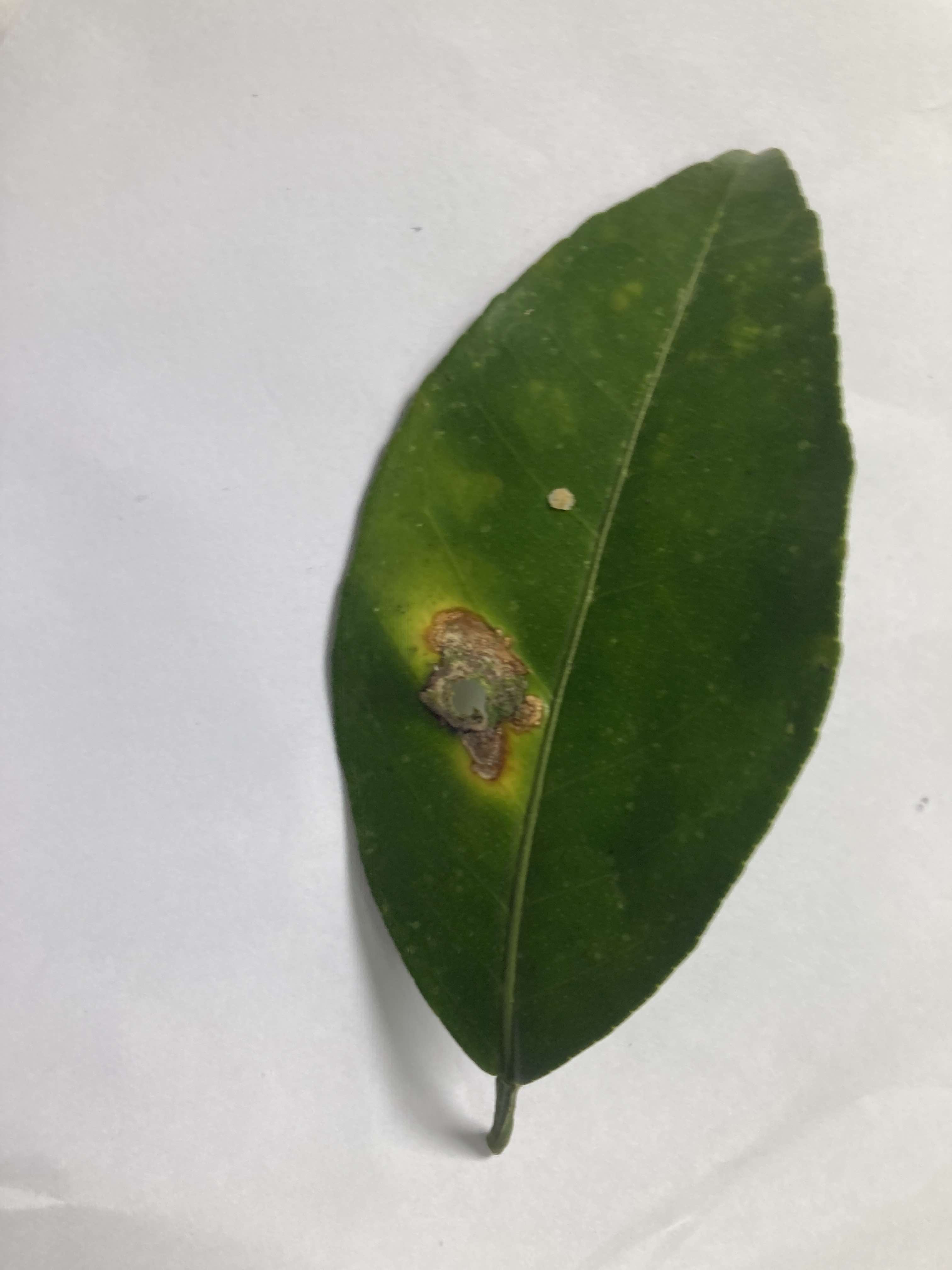

Supplement: Supplementary file 1 [file mmc1.zip › Sweetorange Sample Dataset/Annotation/Citrus_canker (2).jpg]

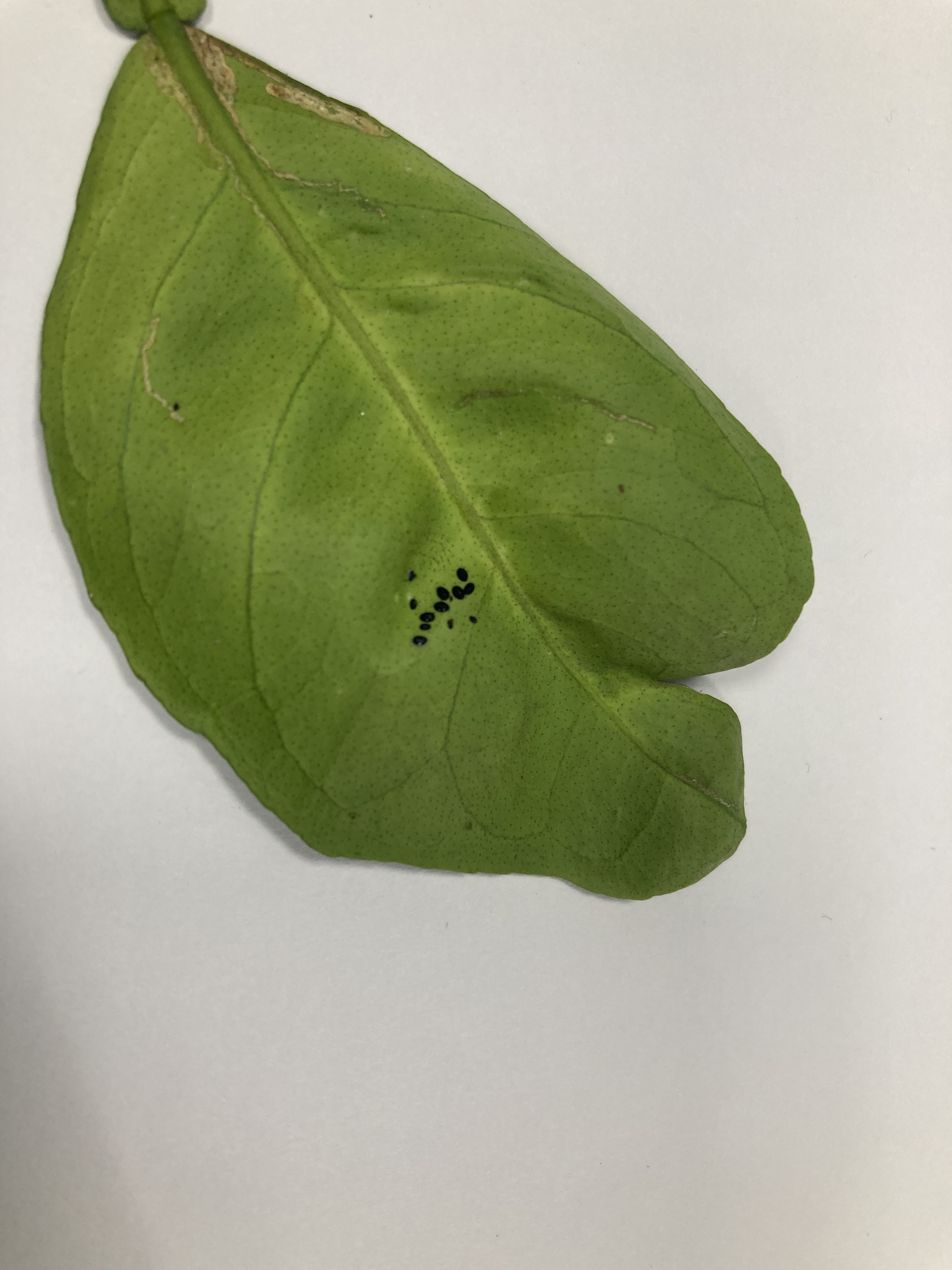

Supplement: Supplementary file 1 [file mmc1.zip › Sweetorange Sample Dataset/Annotation/Spiny_whitefly (2).jpg]

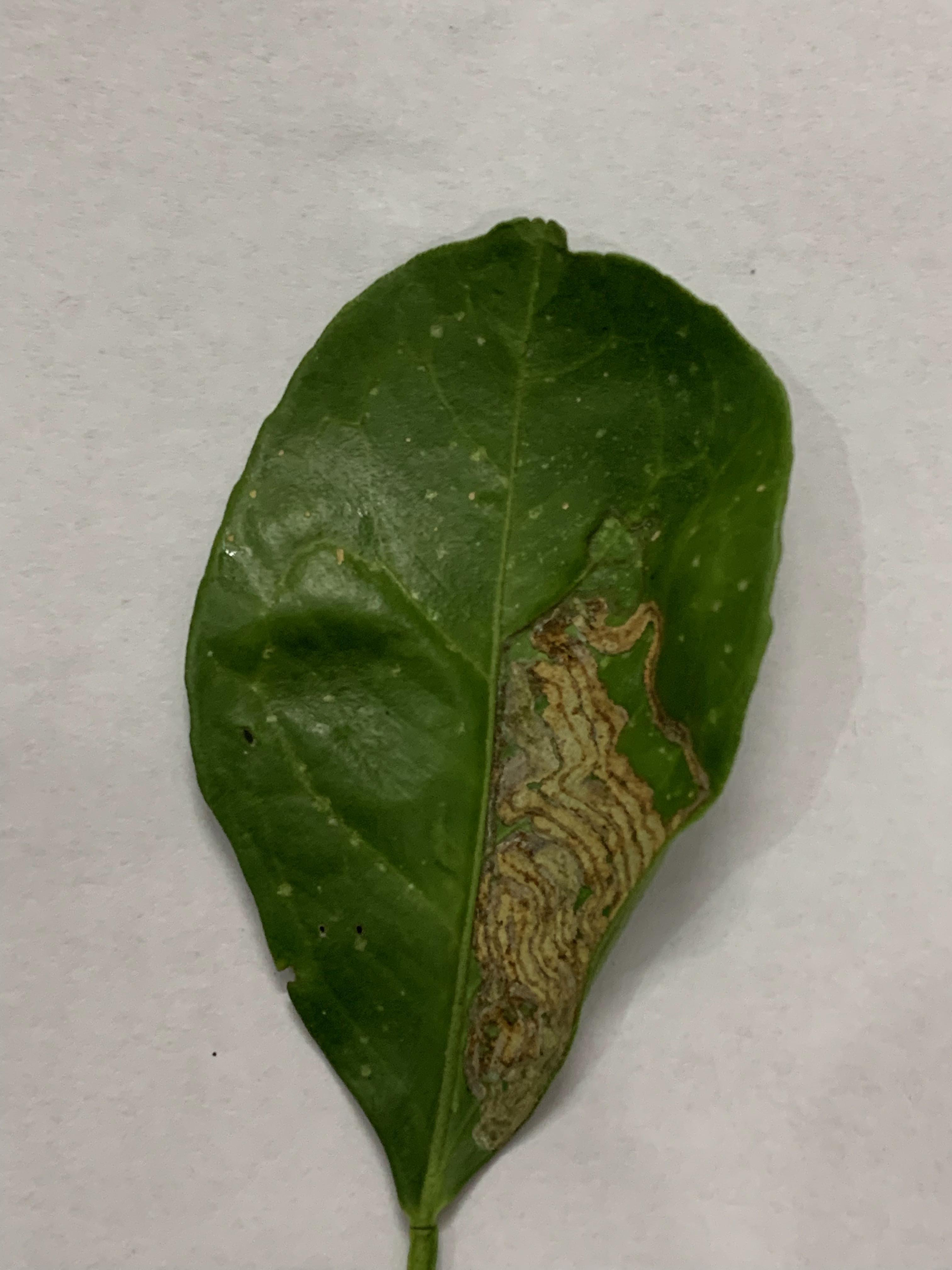

Supplement: Supplementary file 1 [file mmc1.zip › Sweetorange Sample Dataset/Annotation/Foliage_damaged (3).jpg]

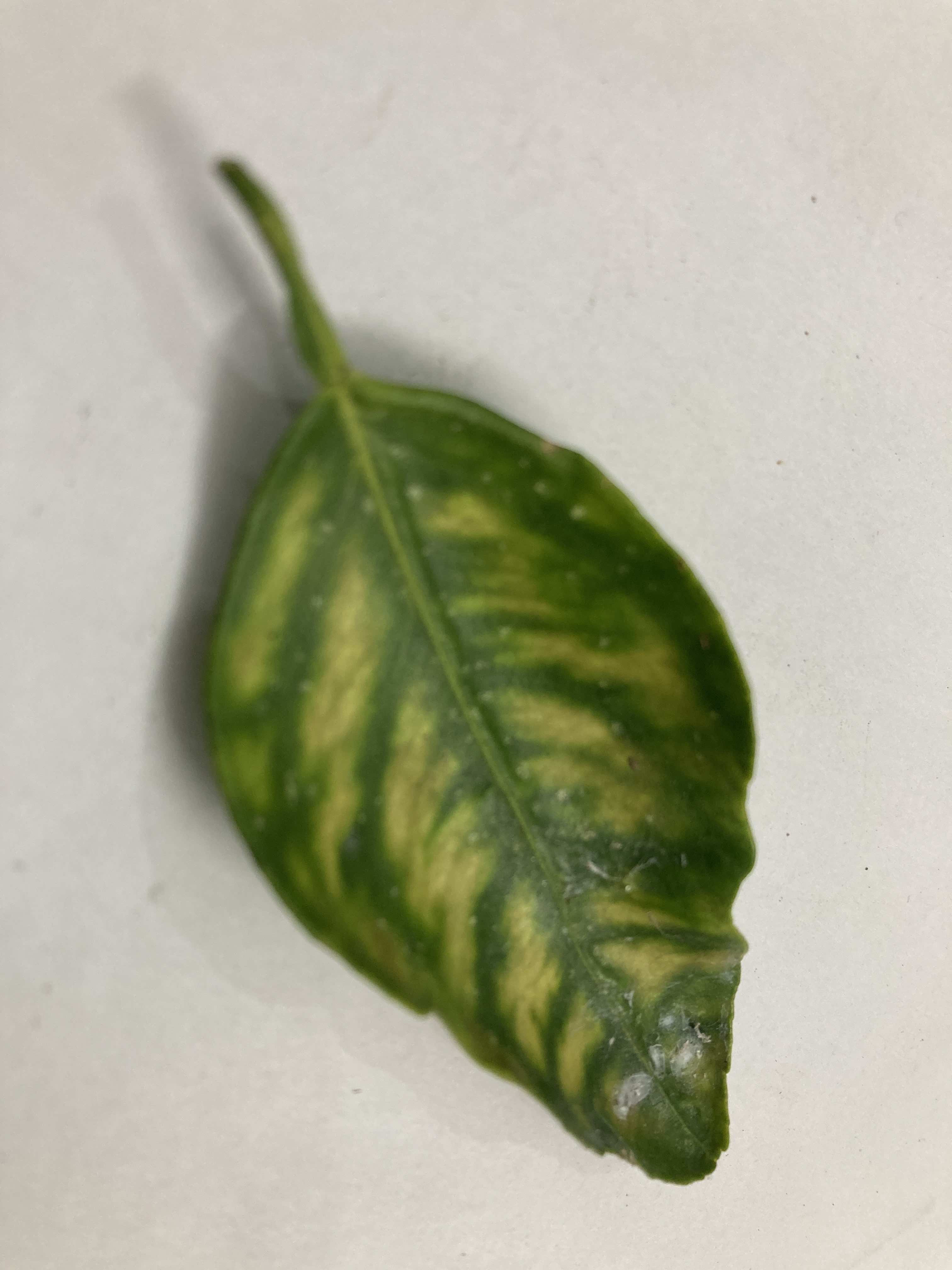

Supplement: Supplementary file 1 [file mmc1.zip › Sweetorange Sample Dataset/Annotation/Citrus_greening (2).jpg]

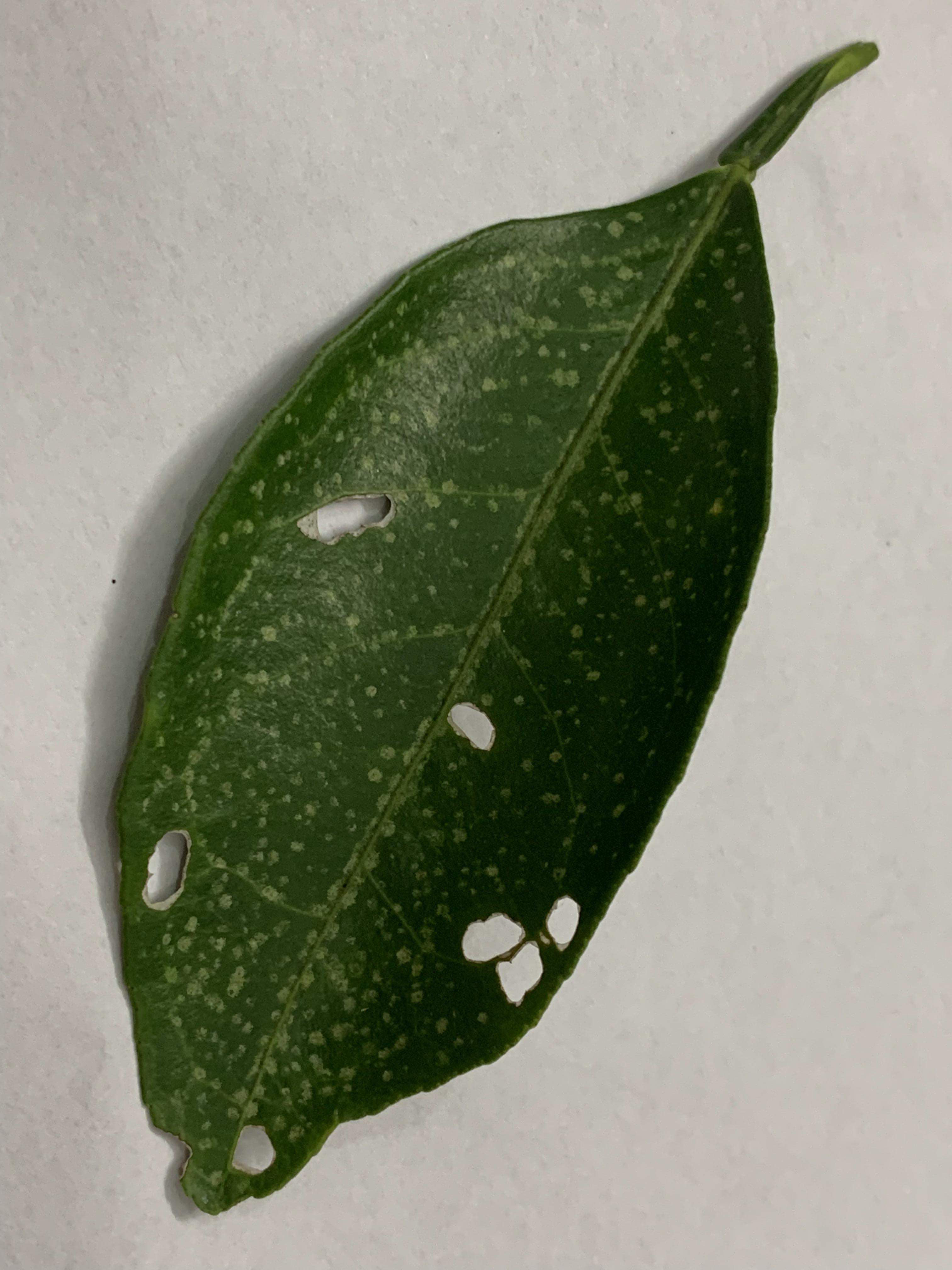

Supplement: Supplementary file 1 [file mmc1.zip › Sweetorange Sample Dataset/Annotation/Shot_hole (4).jpg]

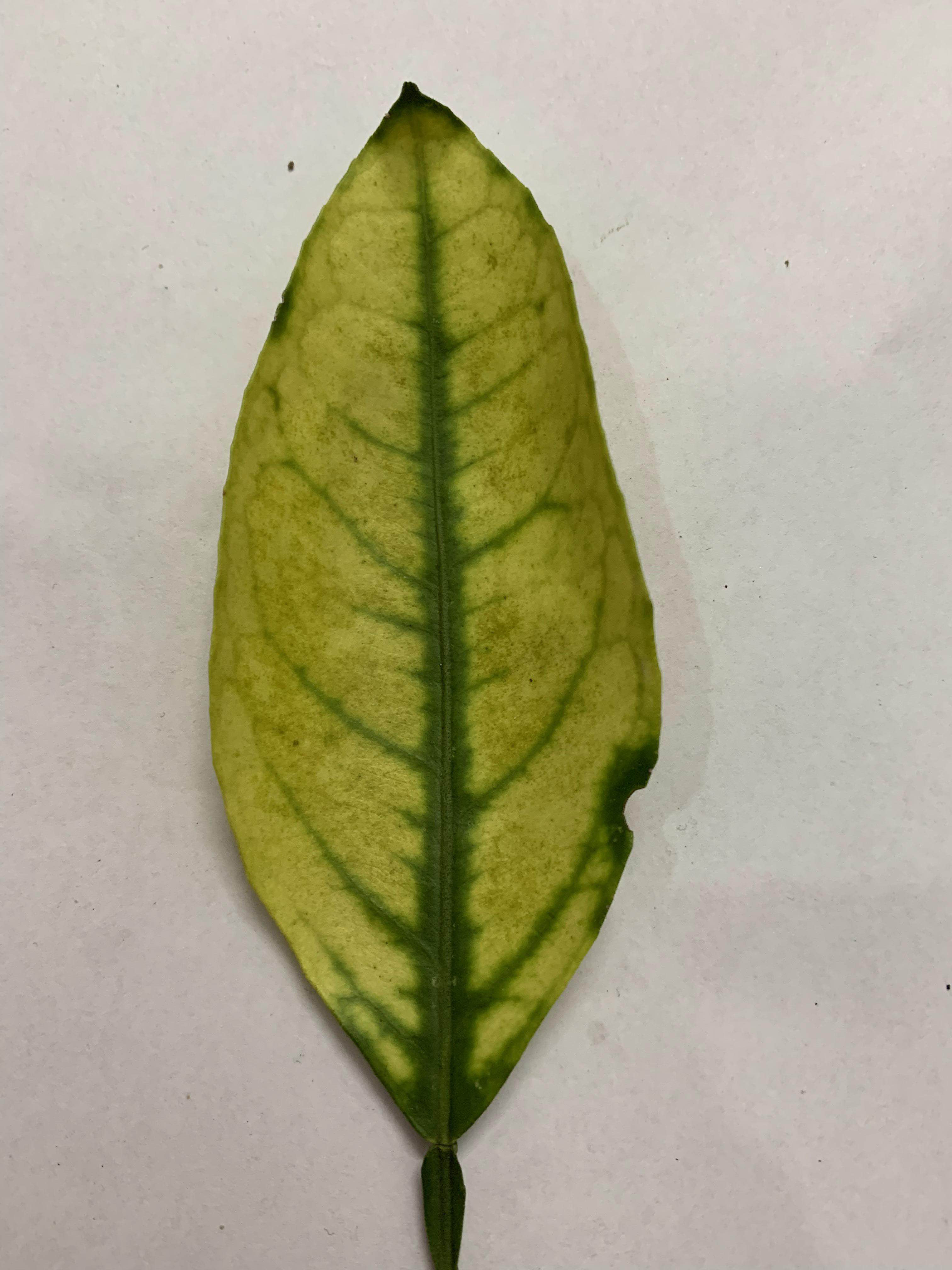

Supplement: Supplementary file 1 [file mmc1.zip › Sweetorange Sample Dataset/Annotation/Yellow_dragon (1).jpg]

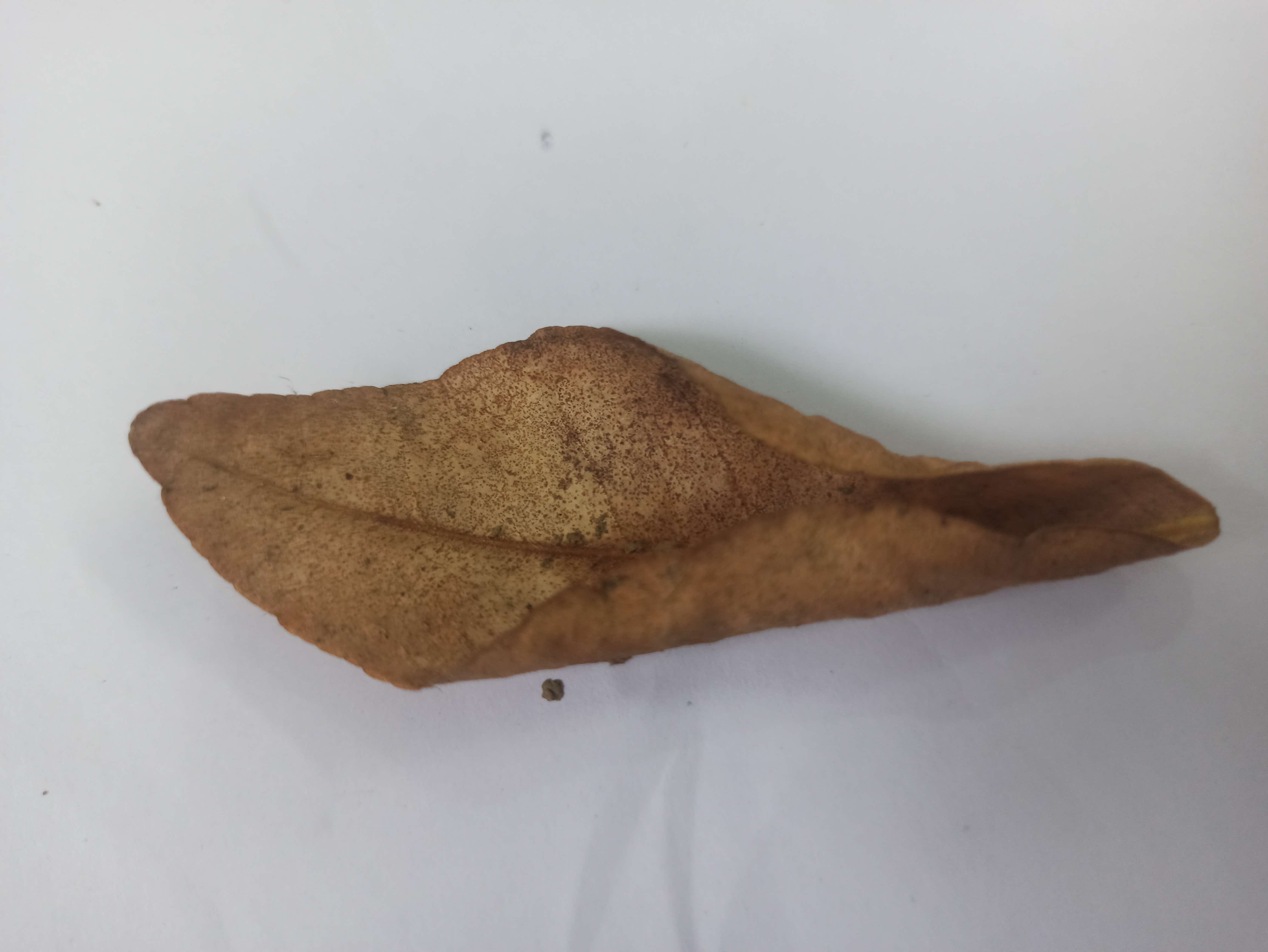

Supplement: Supplementary file 1 [file mmc1.zip › Sweetorange Sample Dataset/Annotation/Die_back (1).jpg]

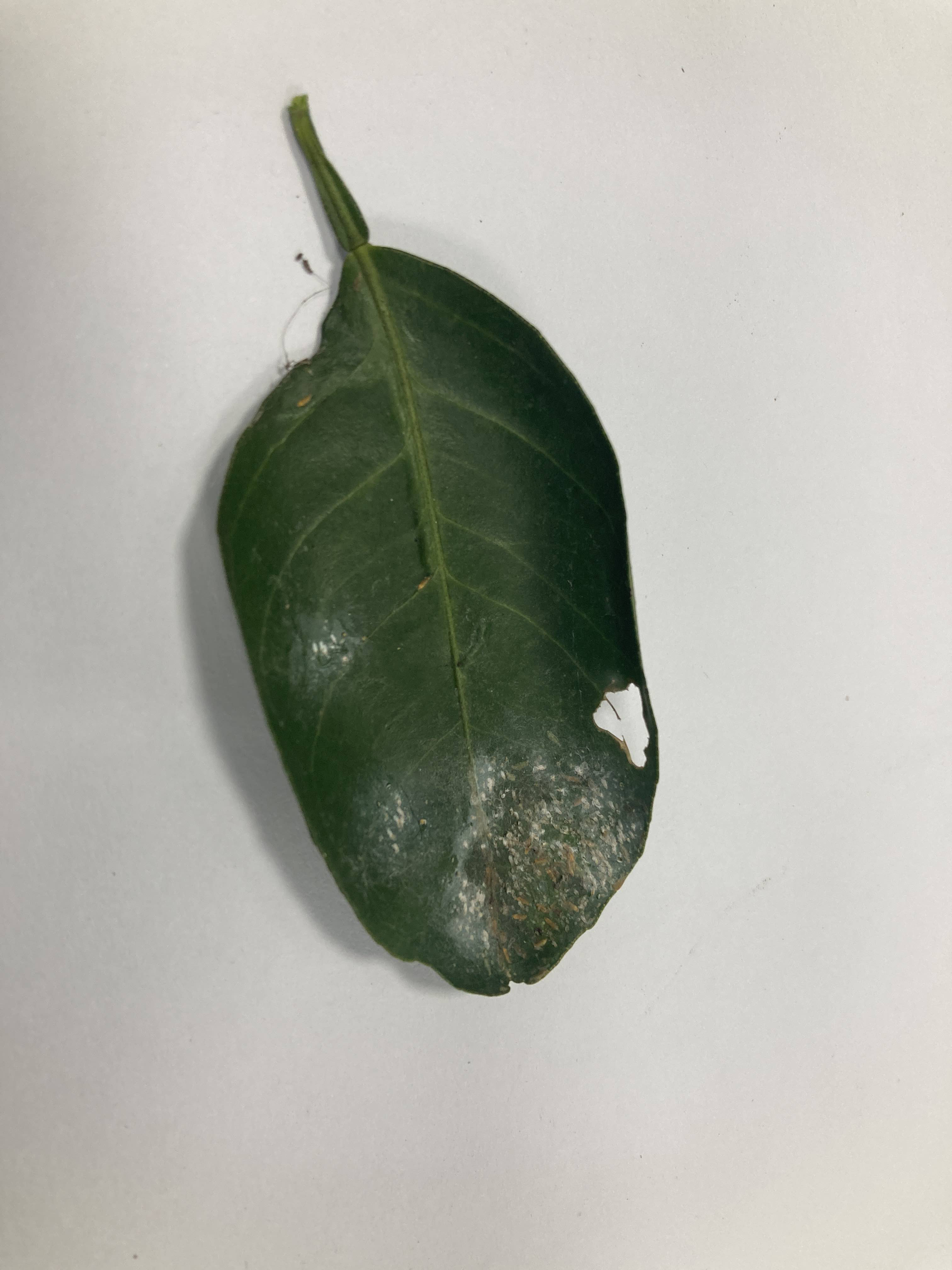

Supplement: Supplementary file 1 [file mmc1.zip › Sweetorange Sample Dataset/Annotation/Citrus_mealybugs (3).jpg]

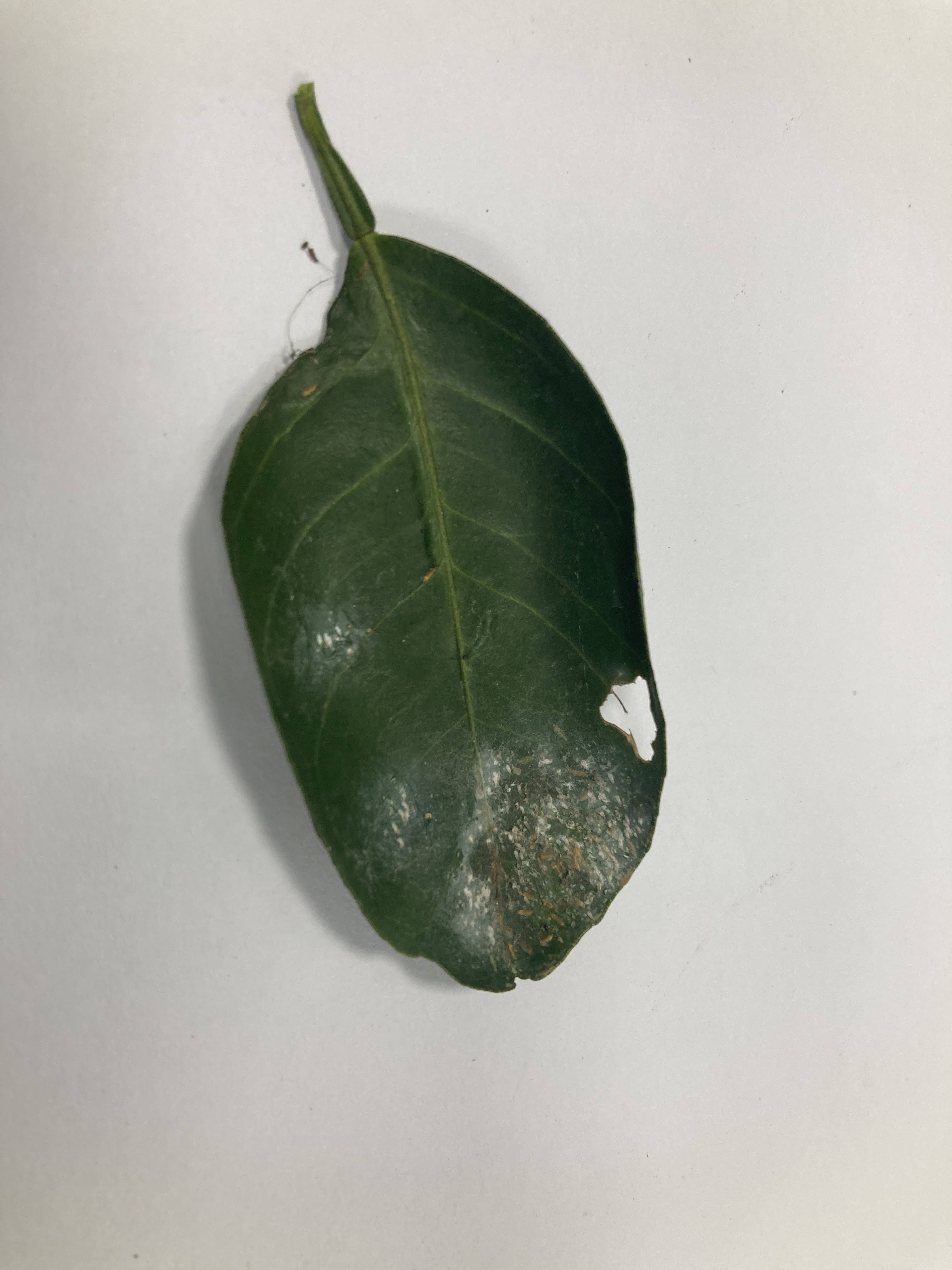

Supplement: Supplementary file 1 [file mmc1.zip › Sweetorange Sample Dataset/Annotation/Citrus_mealybugs (2).jpg]

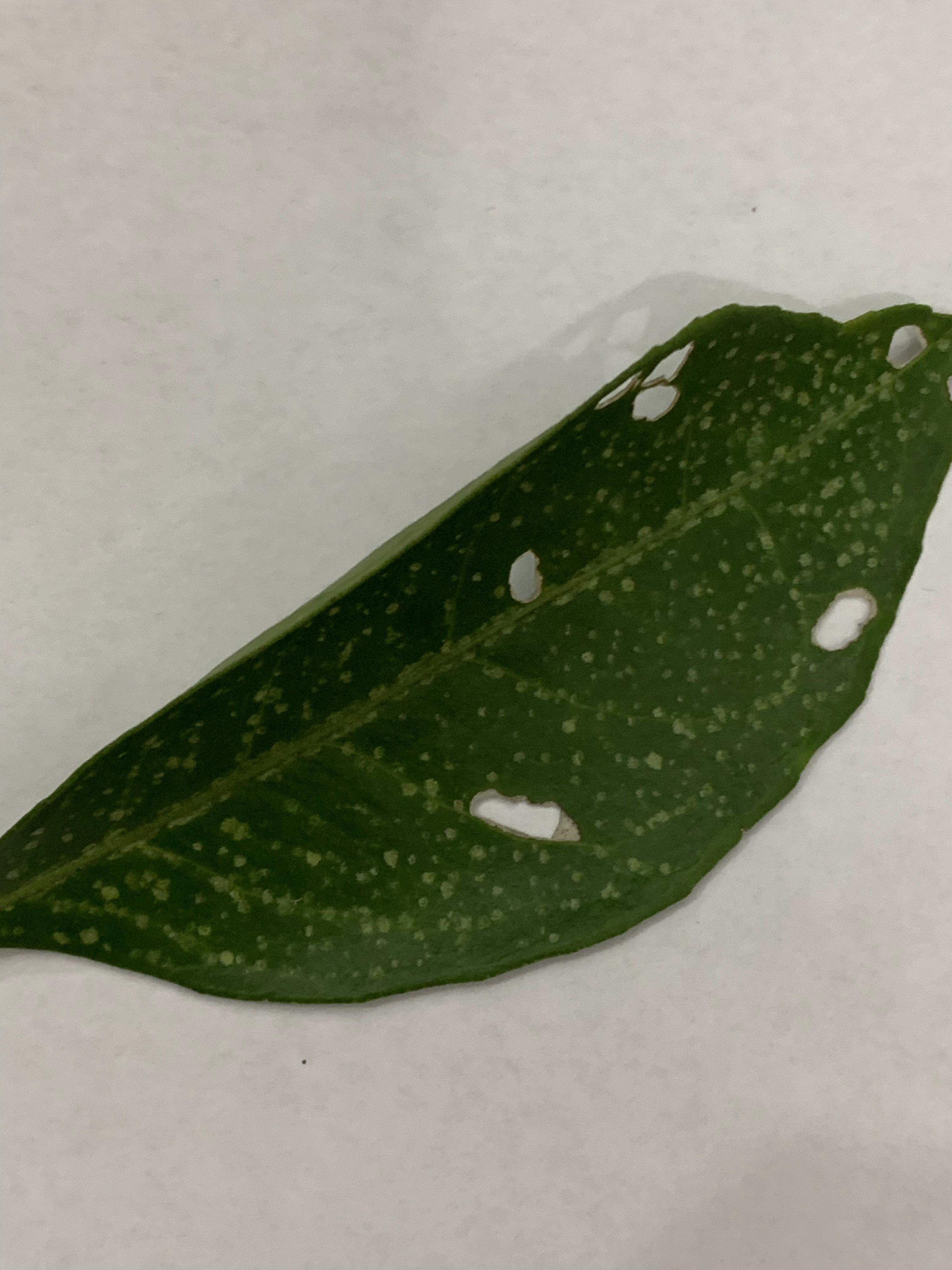

Supplement: Supplementary file 1 [file mmc1.zip › Sweetorange Sample Dataset/Annotation/Shot_hole (5).jpg]

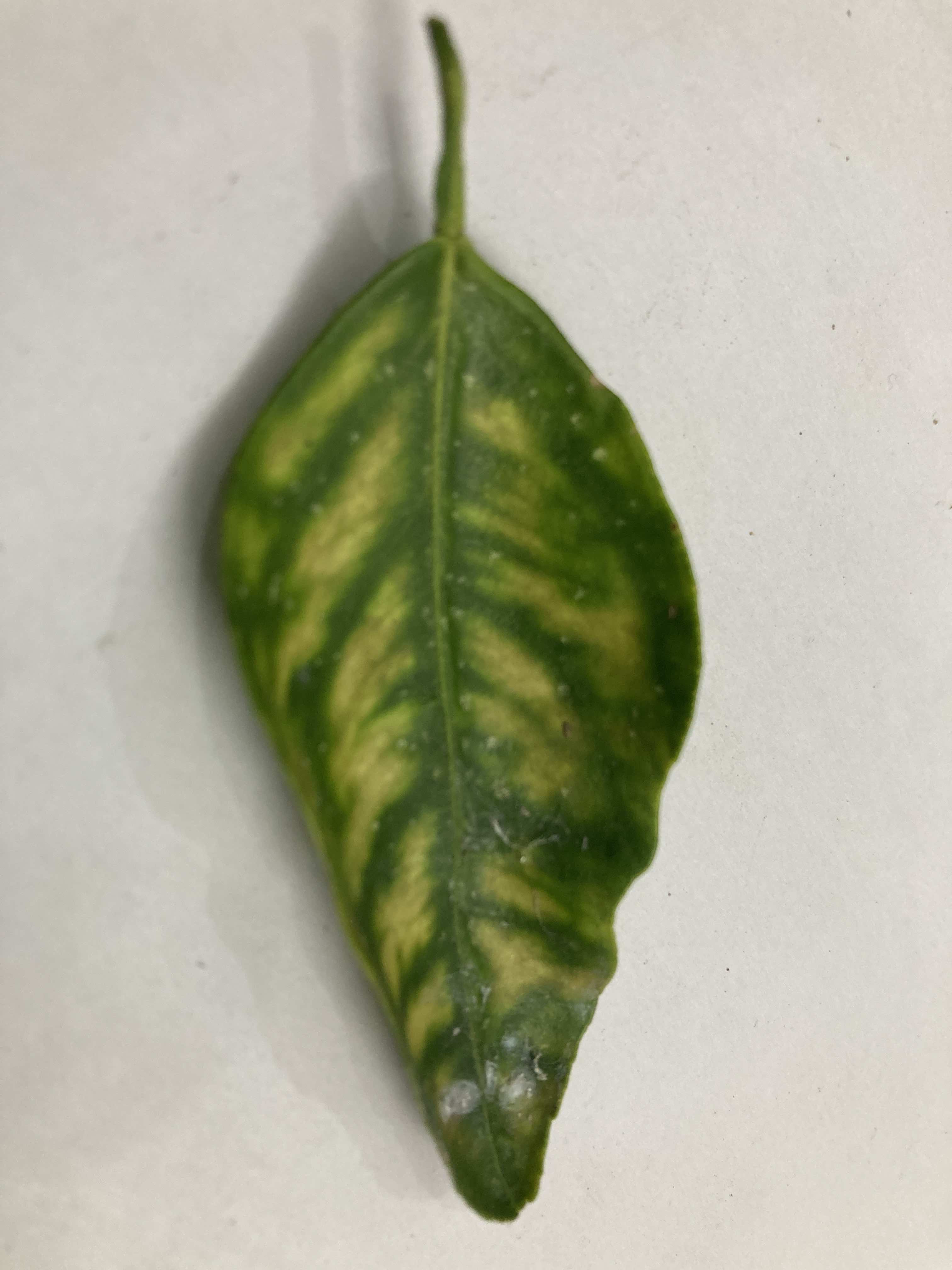

Supplement: Supplementary file 1 [file mmc1.zip › Sweetorange Sample Dataset/Annotation/Citrus_greening (3).jpg]

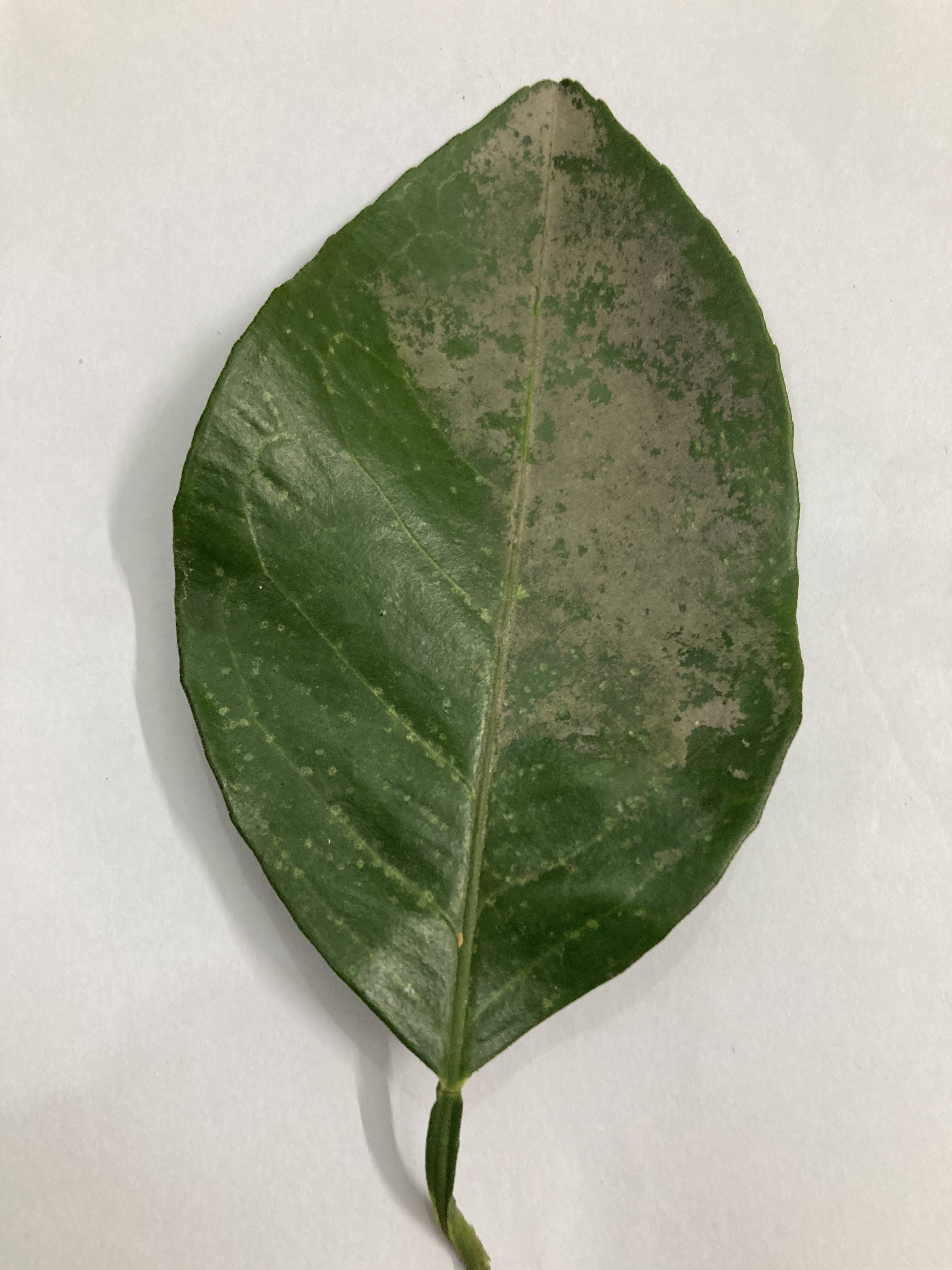

Supplement: Supplementary file 1 [file mmc1.zip › Sweetorange Sample Dataset/Annotation/Powdery_mildew (1).jpg]

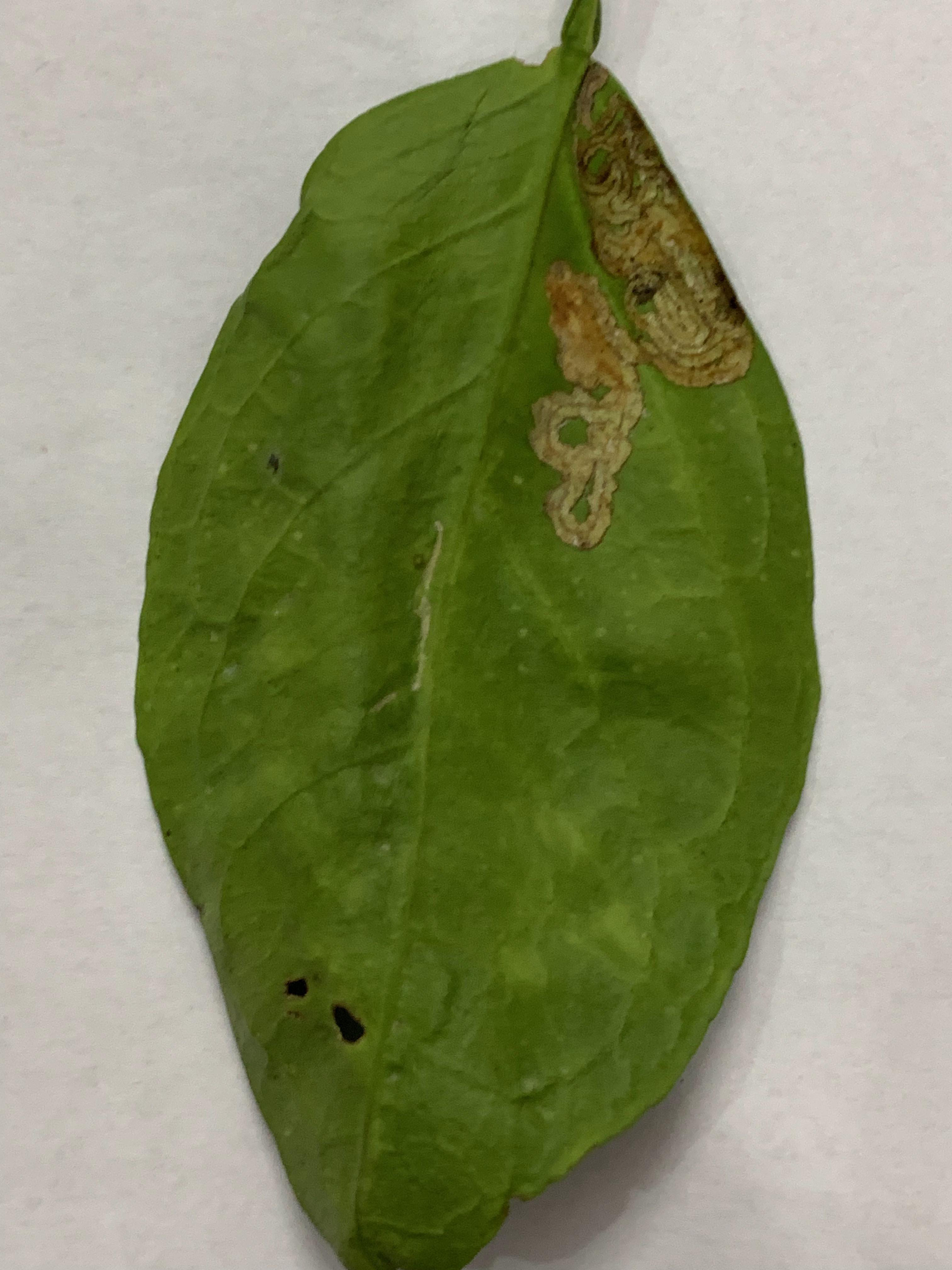

Supplement: Supplementary file 1 [file mmc1.zip › Sweetorange Sample Dataset/Annotation/Foliage_damaged (2).jpg]

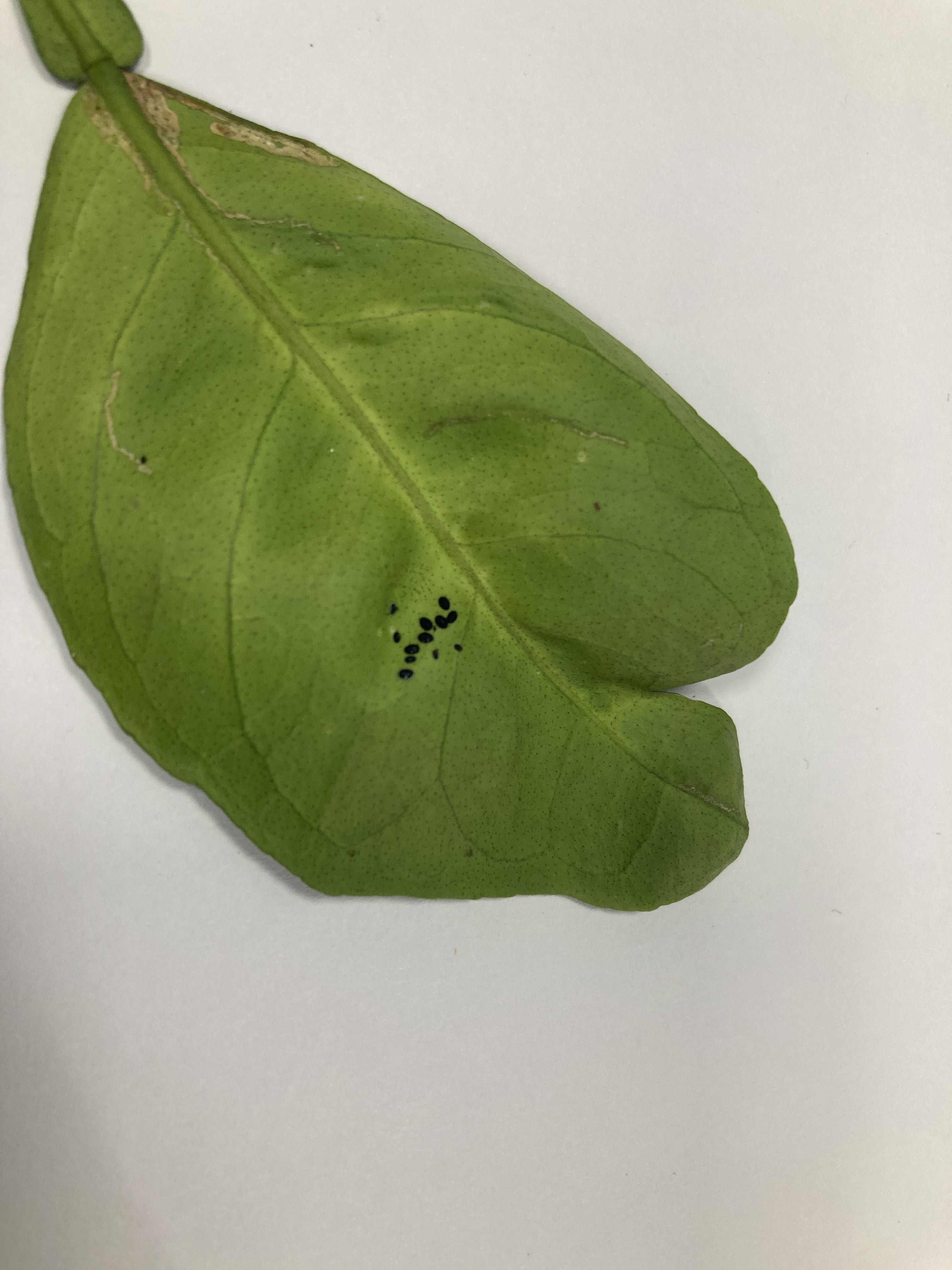

Supplement: Supplementary file 1 [file mmc1.zip › Sweetorange Sample Dataset/Annotation/Spiny_whitefly (3).jpg]

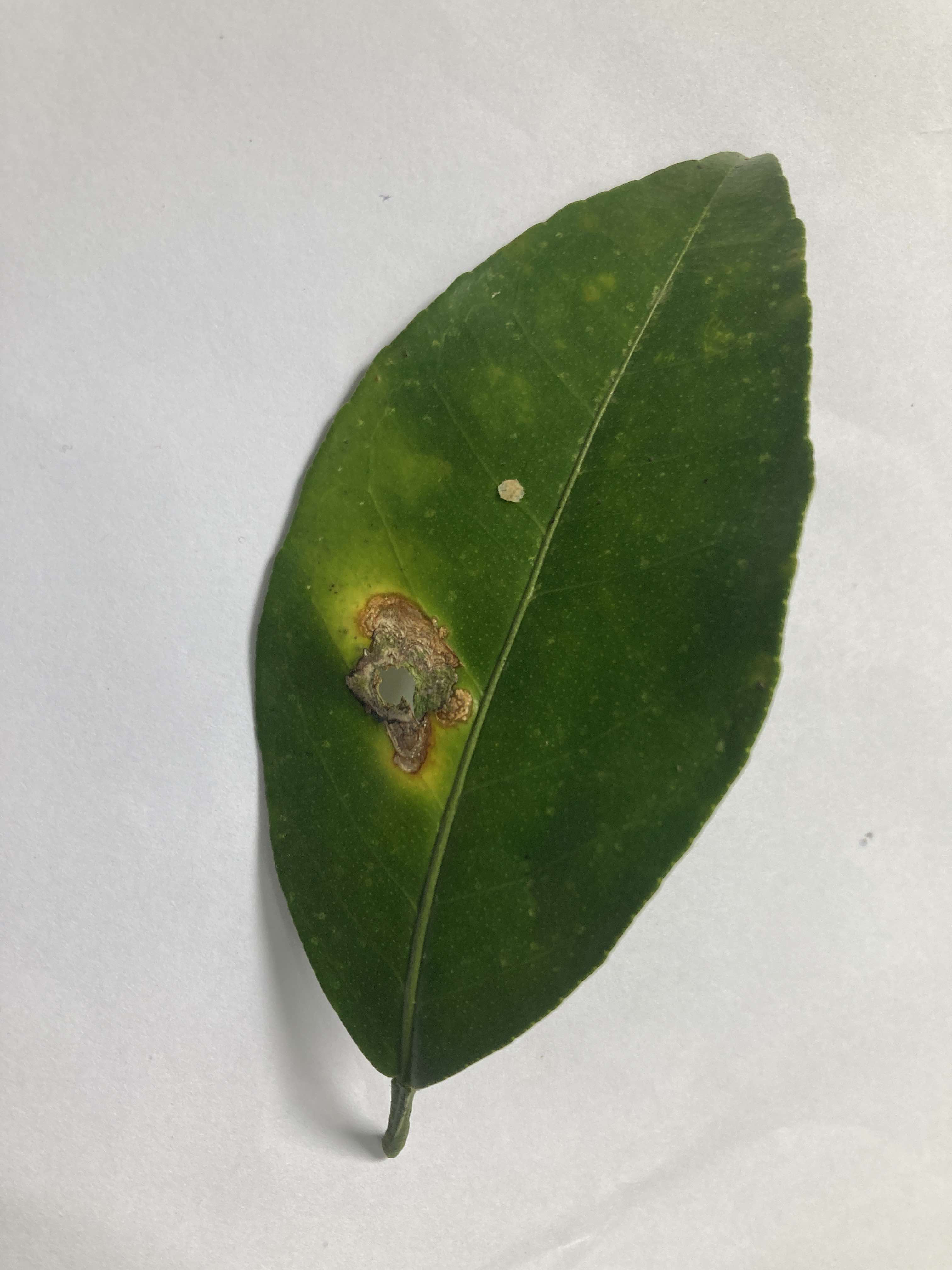

Supplement: Supplementary file 1 [file mmc1.zip › Sweetorange Sample Dataset/Annotation/Citrus_canker (3).jpg]

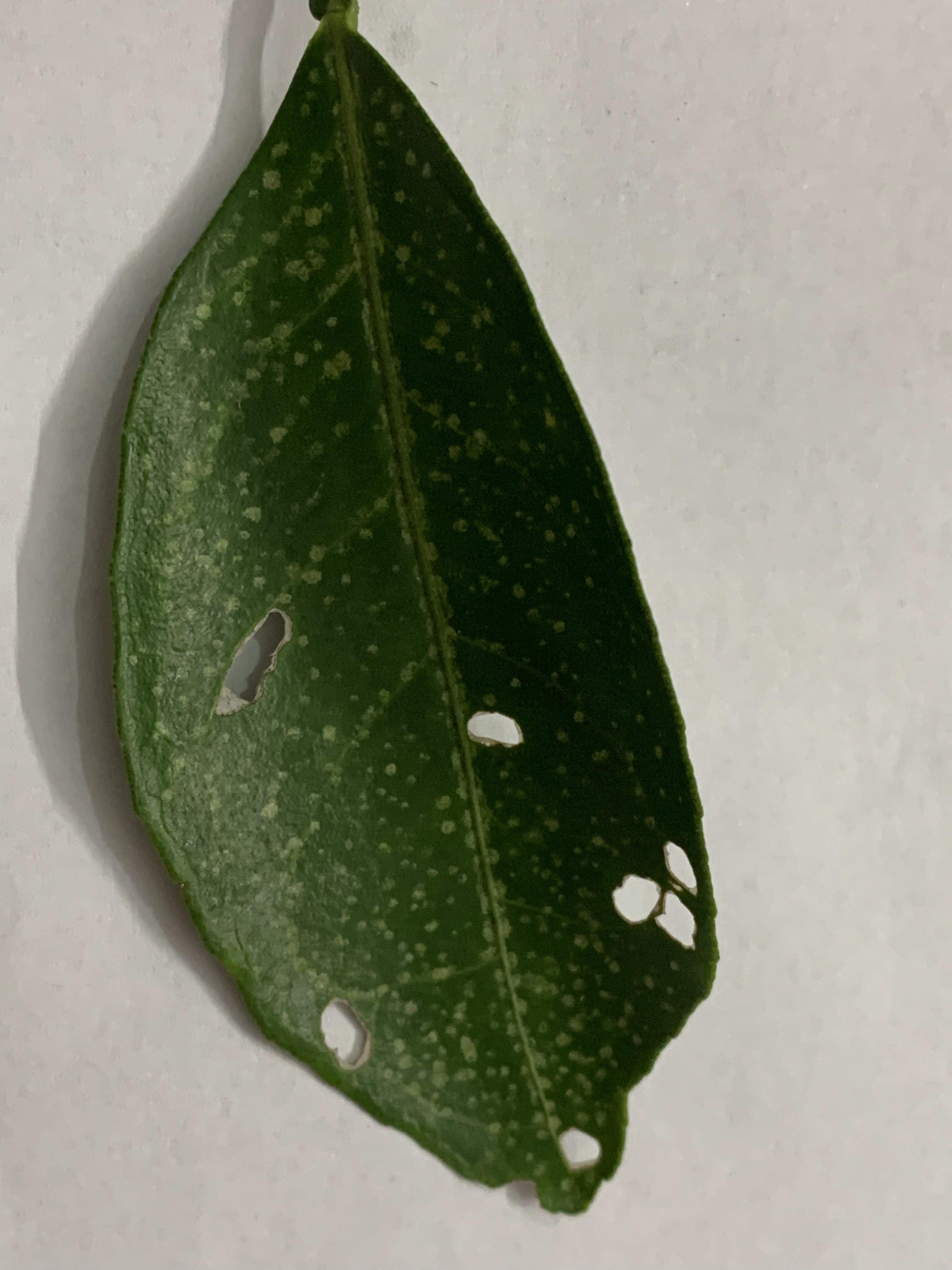

Supplement: Supplementary file 1 [file mmc1.zip › Sweetorange Sample Dataset/Annotation/Shot_hole (2).jpg]

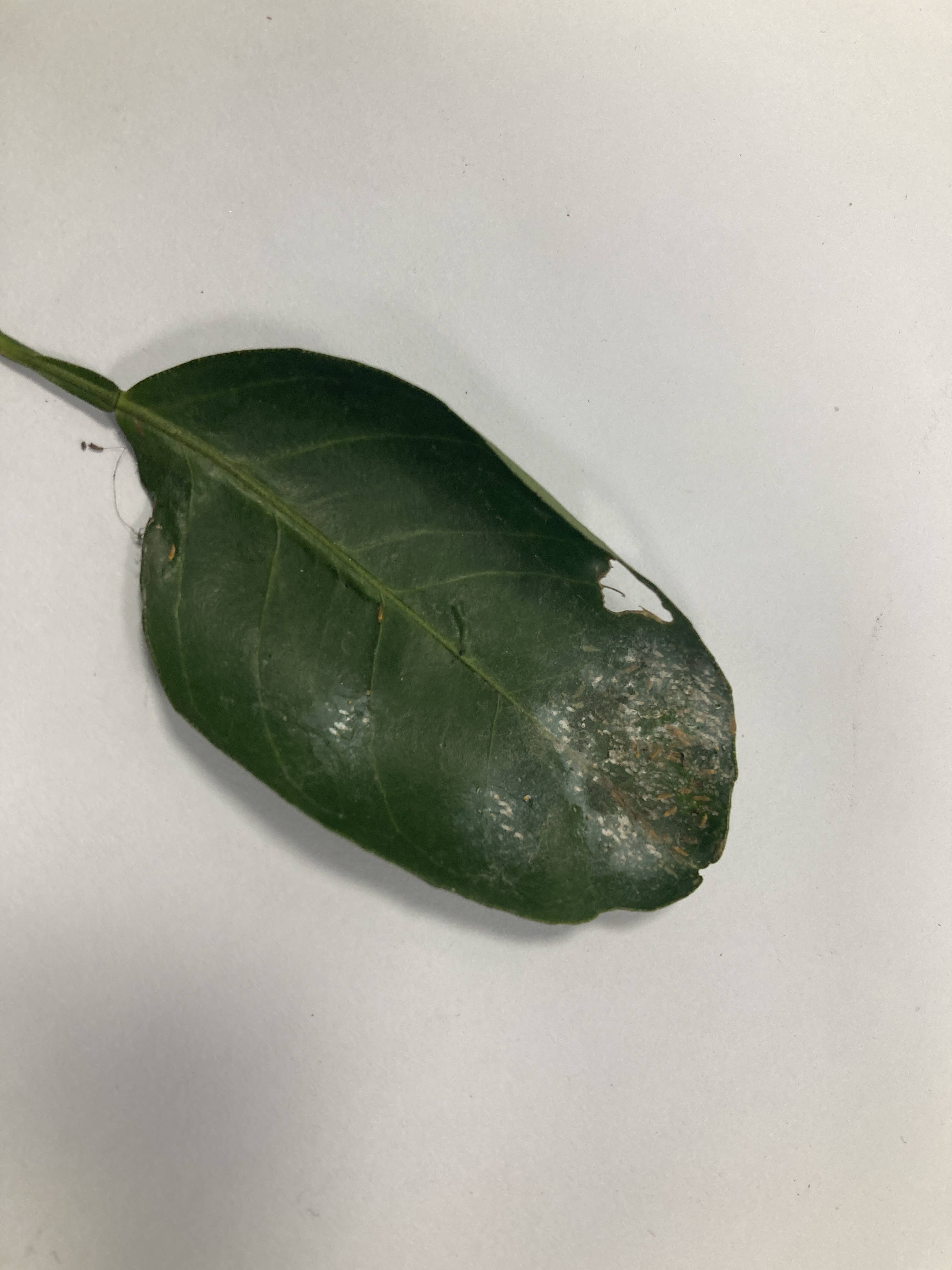

Supplement: Supplementary file 1 [file mmc1.zip › Sweetorange Sample Dataset/Annotation/Citrus_mealybugs (5).jpg]

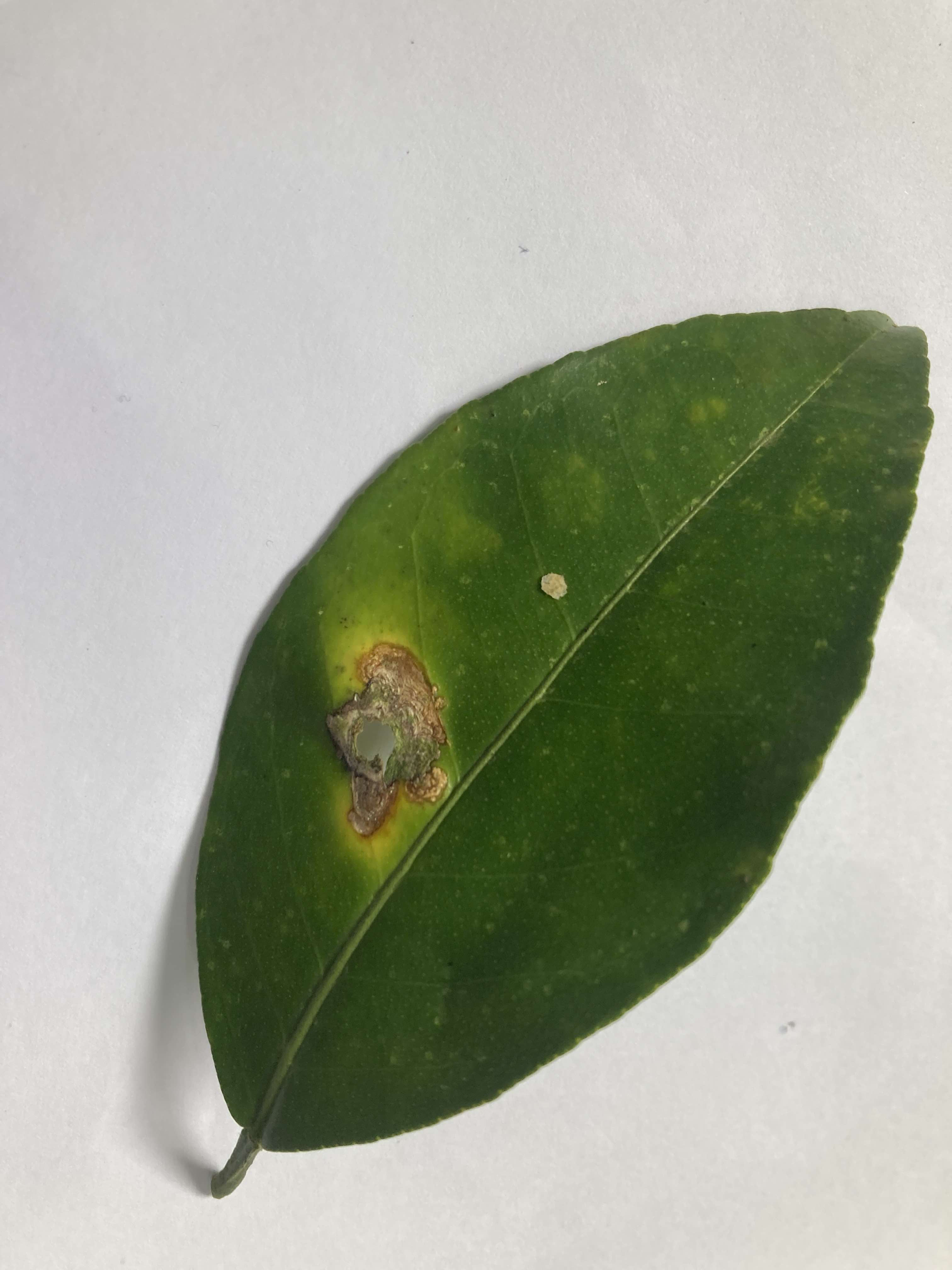

Supplement: Supplementary file 1 [file mmc1.zip › Sweetorange Sample Dataset/Annotation/Citrus_canker (4).jpg]

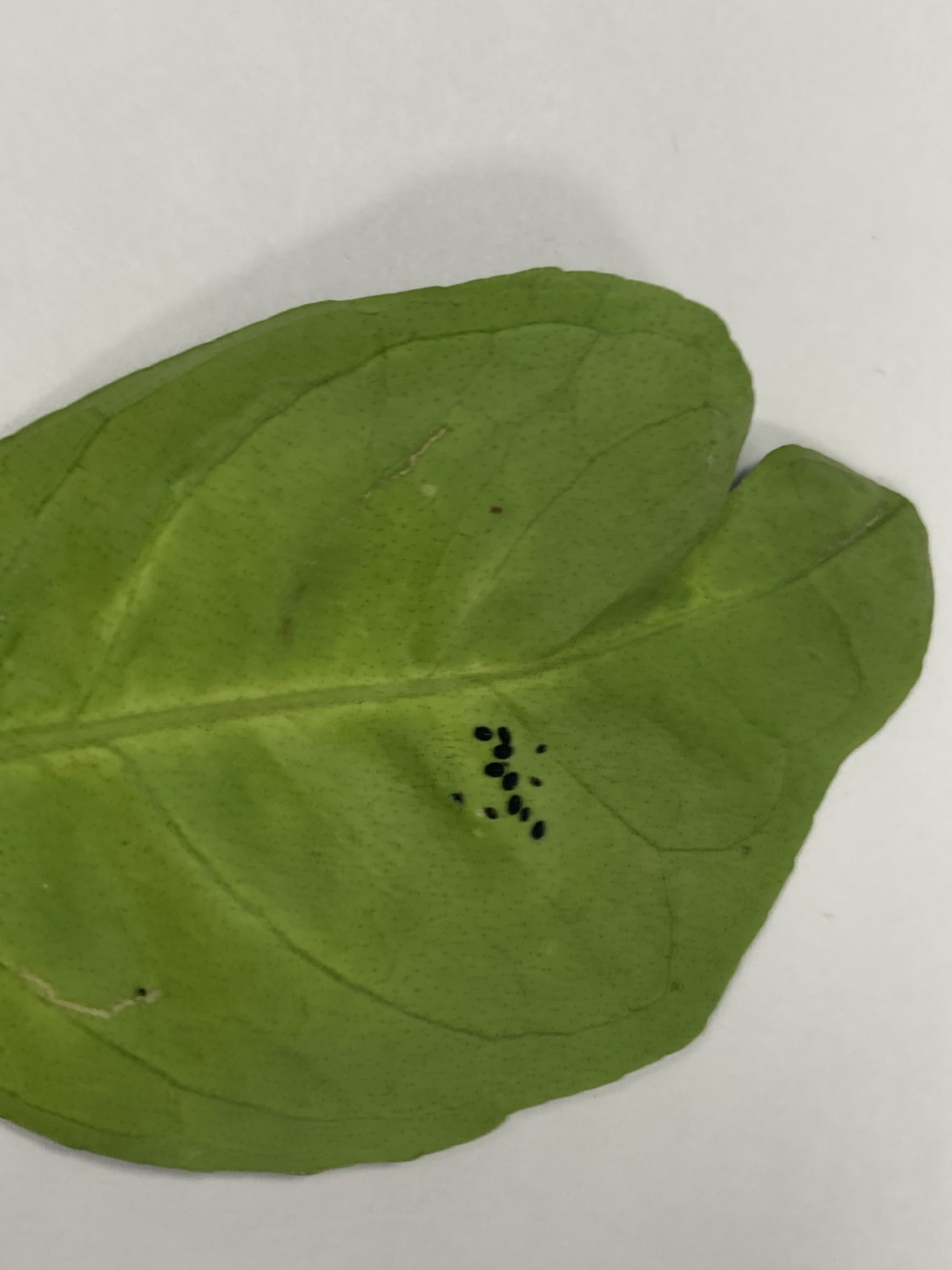

Supplement: Supplementary file 1 [file mmc1.zip › Sweetorange Sample Dataset/Annotation/Spiny_whitefly (4).jpg]

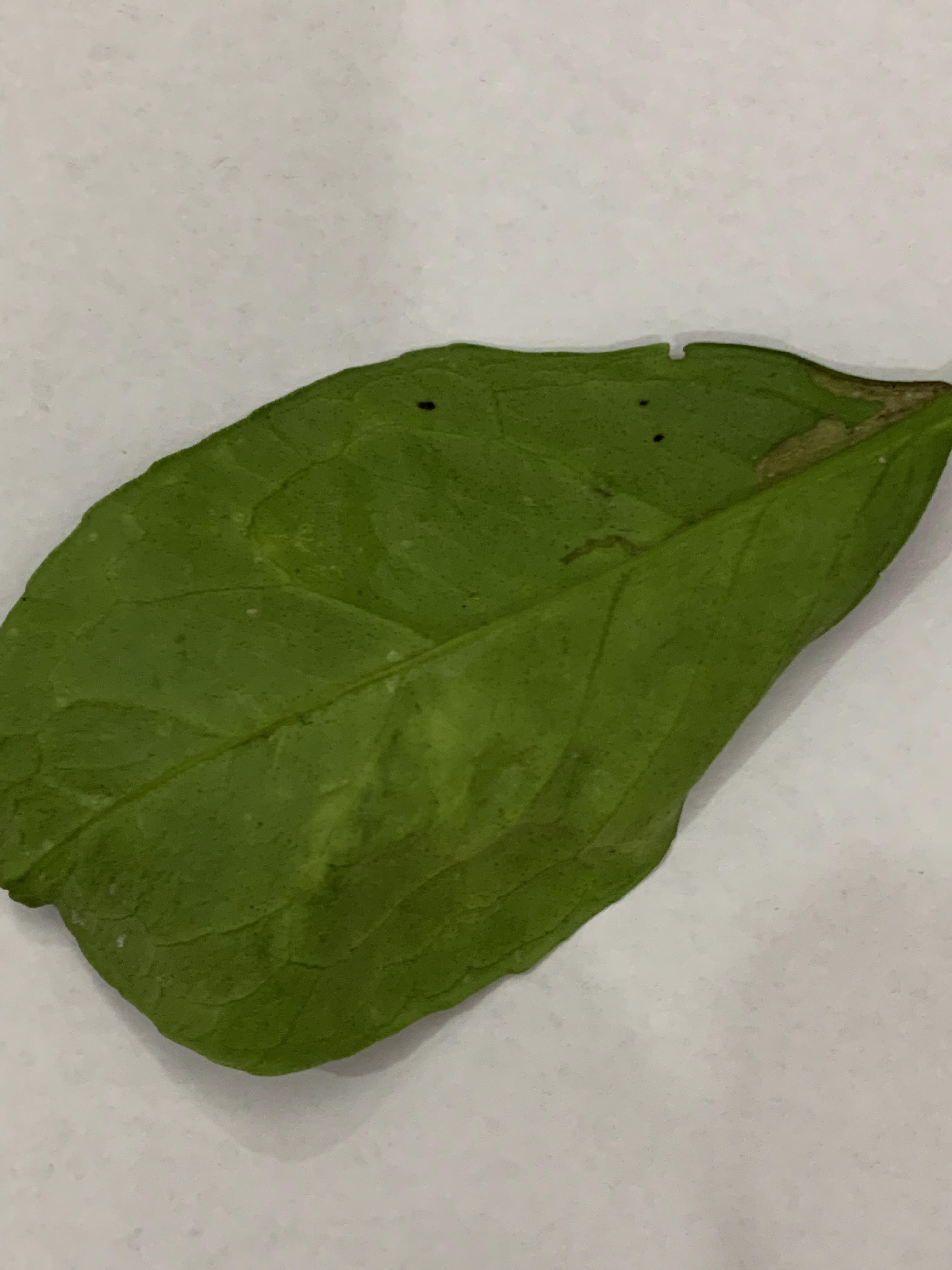

Supplement: Supplementary file 1 [file mmc1.zip › Sweetorange Sample Dataset/Annotation/Foliage_damaged (5).jpg]

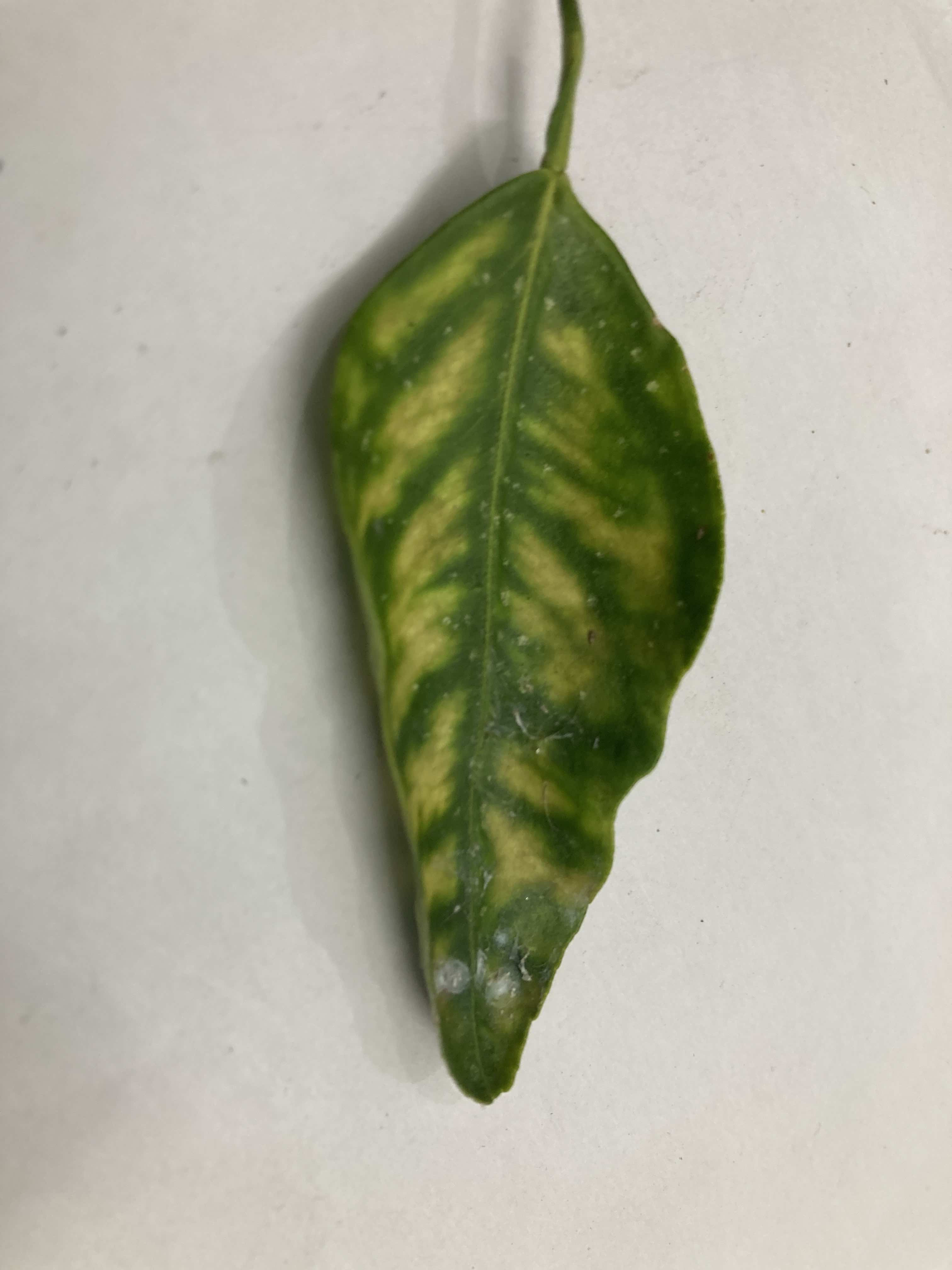

Supplement: Supplementary file 1 [file mmc1.zip › Sweetorange Sample Dataset/Annotation/Citrus_greening (4).jpg]

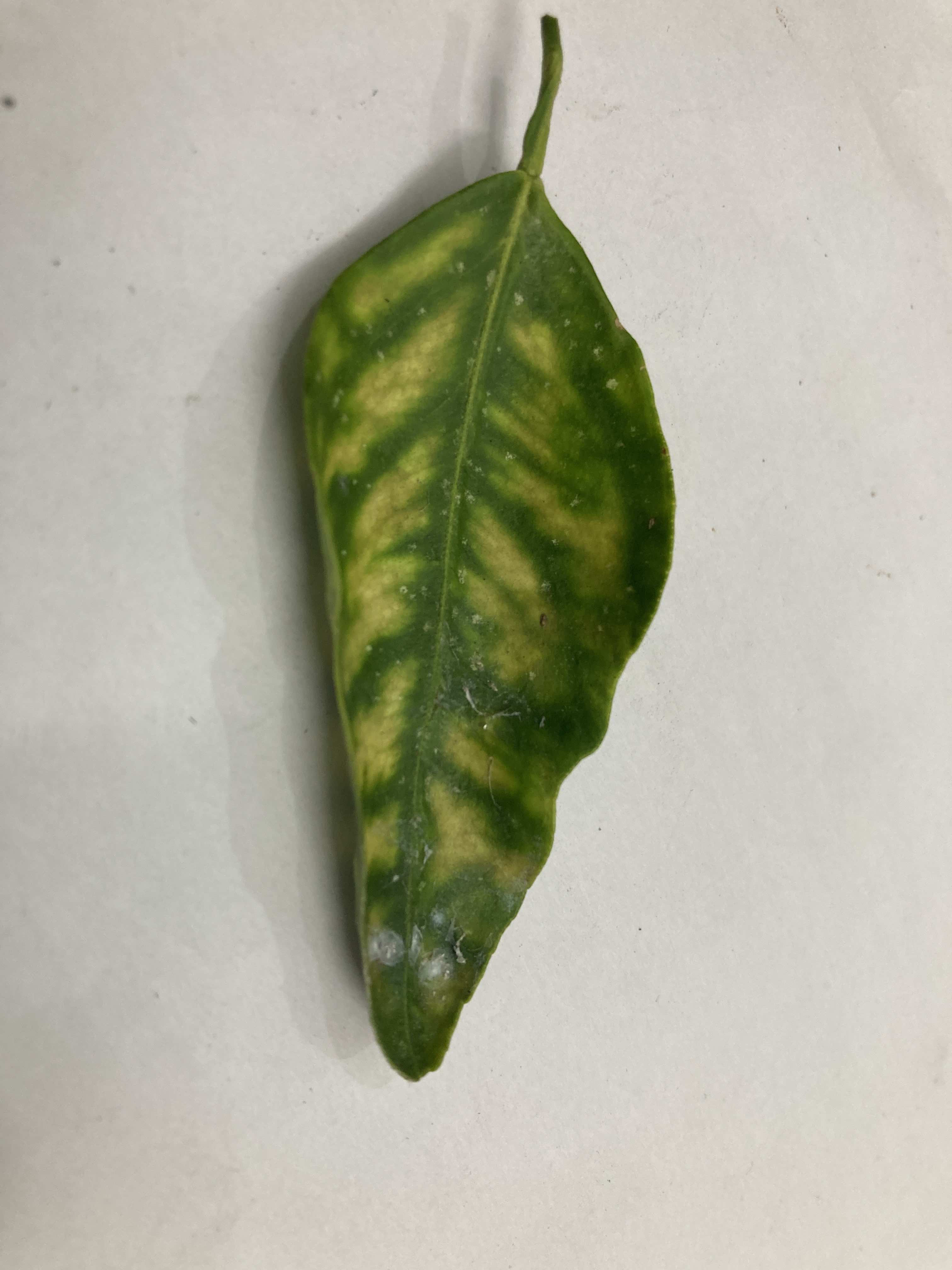

Supplement: Supplementary file 1 [file mmc1.zip › Sweetorange Sample Dataset/Annotation/Citrus_greening (5).jpg]

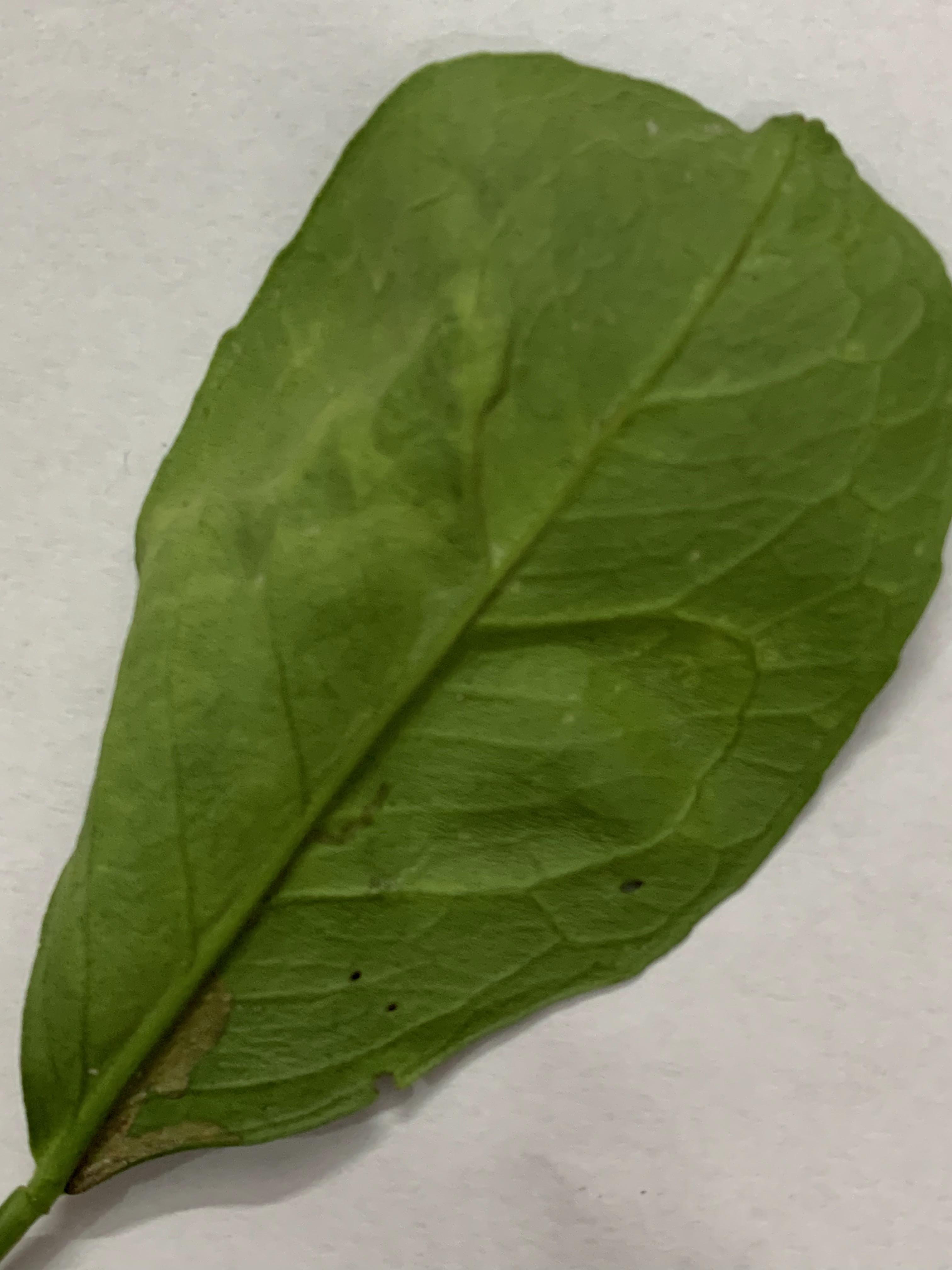

Supplement: Supplementary file 1 [file mmc1.zip › Sweetorange Sample Dataset/Annotation/Foliage_damaged (4).jpg]

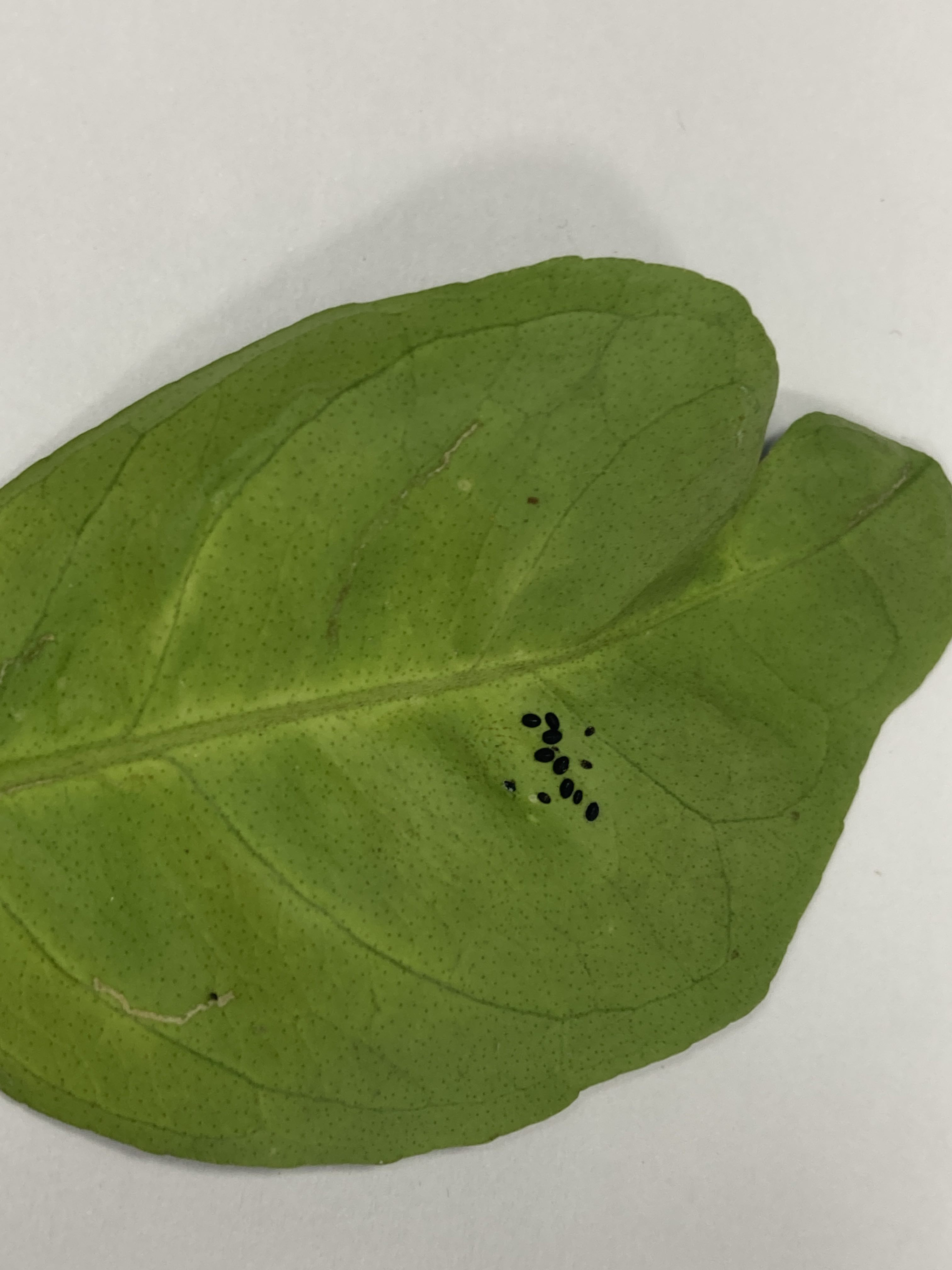

Supplement: Supplementary file 1 [file mmc1.zip › Sweetorange Sample Dataset/Annotation/Spiny_whitefly (5).jpg]

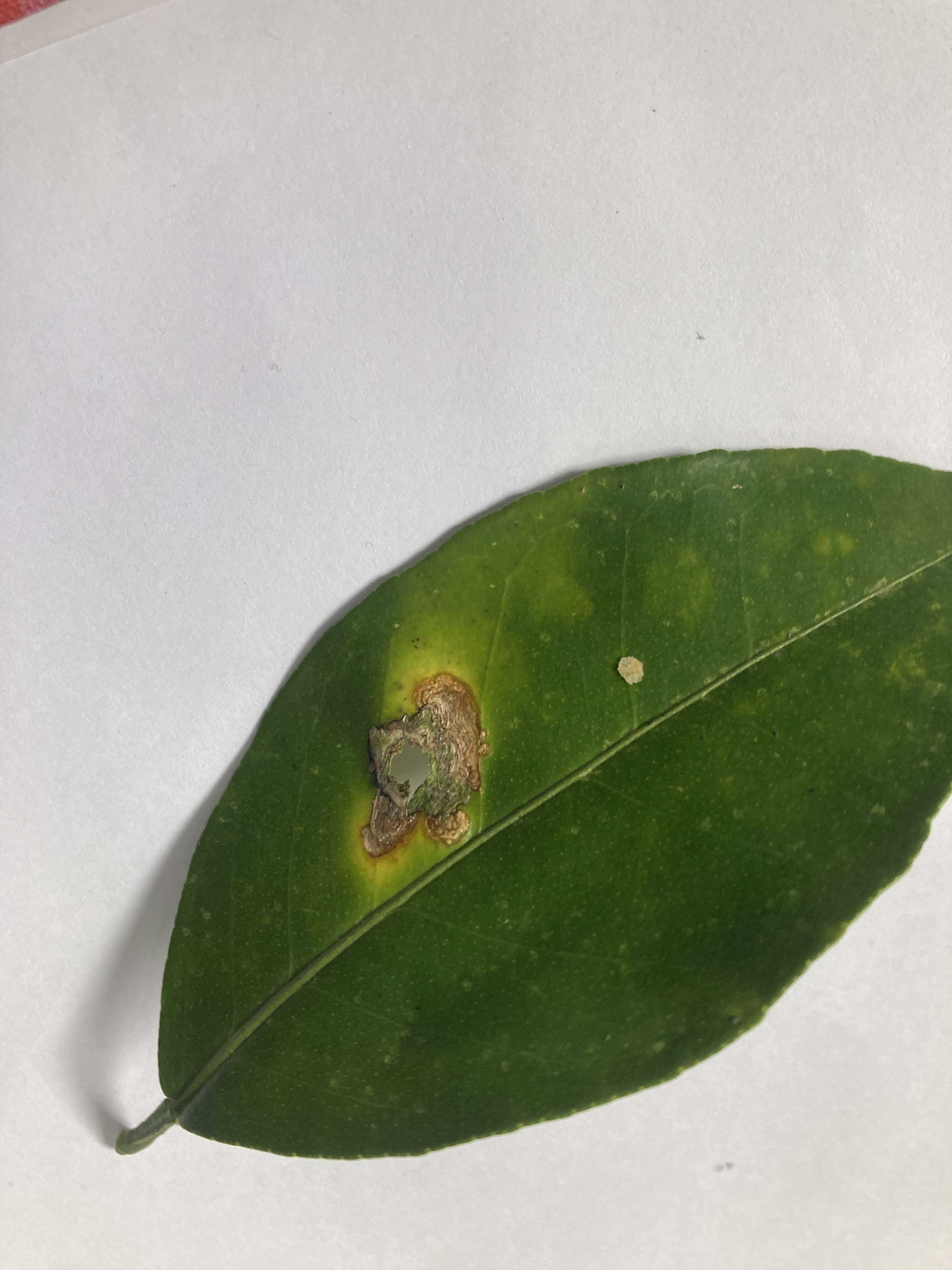

Supplement: Supplementary file 1 [file mmc1.zip › Sweetorange Sample Dataset/Annotation/Citrus_canker (5).jpg]

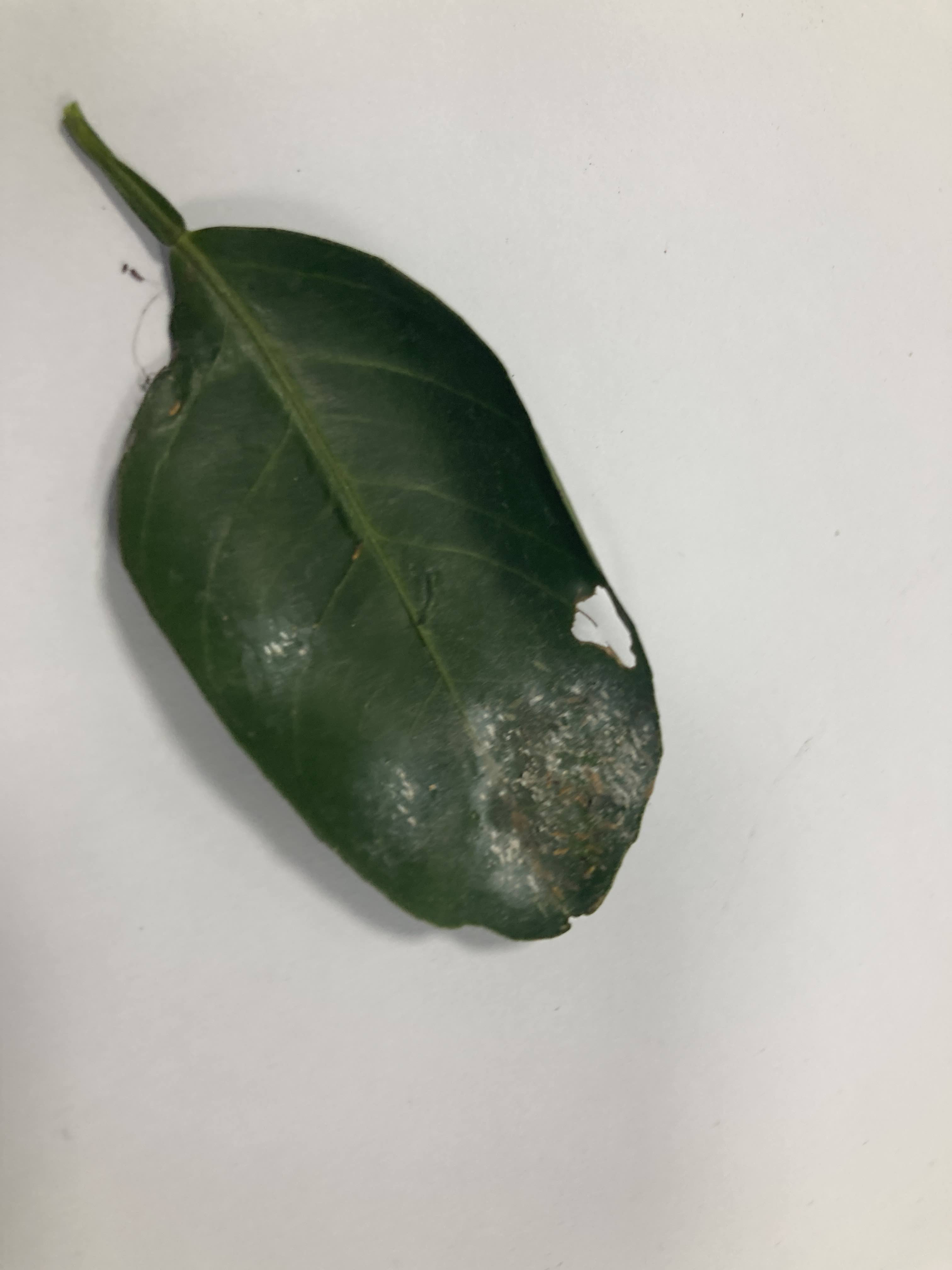

Supplement: Supplementary file 1 [file mmc1.zip › Sweetorange Sample Dataset/Annotation/Citrus_mealybugs (4).jpg]

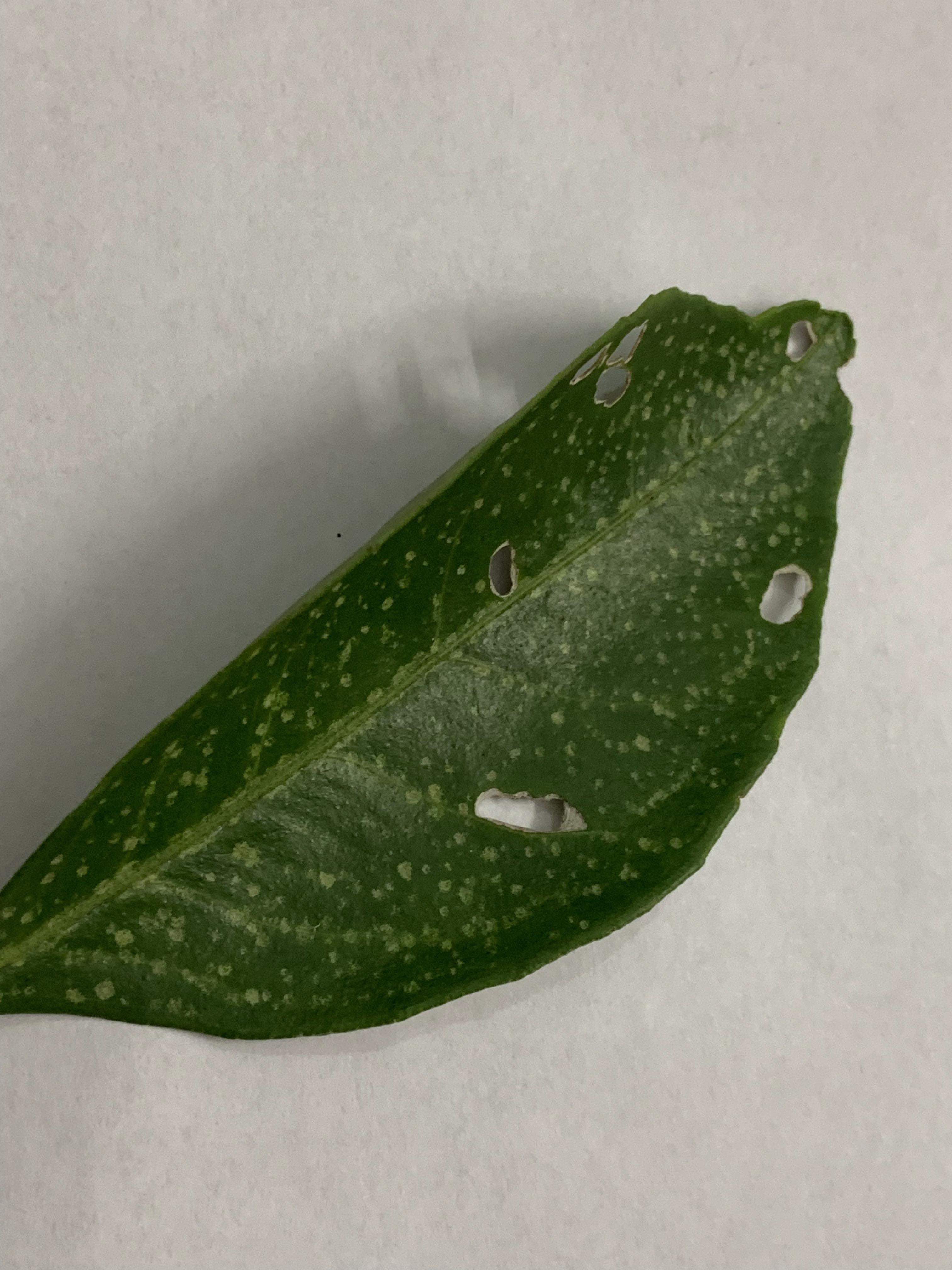

Supplement: Supplementary file 1 [file mmc1.zip › Sweetorange Sample Dataset/Annotation/Shot_hole (3).jpg]

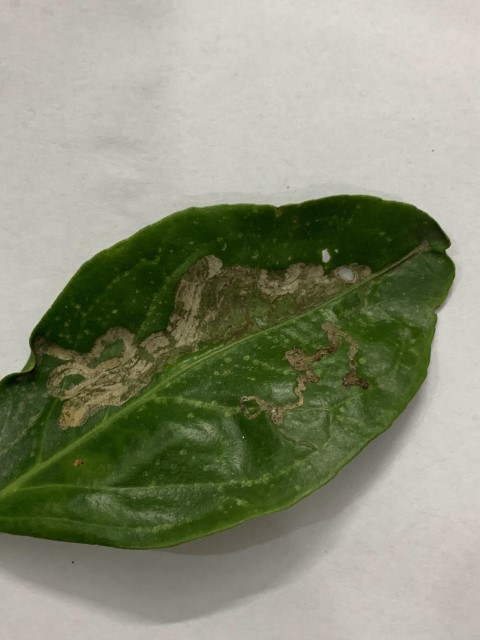

Supplement: Supplementary file 1 [file mmc1.zip › Sweetorange Sample Dataset/Converted Image/Foliage damaged/Con_Foliage Damaged7.jpeg]

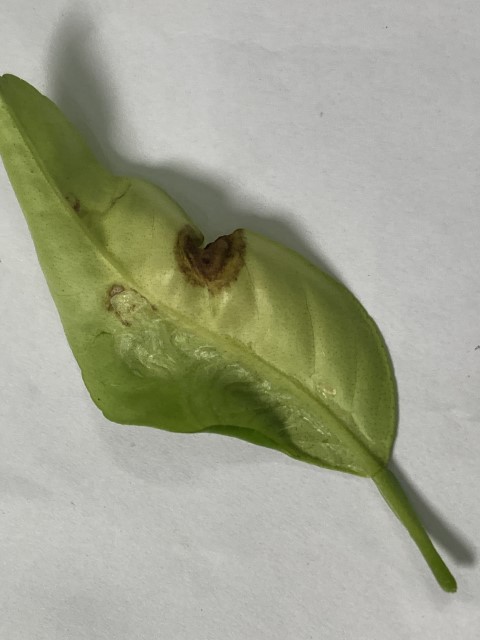

Supplement: Supplementary file 1 [file mmc1.zip › Sweetorange Sample Dataset/Converted Image/Foliage damaged/Con_Foliage Damaged168.jpeg]

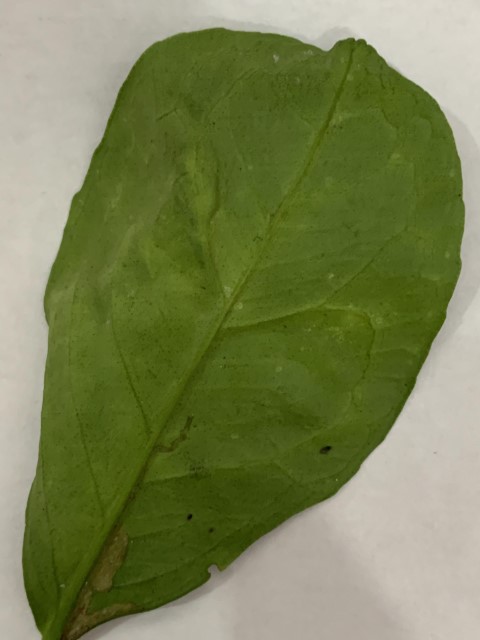

Supplement: Supplementary file 1 [file mmc1.zip › Sweetorange Sample Dataset/Converted Image/Foliage damaged/Con_Foliage Damaged49.jpeg]

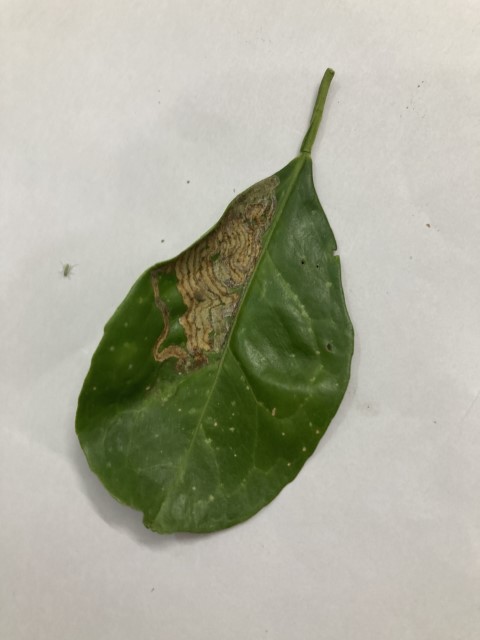

Supplement: Supplementary file 1 [file mmc1.zip › Sweetorange Sample Dataset/Converted Image/Foliage damaged/Con_Foliage Damaged630.jpeg]

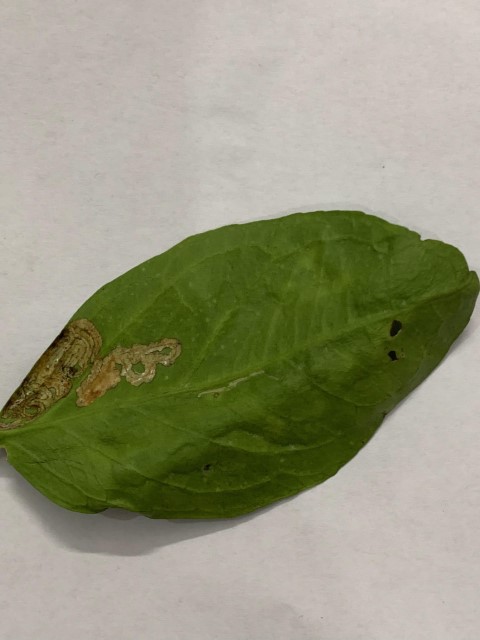

Supplement: Supplementary file 1 [file mmc1.zip › Sweetorange Sample Dataset/Converted Image/Foliage damaged/Con_Foliage Damaged28.jpeg]

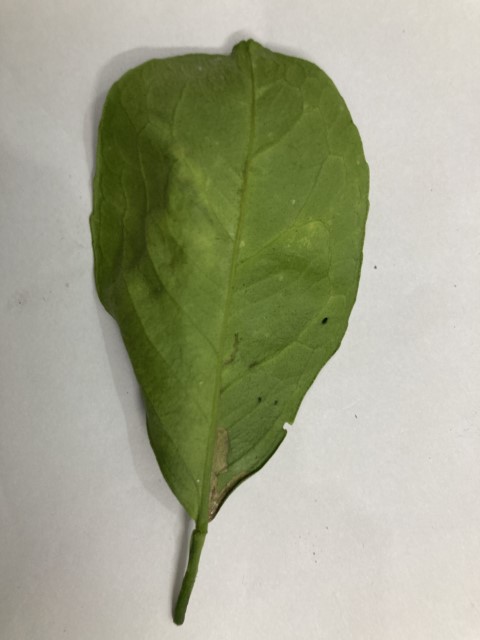

Supplement: Supplementary file 1 [file mmc1.zip › Sweetorange Sample Dataset/Converted Image/Foliage damaged/Con_Foliage Damaged637.jpeg]

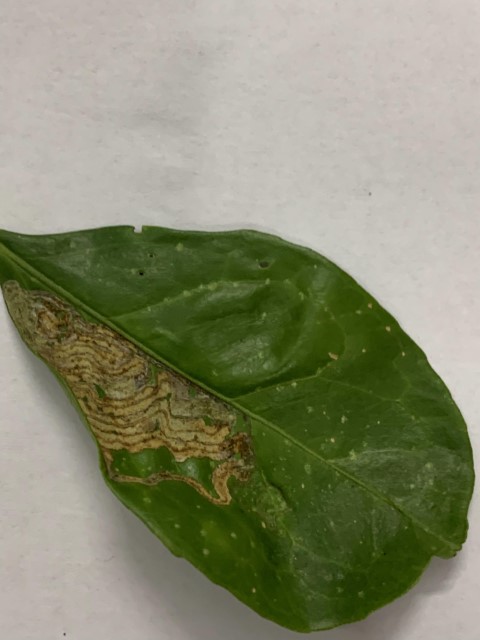

Supplement: Supplementary file 1 [file mmc1.zip › Sweetorange Sample Dataset/Converted Image/Foliage damaged/Con_Foliage Damaged35.jpeg]

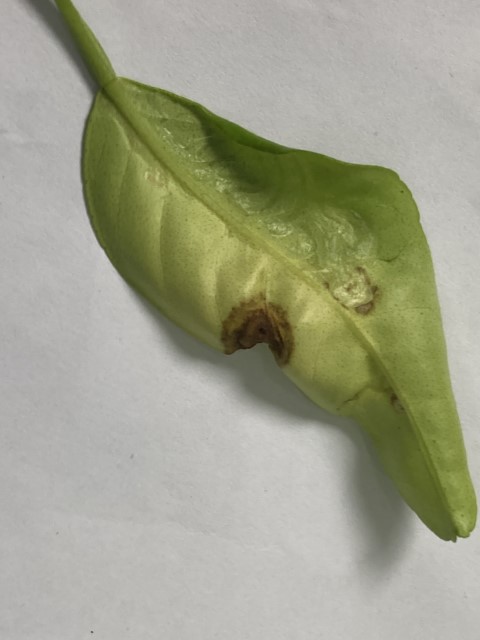

Supplement: Supplementary file 1 [file mmc1.zip › Sweetorange Sample Dataset/Converted Image/Foliage damaged/Con_Foliage Damaged154.jpeg]

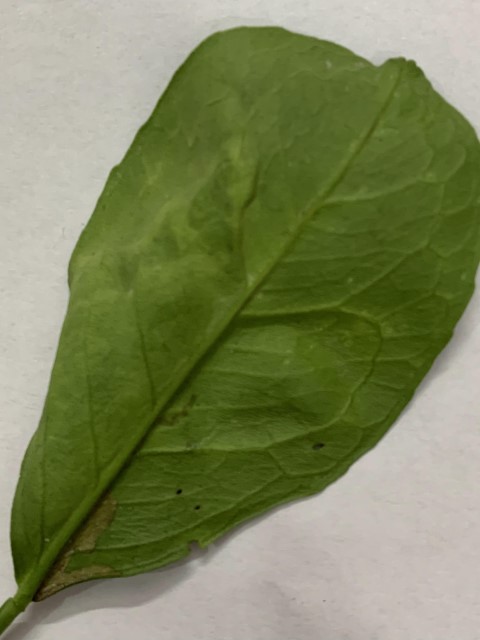

Supplement: Supplementary file 1 [file mmc1.zip › Sweetorange Sample Dataset/Converted Image/Foliage damaged/Con_Foliage Damaged42.jpeg]

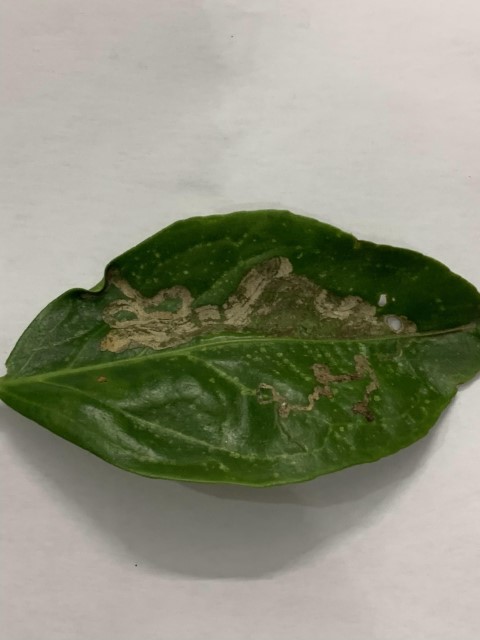

Supplement: Supplementary file 1 [file mmc1.zip › Sweetorange Sample Dataset/Converted Image/Foliage damaged/Con_Foliage Damaged14.jpeg]

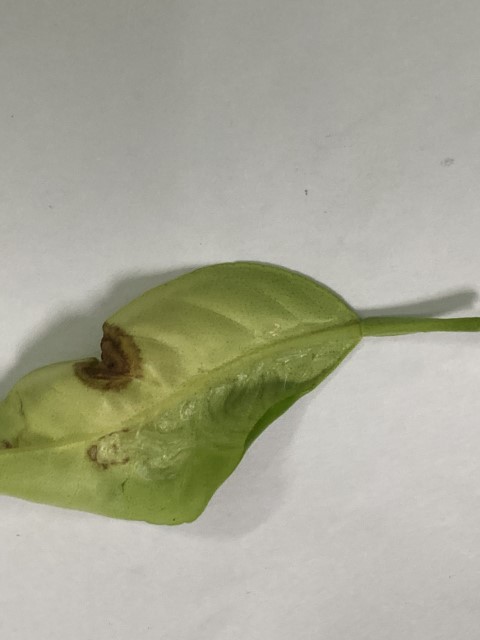

Supplement: Supplementary file 1 [file mmc1.zip › Sweetorange Sample Dataset/Converted Image/Foliage damaged/Con_Foliage Damaged175.jpeg]

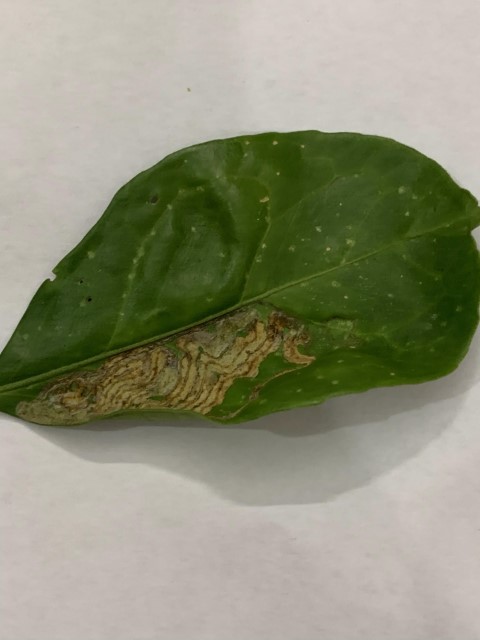

Supplement: Supplementary file 1 [file mmc1.zip › Sweetorange Sample Dataset/Converted Image/Foliage damaged/Con_Foliage Damaged63.jpeg]

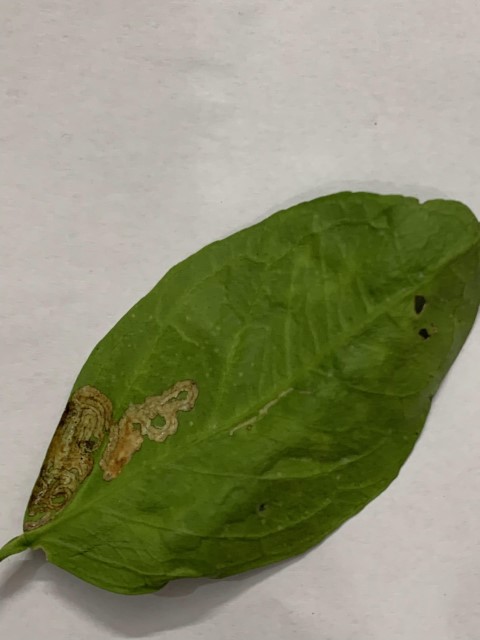

Supplement: Supplementary file 1 [file mmc1.zip › Sweetorange Sample Dataset/Converted Image/Foliage damaged/Con_Foliage Damaged21.jpeg]

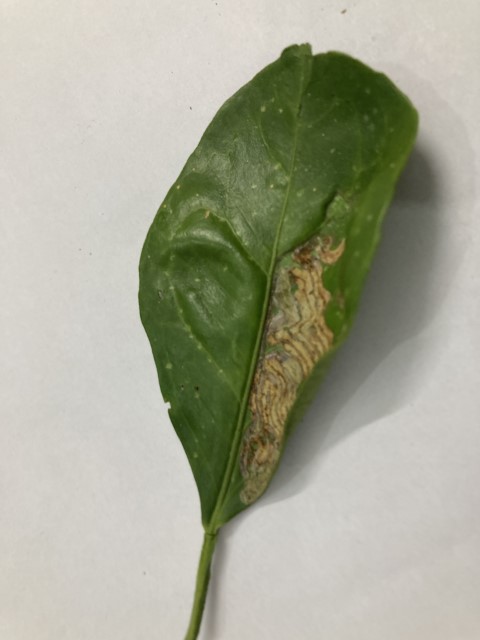

Supplement: Supplementary file 1 [file mmc1.zip › Sweetorange Sample Dataset/Converted Image/Foliage damaged/Con_Foliage Damaged623.jpeg]

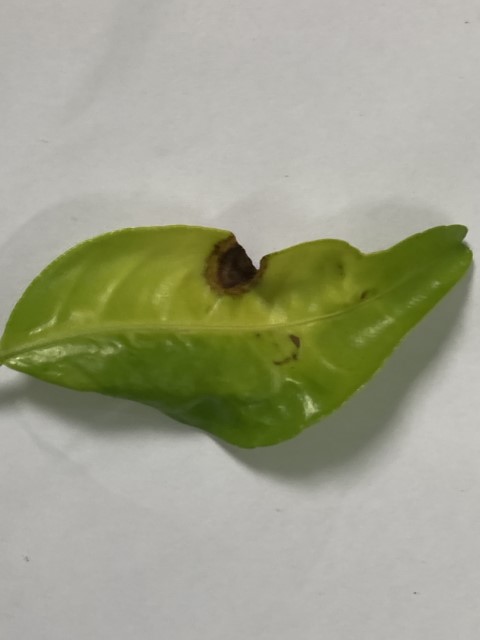

Supplement: Supplementary file 1 [file mmc1.zip › Sweetorange Sample Dataset/Converted Image/Foliage damaged/Con_Foliage Damaged140.jpeg]

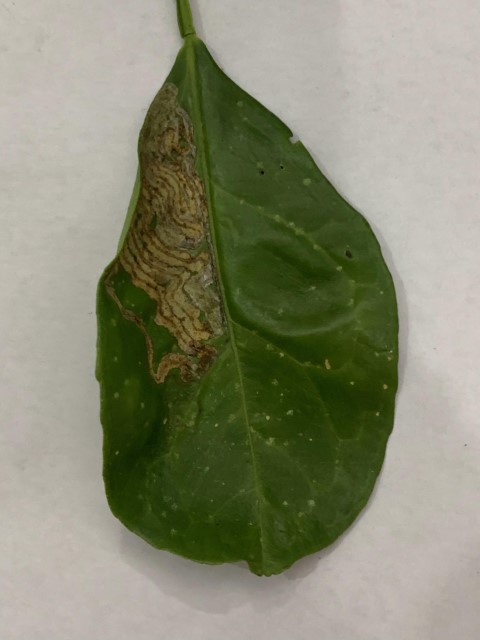

Supplement: Supplementary file 1 [file mmc1.zip › Sweetorange Sample Dataset/Converted Image/Foliage damaged/Con_Foliage Damaged56.jpeg]

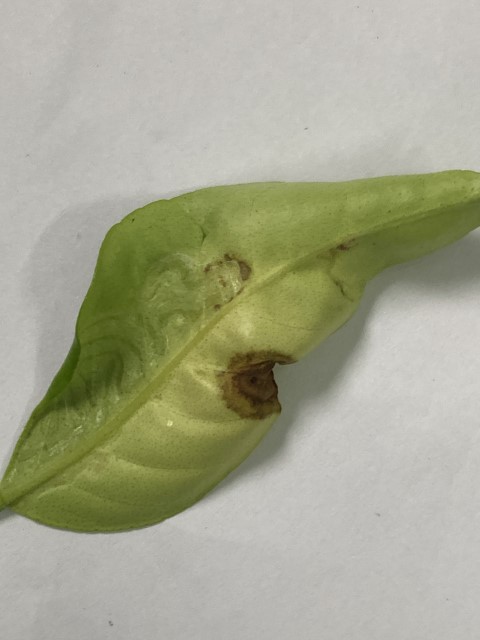

Supplement: Supplementary file 1 [file mmc1.zip › Sweetorange Sample Dataset/Converted Image/Foliage damaged/Con_Foliage Damaged161.jpeg]

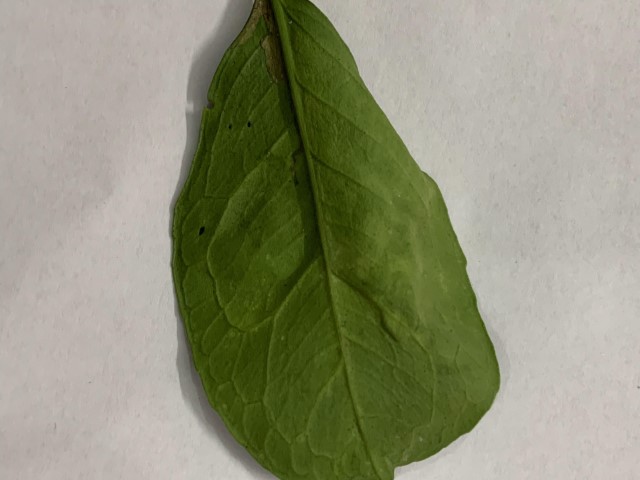

Supplement: Supplementary file 1 [file mmc1.zip › Sweetorange Sample Dataset/Converted Image/Foliage damaged/Con_Foliage Damaged77.jpeg]

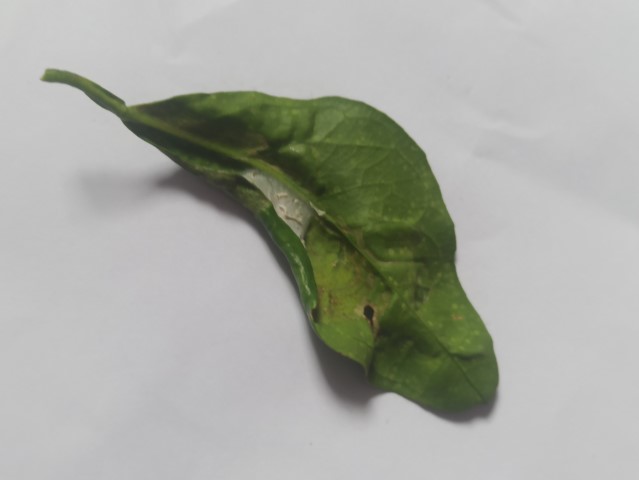

Supplement: Supplementary file 1 [file mmc1.zip › Sweetorange Sample Dataset/Converted Image/Foliage damaged/Con_Foliage Damaged644.jpeg]

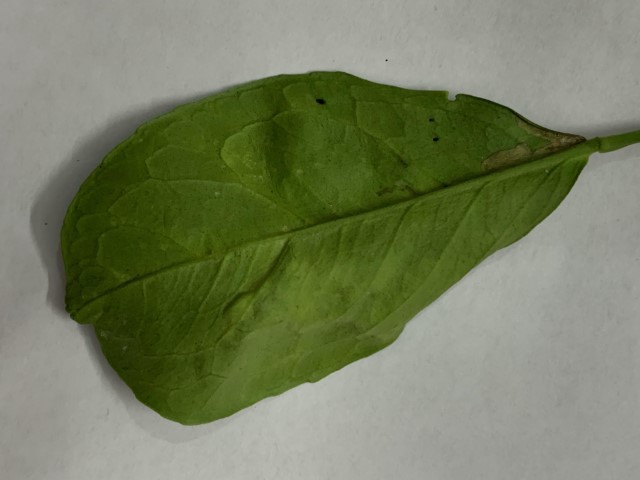

Supplement: Supplementary file 1 [file mmc1.zip › Sweetorange Sample Dataset/Converted Image/Foliage damaged/Con_Foliage Damaged70.jpeg]

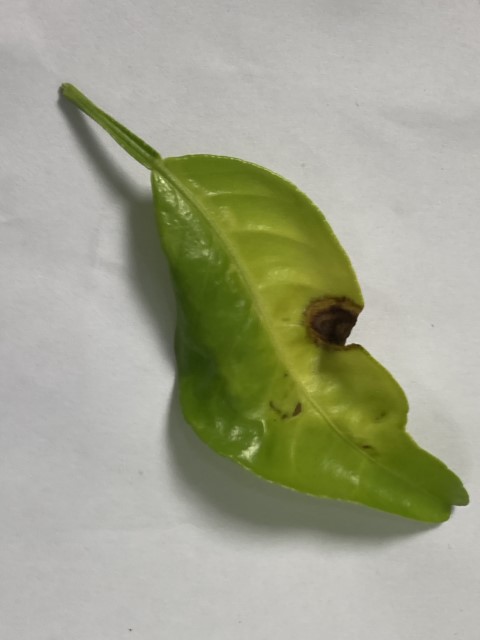

Supplement: Supplementary file 1 [file mmc1.zip › Sweetorange Sample Dataset/Converted Image/Foliage damaged/Con_Foliage Damaged147.jpeg]

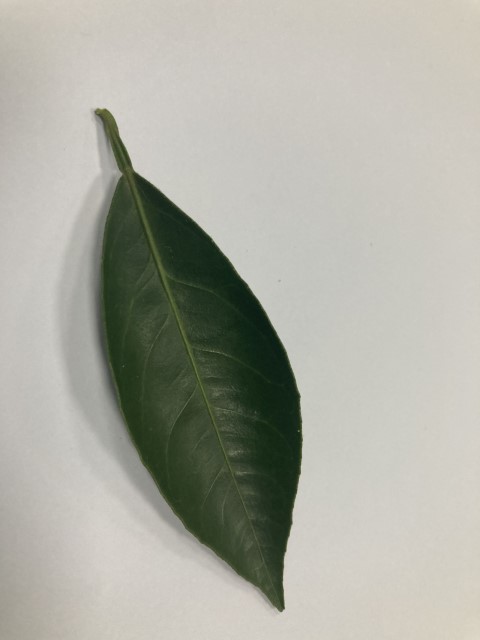

Supplement: Supplementary file 1 [file mmc1.zip › Sweetorange Sample Dataset/Converted Image/Healthy leaf/Con_Healthy Leaf49.jpeg]

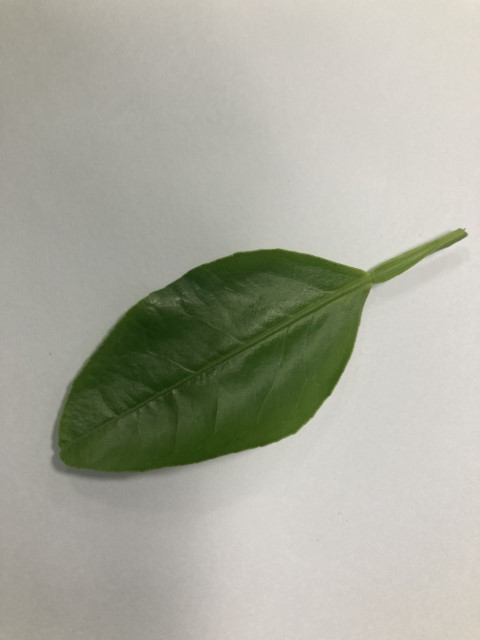

Supplement: Supplementary file 1 [file mmc1.zip › Sweetorange Sample Dataset/Converted Image/Healthy leaf/Con_Healthy Leaf28.jpeg]

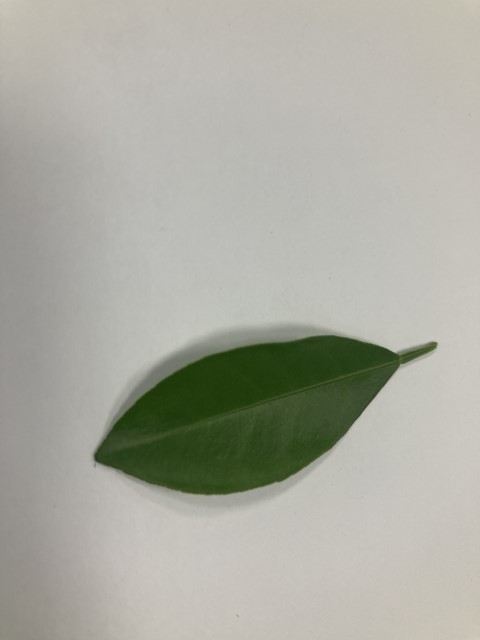

Supplement: Supplementary file 1 [file mmc1.zip › Sweetorange Sample Dataset/Converted Image/Healthy leaf/Con_Healthy Leaf189.jpeg]

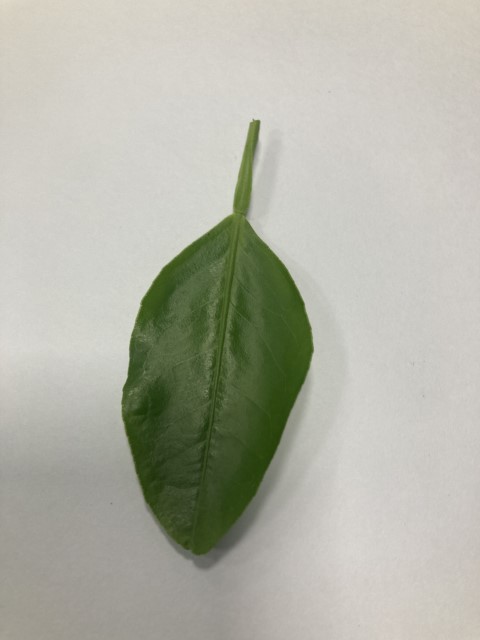

Supplement: Supplementary file 1 [file mmc1.zip › Sweetorange Sample Dataset/Converted Image/Healthy leaf/Con_Healthy Leaf14.jpeg]

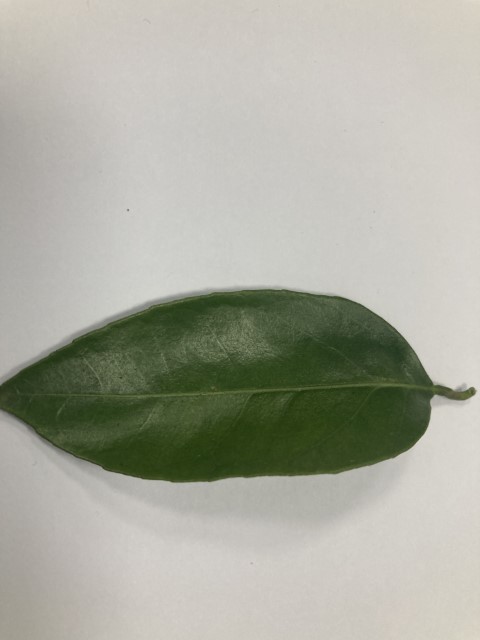

Supplement: Supplementary file 1 [file mmc1.zip › Sweetorange Sample Dataset/Converted Image/Healthy leaf/Con_Healthy Leaf588.jpeg]

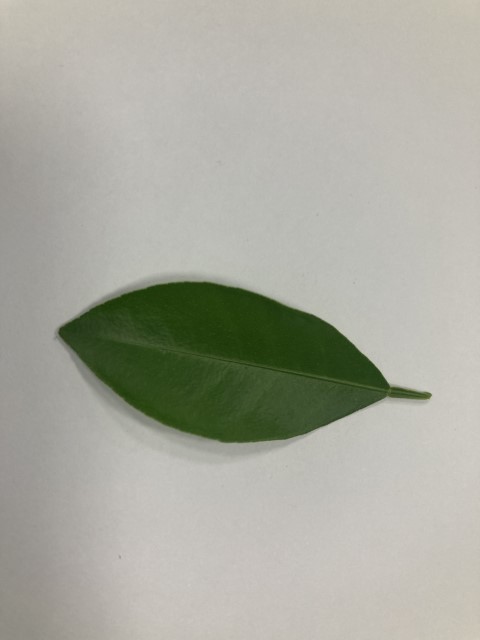

Supplement: Supplementary file 1 [file mmc1.zip › Sweetorange Sample Dataset/Converted Image/Healthy leaf/Con_Healthy Leaf182.jpeg]

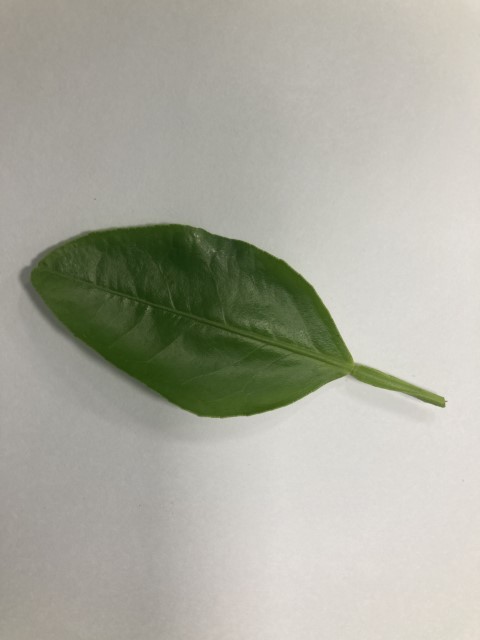

Supplement: Supplementary file 1 [file mmc1.zip › Sweetorange Sample Dataset/Converted Image/Healthy leaf/Con_Healthy Leaf35.jpeg]

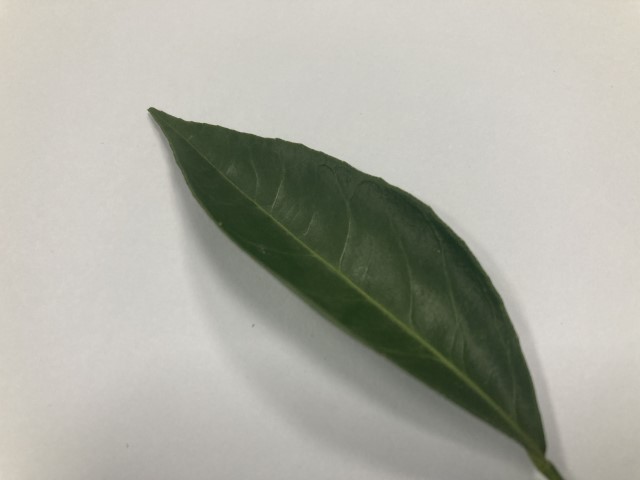

Supplement: Supplementary file 1 [file mmc1.zip › Sweetorange Sample Dataset/Converted Image/Healthy leaf/Con_Healthy Leaf161.jpeg]

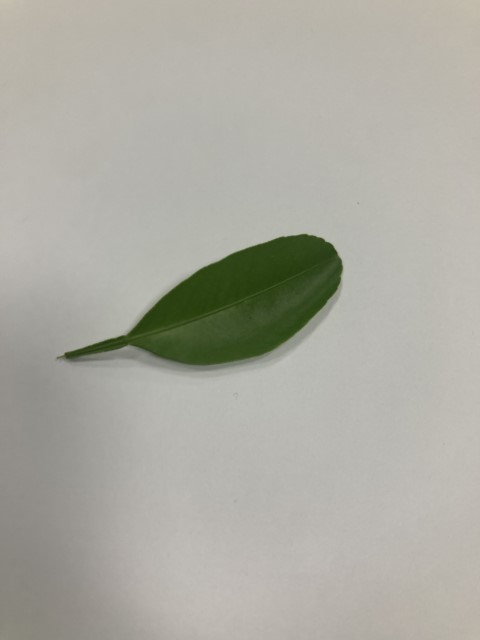

Supplement: Supplementary file 1 [file mmc1.zip › Sweetorange Sample Dataset/Converted Image/Healthy leaf/Con_Healthy Leaf42.jpeg]

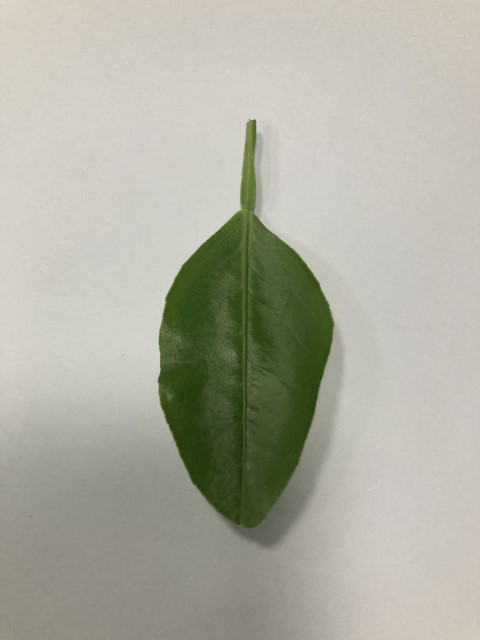

Supplement: Supplementary file 1 [file mmc1.zip › Sweetorange Sample Dataset/Converted Image/Healthy leaf/Con_Healthy Leaf7.jpeg]

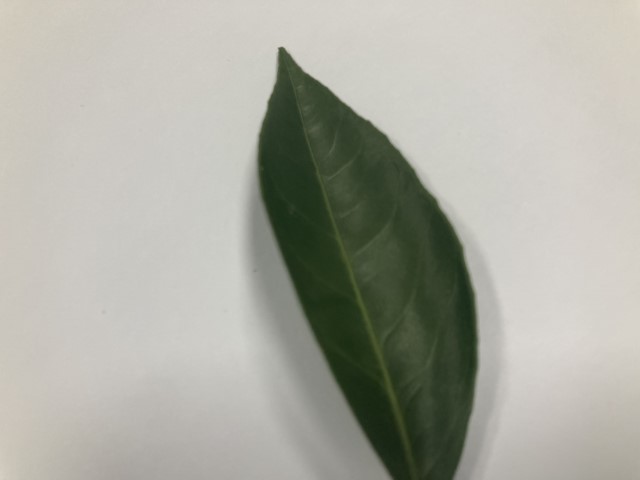

Supplement: Supplementary file 1 [file mmc1.zip › Sweetorange Sample Dataset/Converted Image/Healthy leaf/Con_Healthy Leaf154.jpeg]

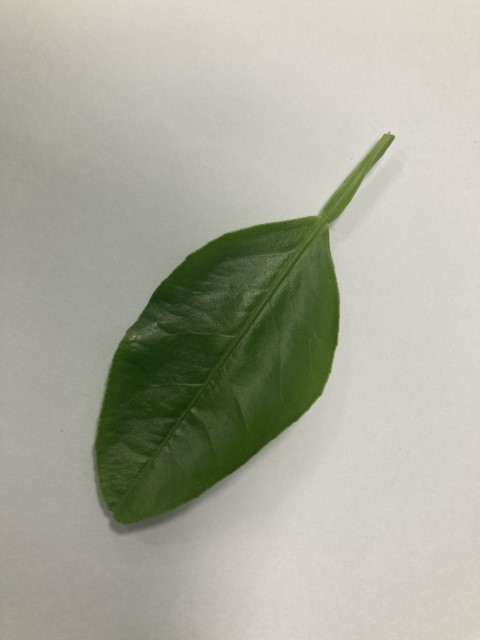

Supplement: Supplementary file 1 [file mmc1.zip › Sweetorange Sample Dataset/Converted Image/Healthy leaf/Con_Healthy Leaf21.jpeg]

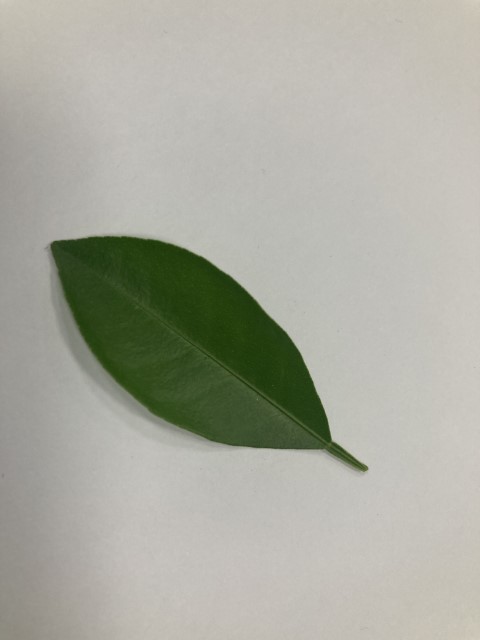

Supplement: Supplementary file 1 [file mmc1.zip › Sweetorange Sample Dataset/Converted Image/Healthy leaf/Con_Healthy Leaf175.jpeg]

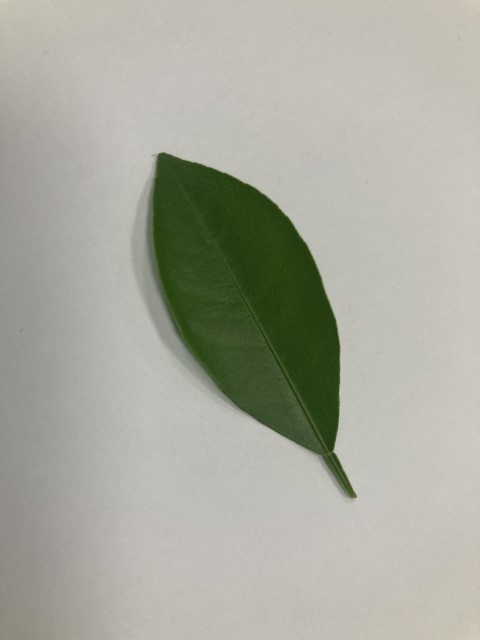

Supplement: Supplementary file 1 [file mmc1.zip › Sweetorange Sample Dataset/Converted Image/Healthy leaf/Con_Healthy Leaf168.jpeg]

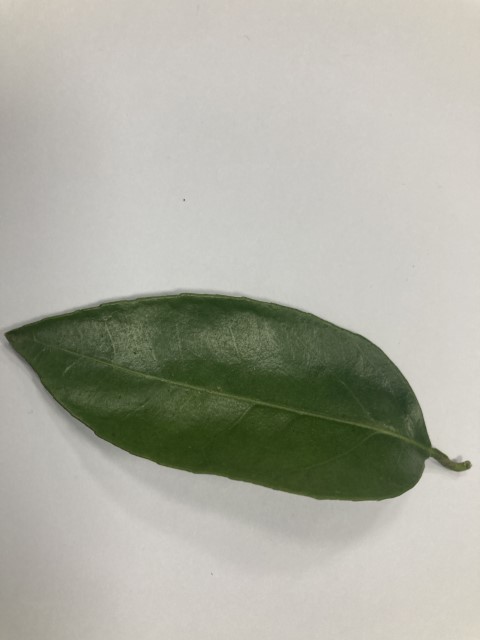

Supplement: Supplementary file 1 [file mmc1.zip › Sweetorange Sample Dataset/Converted Image/Healthy leaf/Con_Healthy Leaf574.jpeg]

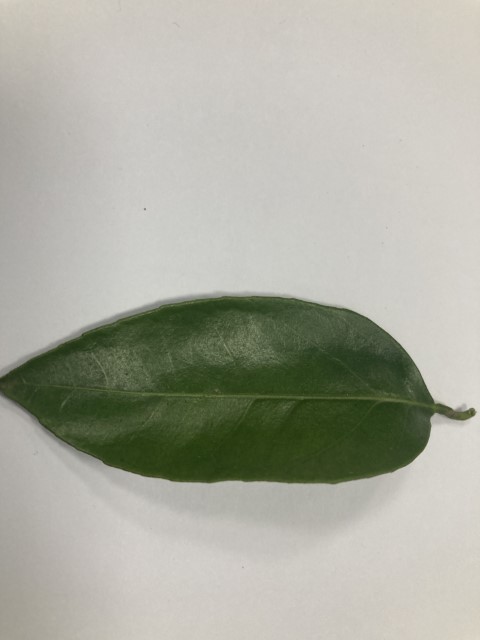

Supplement: Supplementary file 1 [file mmc1.zip › Sweetorange Sample Dataset/Converted Image/Healthy leaf/Con_Healthy Leaf581.jpeg]

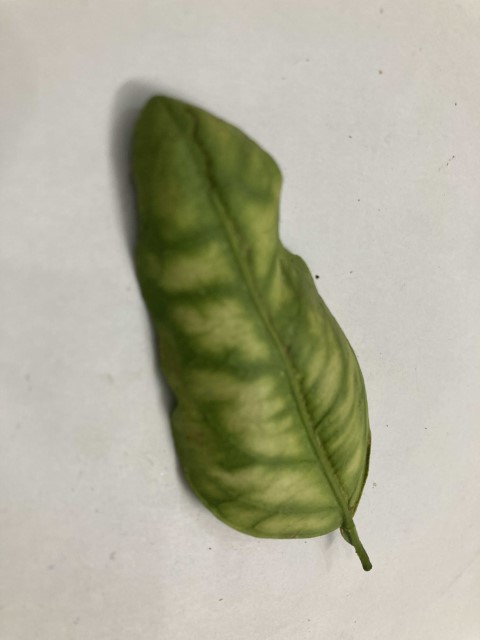

Supplement: Supplementary file 1 [file mmc1.zip › Sweetorange Sample Dataset/Converted Image/Citrus greening/Con_Citrus Greening72.jpeg]

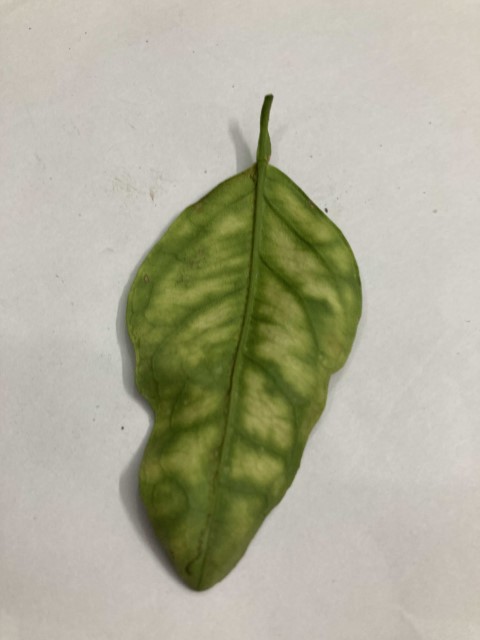

Supplement: Supplementary file 1 [file mmc1.zip › Sweetorange Sample Dataset/Converted Image/Citrus greening/Con_Citrus Greening48.jpeg]

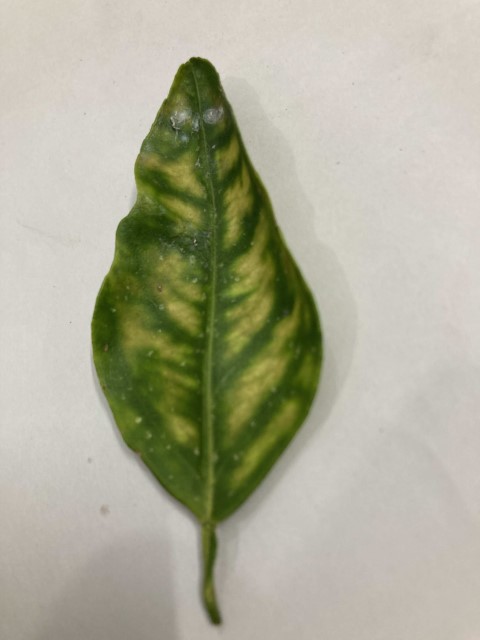

Supplement: Supplementary file 1 [file mmc1.zip › Sweetorange Sample Dataset/Converted Image/Citrus greening/Con_Citrus Greening246.jpeg]

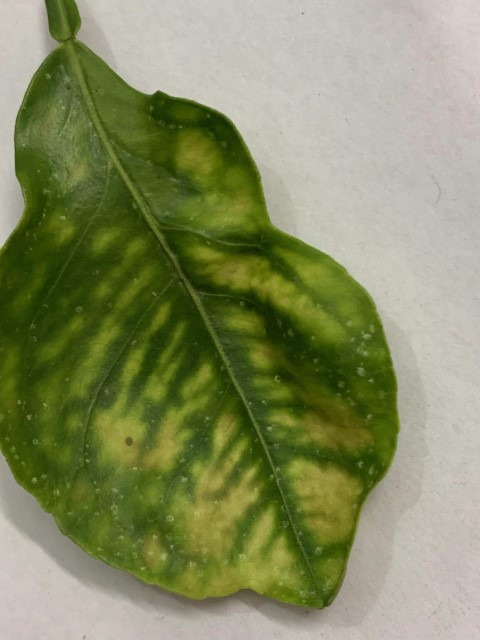

Supplement: Supplementary file 1 [file mmc1.zip › Sweetorange Sample Dataset/Converted Image/Citrus greening/Con_Citrus Greening24.jpeg]

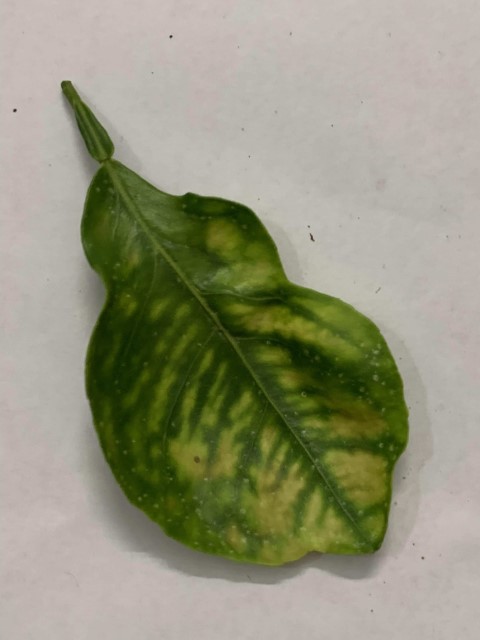

Supplement: Supplementary file 1 [file mmc1.zip › Sweetorange Sample Dataset/Converted Image/Citrus greening/Con_Citrus Greening12.jpeg]

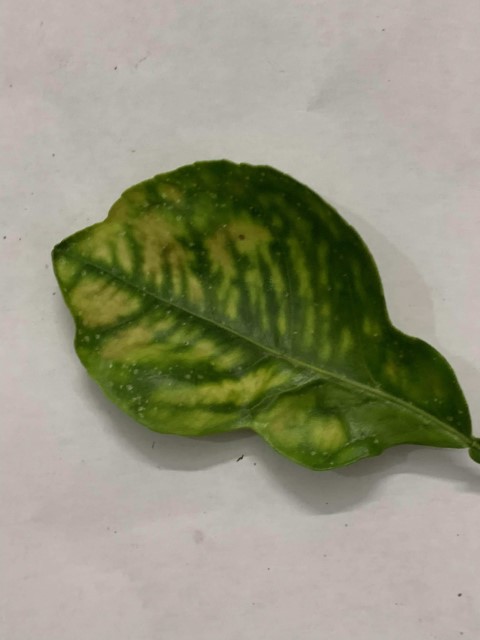

Supplement: Supplementary file 1 [file mmc1.zip › Sweetorange Sample Dataset/Converted Image/Citrus greening/Con_Citrus Greening6.jpeg]

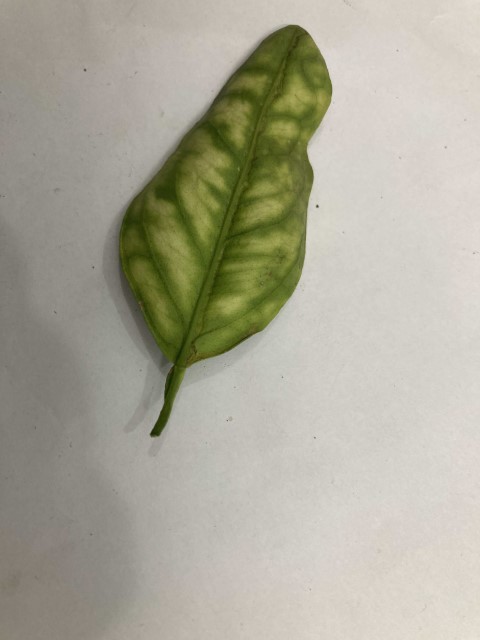

Supplement: Supplementary file 1 [file mmc1.zip › Sweetorange Sample Dataset/Converted Image/Citrus greening/Con_Citrus Greening78.jpeg]

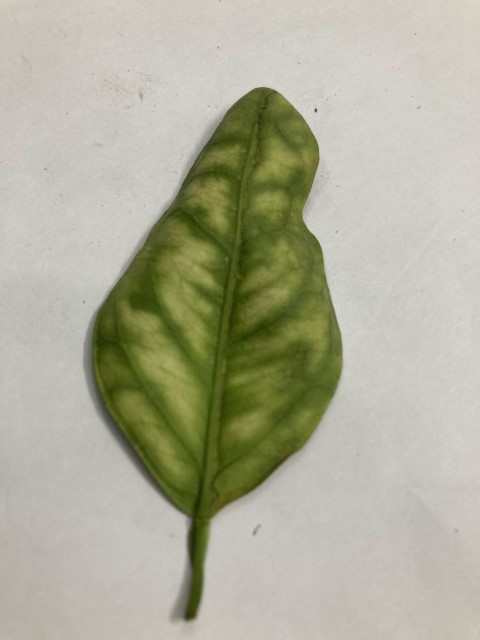

Supplement: Supplementary file 1 [file mmc1.zip › Sweetorange Sample Dataset/Converted Image/Citrus greening/Con_Citrus Greening42.jpeg]

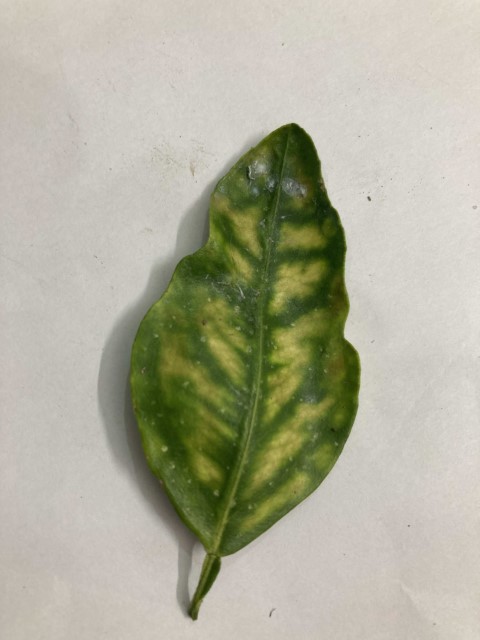

Supplement: Supplementary file 1 [file mmc1.zip › Sweetorange Sample Dataset/Converted Image/Citrus greening/Con_Citrus Greening54.jpeg]

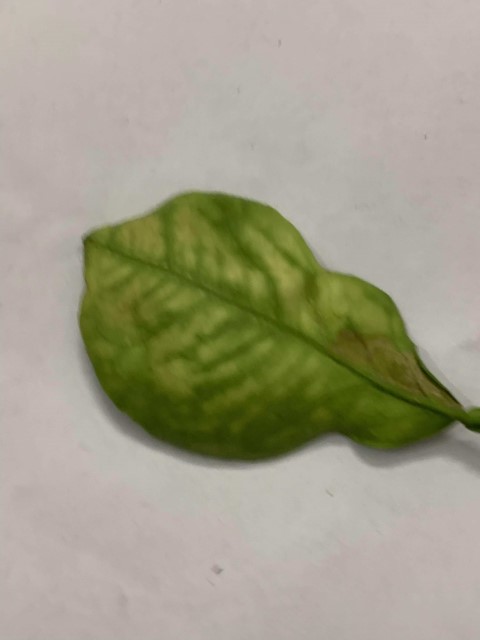

Supplement: Supplementary file 1 [file mmc1.zip › Sweetorange Sample Dataset/Converted Image/Citrus greening/Con_Citrus Greening18.jpeg]

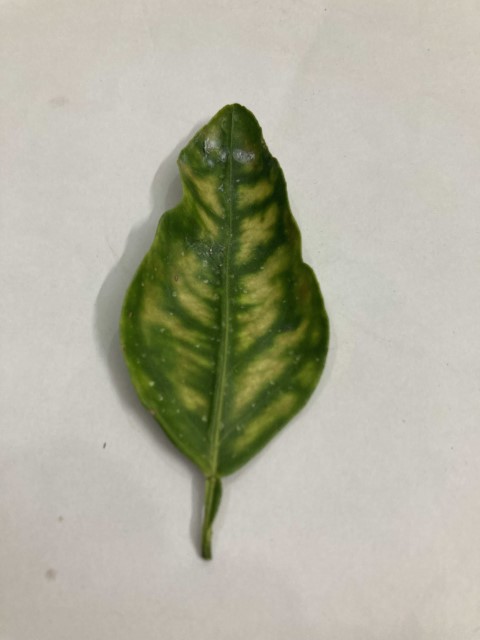

Supplement: Supplementary file 1 [file mmc1.zip › Sweetorange Sample Dataset/Converted Image/Citrus greening/Con_Citrus Greening240.jpeg]

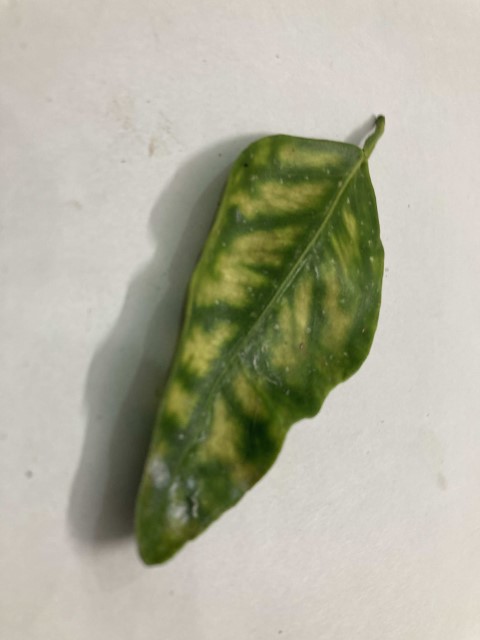

Supplement: Supplementary file 1 [file mmc1.zip › Sweetorange Sample Dataset/Converted Image/Citrus greening/Con_Citrus Greening60.jpeg]

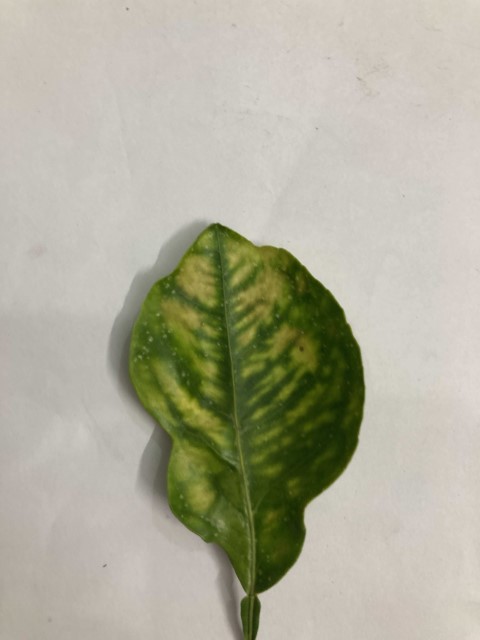

Supplement: Supplementary file 1 [file mmc1.zip › Sweetorange Sample Dataset/Converted Image/Citrus greening/Con_Citrus Greening234.jpeg]

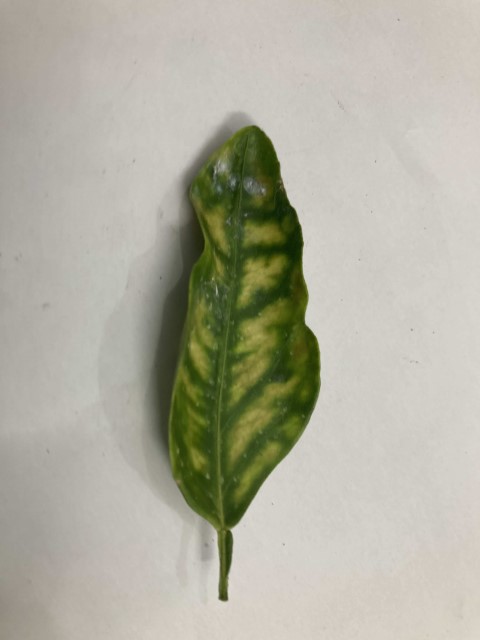

Supplement: Supplementary file 1 [file mmc1.zip › Sweetorange Sample Dataset/Converted Image/Citrus greening/Con_Citrus Greening36.jpeg]

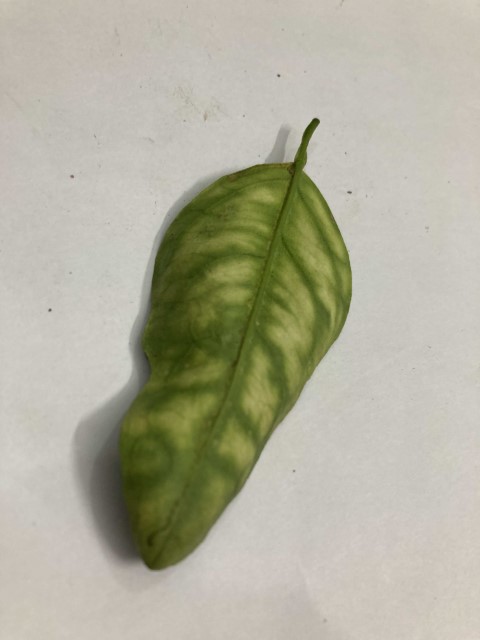

Supplement: Supplementary file 1 [file mmc1.zip › Sweetorange Sample Dataset/Converted Image/Citrus greening/Con_Citrus Greening66.jpeg]

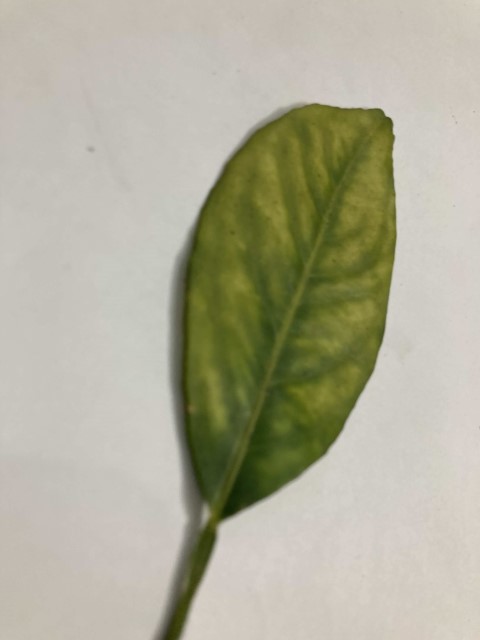

Supplement: Supplementary file 1 [file mmc1.zip › Sweetorange Sample Dataset/Converted Image/Citrus greening/Con_Citrus Greening252.jpeg]

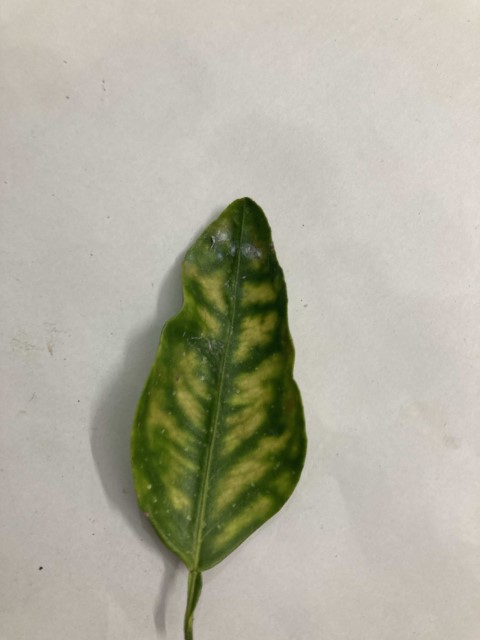

Supplement: Supplementary file 1 [file mmc1.zip › Sweetorange Sample Dataset/Converted Image/Citrus greening/Con_Citrus Greening30.jpeg]

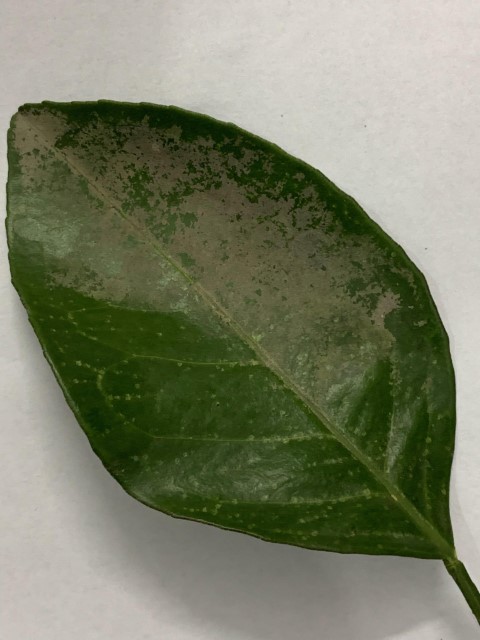

Supplement: Supplementary file 1 [file mmc1.zip › Sweetorange Sample Dataset/Converted Image/Powdery mildew/Con_Powdery Mildew7.jpeg]
